# Structural ultrasound of joints and tendons in healthy children: development of normative data

**DOI:** 10.1186/s12969-023-00895-8

**Published:** 2023-09-19

**Authors:** Ruth Wittoek, Céline Decock, Nele Dewaele, Lara Arnold, Pieter Baeyens, Ignace De Schrijver, Lisa Pardaens, Ioannis Raftakis, Thomas Renson, Charline Rinkin, Alexander D. J. Thooft, Tine Vanhaverbeke, Caroline Verbist

**Affiliations:** 1 Rheumatology, Ghent University Hospital, Ghent, Belgium; 2 Faculty of Medicine and Health Sciences, Dept. of Internal Medicine and Pediatrics, Ghent University, Ghent, Belgium; 3 Rheumatology, AZ Sint-Lucas Gent, Ghent, Belgium; 4 Radiology, Clinique de Flandre, Coudekerque-Branche, France; 5 Rheumatology, CHU-Brugmann Hospital Brussels, Brussels, Belgium; 6 RITA, European Reference Networks, Brussels, Belgium; 7 Rheumatology, CHU Liège, Liège, Belgium; 8 Rheumatology, AZ Delta, Roeselare, Belgium

**Keywords:** Ultrasound, Growth tables, Normative data

## Abstract

**Background:**

Musculoskeletal ultrasound is a well accessible technique to assess disease activity in children with juvenile idiopathic arthritis. Knowledge of reference values of joint structures is indispensable to differentiate between physiological and pathological finding. The aim of this study was to assess the structural sonographic features of joints and tendons in healthy children from several age groups (0.2–18 year), and develop a set of normative data.

**Methods:**

Greyscale ultrasound was performed in 500 healthy children (age 0.2–18 years) according to a predefined scanning protocol (Additional file 1) including the shoulder, elbow, wrist, second metacarpophalangeal joint, hip, knee, ankle, and first metatarsophalangeal joint). Demographic data and values of cartilage thickness, tendon diameters, and the degree of capsular distention measured by bone-capsular distance (BCD) were collected. Differences according to the sex were assessed by unpaired t-test. Single and multiple regression analyses were performed between the ultrasound outcomes and covariates such as age, height, weight and body mass index. Growth charts and tables were developed with respect to age. Nonparametric quantile regression was applied using the R-packages quantreg and quantregGrowth.

**Results:**

A total of 195 male and 305 female volunteers were included between the age of 0 and 18 years (mean age 8.9; range: 0.2–17.9 years). Cartilage diminished markedly as children aged, and cartilage of the boys was significantly thicker compared to the girls in all joints (p < 0.001). In addition, cartilage became thinner as children’s height and weight increased (beta regression coefficients between − 0.27 and − 0.01, p < 0.0001). Capsular distention (i.e., BCD > 0 mm) was uncommon in the ankle, wrist and MCP2 (resp. in 3, 6, and 3% of cases). It was more common in the suprapatellar and parapatellar knee, MTP1 and posterior recess of the elbow (resp. in 34, 32, 46, and 39% of cases). In the hip, some capsular distention was always present. Age was found to be the best predictor for BCD (beta regression coefficients between 0.05 and 0.13, p < 0.0001). Height was, in addition to age, a good predictor of tendon diameter (beta regression coefficients between 0.03 and 0.14, p < 0.0001). Growth curves and tables for each variable were developed.

**Conclusions:**

Reference values of sonographic cartilage thickness, BCD and diameters of tendons at several joints were established from 500 healthy children, aged between 0.2 and 18 years. Growth charts and tables were developed to distinguish normal findings from pathology in children with complaints suspicious of arthritis.

**Supplementary Information:**

The online version contains supplementary material available at 10.1186/s12969-023-00895-8.

## Background

Imaging techniques such as ultrasound (US) and magnetic resonance imaging (MRI) have made great progress in recent decades and have enabled the diagnostic process and monitoring of disease activity in patients with rheumatic diseases. Especially in the pediatric population, the availability of high end ultrasound machines and increased knowledge of pediatric ultrasound examination have provided significant advantages [1–5]. US has better accessibility and tolerability than MRI, even in very young children, without the need for sedation to avoid disturbing movement artefacts. However, several pitfalls and challenges impede the general use of US in children and interpretation of pediatric musculoskeletal US images: incomplete ossification, physiological vascularity (i.e., nutritional vessels in cartilage) and changing US anatomy of the growing skeleton during childhood [5–8]. Standard reference values have been established from a few previous studies but these mainly focused on cartilage thickness and very young children were often excluded [9–13].

Knowledge of reference values of joint structures is indispensable to differentiate between physiological and pathological findings.

The aim of this study is to develop normative data of several anatomical structures of joints (not limited to cartilage), and surrounding tissues relevant in juvenile idiopathic arthritis (JIA), that allow us to differentiate normal findings from minimal and severe abnormalities even in very young children.

## Methods

### Study design and participants

This study was an observational, cross-sectional, monocentric study in healthy children and adolescents. The study complied with the Declaration of Helsinki and approval from the local ethics committee of the Ghent University Hospital was obtained. Parents or legal guardians of the study participants provided verbal and written informed consent, and all study participants aged ≥ 12 years also provided personal consent (assent) before entering the study.

This study aimed to include 500 healthy children and adolescents aged between 0 and 18 years. The participants were divided into nine age groups to cover different stages of joint development (0–2, 2–4, 4–6, 6–8, 8–10, 10–12, 12–14, 14–16 and 16–18 years). At least 50 participants were included in all age groups.

The study was organized in a Belgian amusement park during the summer holidays of 2020. The study team and volunteers complied at all times with ongoing COVID-19 prevention and control measures prescribed by the public health authorities. Children and their parents or legal guardians visiting the amusement park were randomly approached by members of our promotional team, noncommittally informed about the project and purpose and invited to participate on a voluntary basis.

Exclusion criteria were chronic diseases, presence and/or history of any underlying inflammatory rheumatic condition or previous arthritis or surgical intervention of one or more peripheral joint(s), and intake of medicines influencing growth or bone metabolism. The occurrence of previous fractures or other joint traumata was recorded in the file. Demographic data such as sex, age, height, weight and body mass index (BMI) were recorded. These data were collected based on self-report before the ultrasound examination took place. No access to medical records was available since the participant were healthy volunteers, not patients.

### Standardized scanning protocol

An additional file shows the standardized scanning protocol including all details about positioning the child and probe, and anatomic landmarks that was developed and followed during the entire US procedure (see Additional file 1). The following joints were included: shoulder, hip, knee, ankle, first metatarsophalangeal joint (MTP1), elbow, wrist, and second metacarpophalangeal joint (MCP2). Only unilateral (right sided) joints were assessed (except in case of antecedent of traumata or other events that could influence normal maturation of this joint), to keep the examination within the anticipated time limits and since no left right difference in healthy children was demonstrated earlier [9, 14]. The following anatomical structures were assessed according to EULAR guidelines [15]: the bone-capsular distance (BCD) at the acetabulofemoral, supra- and parapatellar, tibiotalar, MTP1 joint (dorsal side), lateral and anterior radiohumeral, posterior fossa of the elbow, radio-lunate, lunate-capitatum and capitatum-metacarpal 3, and MCP2 joint (dorsal side) recesses; the diameter of tendons of the biceps (proximal part), extensor digitorum communis, extensor carpi ulnaris, flexor digitorum of the 2nd finger, and patellar tendon; and cartilage thickness at the femoral head, the trochlea of the knee, the talar dome, the head of the 1st metatarsal bone (dorsal side) and 2nd metacarpal bone (dorsal side). Standard scans were chosen [15], except for the ankle joint. For BCD, the greatest perpendicular distance between the inner capsular layer and underlying bony surface was measured (Additional file 2, p. 6). High-resolution US machines were used allowing to align the joint capsule or differentiate from adjacent structures such as tendons/tendon sheets or connective tissue. For tendon diameters, the greatest diameter was measured in the transverse view (Additional file 2, p. 6). For cartilage, the greatest thickness perpendicular to the bony surface was measured, including the hyaline cartilage layer (this was chosen since preliminary observations showed that this considerably improved the reliability of this measure)(Additional file 2, p. 6) [16]. All measurements were performed in millimeters (mm). The use of predefined settings was promoted but the sonographer was given the liberty to adjust settings to achieve the best quality image. All measurements were performed in real time, and pictures were digitally stored. The entire scanning time was anticipated to be kept below 30 min.

Only greyscale US was performed. Several high-resolution US machines were used with 8 to 18 MHz linear transducers: MyLab7® (Esaote, Genua, Italy) and LOGIQ S8® (GE Healthcare, Chicago, USA).

### Sonographers and interobserver reliability

Six sonographers (4 rheumatologists and 2 radiologists) with a high level of experience (> 10 years) in musculoskeletal US and 4 junior sonographers (< 2 years of experience) performed all ultrasounds. Intensive training sessions took place and the standardized protocol was studied before recruiting the children. An interobserver reliability study took place among the junior sonographers where all structures were scanned independently by the sonographers in a 6 year-old child during the same session. An interobserver reliability study based on independent readings of acquired images of children was performed by the experienced sonographers.

### Statistical analysis

Descriptive analysis was performed on the demographic and sonographic data. Missing data were not replaced. If not all joints or structures of a participant were scanned (due to loss/lack of cooperation of the child), the respective missing measurements were considered missing data. For reliability analyses, the intraclass coefficient of correlation (ICC) was calculated for average measures. In the overall population, normal distribution of data was assumed. The central limit theorem was applied.

Comparison between boys and girls were analyzed using unpaired t-test with the level of significance (α) set at 0.05. These analyses were performed in IBM® SPSS™ statistics version 27 (New York, USA).

For each location of interest and for each of the measures (tendon diameter, cartilage thickness and BCD) simple linear regression was performed separately for boys and girls with the following predictors: age (in years (continuous)), height(in centimeter (cm)), weight (in kilogram (kg)), and BMI (kg/m²). Moreover, multiple linear regression was applied with the following combinations of predictors: age and height; age and weight; and age, height and weight. The estimated regression coefficients were reported together with the corresponding p-value and the adjusted R squared (adjustment for the number of predictors in the model). Since a linear relationship could not be assumed for the BCD of most of the joints (since > 50% null values), only regression analyses at acetabulofemoral recess and recess of MTP1 were performed.

Growth charts are used to build reference values of the outcomes of interest with respect to age. Nonparametric quantile regression with a penalty on the coefficients and an estimated smoothing parameter was applied using the R packages quantreg and quantregGrowth [17]. For tendons, monotonicity was assumed: nondecreasing growth curves were fitted. In general, confidence intervals for the quantiles are expected to be wider at the lower and upper ends of the growth curve since here, data on only one side are available to estimate the quantiles in that region.

## Results

### Study participants

In total, 500 healthy children between 0.2 and 18 years old were included, and over 4000 joints were assessed. Demographic data are depicted in Table 1. Overall, more females were included. All age groups were well balanced concerning gender, expect for the 14 to 16 years age group, in which females are overrepresented.

**Table 1:** Demographic data of volunteers (n = 500) across different age categories

Age category	N (female (%))	Age (years)	Height (cm)	Weight (kg)	BMI (kg/m²)
0–2	54 (56)	1.1 (0.2-2.0)	76 (60–92)	10 (6–16)	16.9 (11.9–22.5)
2–4	58 (55)	3.2 (2.0–4.0)	99 (83–120)	16 (11–23)	15.8 (10.7–21.7)
4–6	59 (59)	5.1 (4.0–6.0)	112 (100–125)	20 (11–31)	15.8 (10.5–20.3)
6–8	54 (56)	6.9 (6.0–8.0)	123 (110–137)	24 (14–39)	15.5 (11.3–23.8)
8–10	58 (64)	9.0 (8.0–10.0)	135 (120–148)	30 (21–50)	16.1 (12.6–23.8)
10–12	56 (55)	11.0 (10.0-11.9)	146 (125–164)	39 (25–68)	18.0 (12.8–26.2)
12–14	57 (61)	13.0 (12.0–14.0)	160 (140–182)	48 (28–74)	18.7 (13.7–27.3)
14–16	54 (78)	14.9 (14.0-15.9)	167 (150–183)	56 (38–78)	19.9 (16.2–27.9)
16–18	50 (66)	16.9 (16.0–18.0)	76 (160–192)	65 (42–103)	22.4 (17.0-35.6)

Data are mean (range) unless otherwise stated; N = number; BMI: body mass index (weight/height²)(in kg/m²)

### Interobserver reliability

Interobserver reliability was excellent among both junior and experienced sonographers (all ICC average measures = 0.99 (95% C.I. 0.98–0.99).

### Sonographic structural characteristics

Descriptive data (median and minimum and maximum) of all measures according to the scanning protocol are shown in Table 2. All findings are illustrated in an atlas showing age related findings at all locations assessed (see Additional file 2).

**Table 2:** Descriptive data of the anatomical structures assessed

Anatomical structure	N	Median [minimum - maximum]
Cartilage*		
Femoral head	488	2.5 [0.4–10.3]
Trochlea knee	496	3.0 [1.4–5.8]
Talar dome	494	1.5 [0.1–6.1]
Head of 1st metatarsal bone	489	1.8 [0.4–7.3]
Head of 2nd metacarpal bone	489	1.5 [0.5–6.9]
*Bone-capsular distance#*		
Acetabulofemoral recess	485	3.4 [0.9–9.1]
Suprapatellar recess	489	0.0 [0.0–11.6]
Parapatellar recess	461	0.0 [0.0–3.2]
Tibiotalar recess	495	0.0 [0.0–3.7]
Recess MTP1 joint	494	0.0 [0.0–4.1]
Lateral radiohumeral recess	495	0.0 [0.0–0.8]
Anterior radiohumeral recess	495	0.0 [0.0–4.2]
Posterior fossa elbow	493	0.0 [0.0–5.1]
Radiolunate recess	473	0.0 [0.0–2.8]
Lunate-capitate recess	472	0.0 [0.0–1.5]
Capitate-metacarpal 3 recess	473	0.0 [0.0–1.8]
Recess MCP2 joint	492	0.0 [0.0–1.8]
*Tendons†*		
Biceps tendon	497	5.0 [1.5–9.3]
Patellar tendon	497	22.3 [2.9–40.6]
Extensor digitorum communis tendon	489	10.7 [3.4–24.6]
Extensor carpi ulnaris tendon	481	6.1 [2.0–11.5]
Flexor digitorum (superficial and profound) tendon of the 2nd finger	484	6.4 [2.7–10.8]

All measures are in millimeters (mm). N = number; MCP2: 2nd metacarpophalangeal joint; 1st MTP: first metatarsophalangeal joint

*: the maximal thickness of the cartilage layer was measured (incl. hyaline cartilage and cartilage surface if visible), perpendicular to the bone cortex

#: the maximal distance between the capsular layer and underlying bone cortex was measured

†: the maximum diameter of the tendon in the transverse view was measured, including the synovial sheet if present

#### Cartilage

Upon aging, cartilage size consistently decreased in all joints (Additional file 3, table S2 and tables S5-14). A significant difference in cartilage thickness was observed between sexes with boys consistently showing thicker cartilage than girls (all p < 0.001) (Table 3). Negative correlations were found between cartilage thickness at all locations and age (model 1), height (model 2), weight (model 3), and BMI (model 4) in single regression analysis, both for girls and boys (See Additional file 3, Table S2). After adjusting the models based on the children’s age and height (model 5), age and weight (model 6) and all three variants (model 7), the goodness of fit generally did not improve. Age and height, not weight, were the best predictors of cartilage thickness.

**Table 3:** Cartilage thickness at specific locations categorized by gender

Cartilage location	Boys		Girls		p value
	N	Mean* (SD)[range]	N	Mean* (SD)[range]	
Femoral head	189	3.1 (1.63)[0.8–10.3]	299	2.5 (1.41)[0.4–7.6]	< 0.001
Trochlea knee	194	3.4 (0.75)[1.8–5.8]	302	2.8 (0.72)[1.4–5.3]	< 0.001
Talar dome	192	1.8 (0.74)[0.6–6.1]	302	1.5 (0.61)[0.1–4.7]	< 0.001
Head of 1st metatarsal bone	189	2.3 (1.03)[0.4–7.3]	300	1.8 (0.87)[0.4–5.8]	< 0.001
Head of 2nd metacarpal bone	188	2.1 (1.26)[0.7–6.7]	301	1.7 (1.01)[0.5–6.9]	< 0.001

*All measures are in millimeters (mm). N = number; SD = standard deviation

#### Bone capsular distance

In general, no changes in BCD were observed between sexes. Therefore, no distinction was made between gender in further analyses or for the development of growth curves. Specifically, some capsular distention (BCD > 0 mm), not necessarily representing synovial effusion or synovitis, was always present at the acetabulofemoral recess (mean (SD)[range] = 3.6 mm (1.5)[0.9–9.1 mm]) (Table 2). Capsular distention was rare (< 3%) at the tibiotalar recess, lateral radiohumeral recess, all recesses of the wrist and MCP2 joint. At the supra- and parapatellar recesses, MTP1 joint, and the anterior radiohumeral recess and posterior fossa in the elbow, some capsular distention was often detected in healthy children (ranging from 10.5 to 45.1% of all children) (See Additional file 3, table S1). Age was found to be the best predictor for BCD at the hip and MTP1 joint, and models combining other predictors did not outperform the age model (model 1, See additional file 3, table S3). With every child’s increase in age of 1 year, the BCD increased on average by 0.08 mm in boys and 0.05 mm in girls at the recess of the MTP1 joint (p < 0.0001). In the acetabulofemoral recess, this was 0.09 and 0.13 mm in boys and girls respectively (p < 0.0001).

#### Tendons

In general, the diameter of the tendons and surrounding synovial sheets, if present, increased with aging at all locations. No difference in tendon diameter between boys and girls was present (p > 0.05). No further distinction between genders was made for the development of growth curves. Positive correlations were found between tendon diameter and age, height, weight and BMI (see Additional file 3, table S4). From the multiple regression analyses, model 4, combining age and height generally better predicted tendon diameter than the other models.

### Growth curves

Based on all data available, growth tables and growth charts with percentiles were generated for all variables (See Additional file 3, tables S5-S21 and figures S1-S26).

## Discussion

This study reports the sonographic values of several anatomical structures of joints and tendons from a large cohort of healthy children (n = 500) across several age groups from 0.2 to 18 years. To the best of our knowledge, this is the first study that included a systematic scanning protocol of several peripheral joints including measures of both cartilage thickness and degree of capsular distention, measured by BCD as well as tendon diameters, even in very young children. This dataset complements missing elements in already existing reference data.

Our data confirmed what is already known about gender specific differences in cartilage, i.e., boys have consistently thicker cartilage in all joints compared to girls [9–14]. Height and age seem to be the strongest predictors of cartilage thickness. For the hip, this was already shown before [18]. Throughout growth, the cellular concentration becomes progressively less, and in the adult hyaline articular cartilage, chondrocytes constitute less than 2% of the total volume, with more than 70% composed of water [19]. Our data were not able to explain the reason beyond this consequent gender difference: the role of sex hormones has been suggested to play a role (estrogen and testosterone receptors present on chondrocytes) but the difference is consequently being present even in prepubertal stages implying that there must be additional factors playing a role.

Our study did not show changes in BCD between genders. This corroborates evidence from previous studies [12, 18, 20]. Other studies suggested gender differences in favor of boys for the elbow joint [11]. In accordance with previous studies of other joints, such as the shoulder, elbow and hip [11–13], only a weak correlation between age and BCD was found in the MTP1 joint. Here, this might suggest an important role of mechanical loading which is of interest in future studies.

Our study found some capsular distention at the acetabulofemoral recess in every child with a mean value of 3.6 mm across all ages. The study of Collado et al. showed a mean capsular distention of 5.2 mm [20]. In both studies, the hip was scanned similarly, but in the latter study, measurements included the outer layer of the capsule, while we measure from bone to the inner layer. Previous research revealed that measuring BCD and assessing capsule shape in the hip is very prone to positioning of the joint (neutral versus external rotation) [21]. Our findings underscore that caution is warranted when applying hip US in children to avoid the overdiagnosis of arthritis.

In our study, some distention (i.e., BCD > 0 mm) of the suprapatellar and parapatellar recess of the knee, was present in 33% and 26%, respectively, which was less than previous studies reporting up to 60% [20, 22]. In these latter studies, the knee was assessed with the leg in extension instead of 15–30° degrees of flexion in our study. The maximum values reported in both studies are, however, comparable (7.3 mm in the 16-18-year age group in our study and 6 mm in the same age group by Windschall et al.) [22].

We found limited capsular distention in the anterior radiohumeral recess in 10.5% of the participants, in line with Trauzeddel et al. who reported a convex capsular shape in 7% of healthy children [11]. Chauvin et al. did not find any capsular distention at the elbow and suggested that fluid here is pathological [23].

In wrist joints, capsular distention is quite rare in healthy children: we found some distention in 1.3% at the midcarpal recesses. Rosendahl et al. reported radiocarpal and midcarpal recesses to be visible in 51.7% and 29.3%, respectively, and even bulging in 9.5% and 17.2%, respectively, of 116 healthy children [24]. No differences between genders were found in the latter [11], as confirmed in our study. Interestingly, from an age and BMI-matched study in adults, females seem to have higher quantitative and semiquantitative ultrasound measurements of synovial hypertrophy and capsular distention than males [25]. This accounts for wrist joints but also several finger, hip, ankle and toe joints.

As expected, the diameter of the tendons increased with age, which was already demonstrated in earlier studies [20, 23]. Our study demonstrated that height, in addition to age, was an important predictor of tendon diameter. No differences in gender were shown.

Heterogeneity in results among previous studies and this current study could be explained by differences in sample size, scanning technique or positioning of the study participant and especially definition of what and how to measure. We used the EULAR definitions [15], which is an advantage of the study. However, to assess capsular distention, BCD was measured from the bone to the inner layer of the synovial membrane instead of the outer layer, therefore not including the thickness of the synovial membrane itself.

Another advantage is that a wide range of children were included: from infants to adolescents almost approaching adulthood. Additionally, an extensive scanning protocol was applied to all healthy participants providing a large collection of data of almost all joints potentially affected by JIA (except for the temporomandibular joint).

Both junior and senior sonographers were involved in this project and performed the ultrasound examinations. Training sessions were organized and reliability exercises preceding the actual study demonstrated that US is a reliable exam even in the hands of junior sonographers after a short but intensive training session.

A potential limitation that might jeopardize the generalizability of the study results is that the majority of the children were of Caucasian origin (> 98%). Ethnicity might play a role in growth and joint development. There did not seem to be a difference in cartilage thickness between Caucasian and Asian children [14], but differences in height and weight of children impedes extrapolation to other ethnicities. No systematic enquiry about the intensity of daily sports activities or mechanical loading or pubertal stage was performed and might be a confounder for some of the findings. Moreover, this information might be of interest to clarify gender differences.

We only assessed right-sided joints to keep the scanning time within acceptable limits for young children. However, except for one study that showed slight differences in knee cartilage [10], there does not seem to be any influence of right-sided or left‐sided dominance on articular cartilage thickness or capsular distention. However, this aspect needs to be explored in future larger studies.

There is an underrepresentation of boys, especially in young adolescents, although findings about cartilage thickness and capsular distention are in line with previous studies [9–14].

This study reports the results of a large cohort of healthy children where several joints and relevant structures are measured in a systematic way. Growth tables and charts became available to consult whenever a sonographer might be in doubt about an ultrasound image showing unclear or unexpected values related to the age group to which the child belongs. Both very young children and adolescents almost approaching adulthood were included. In the next step, these reference values should be validated in children with JIA with active disease at several joints. There is always the possibility of anatomic variants being present, hampering these data to be applied as a universal rule.

## Conclusions

This set of normative data enabled the development of growth charts and reference data for children of all age groups and is especially of interest to the pediatric rheumatology community performing US in children with suspicious complaints to assess whether the measured values fall within the reference interval of a healthy, age-matched child. This could help accelerate the diagnosis of JIA. Cartilage is becoming thinner as children age, with consistently thicker cartilage in males. Capsular distention is rare in most joints, with the exception of the hip joint. The latter should be taken into consideration when performing ultrasound in order to avoid overdiagnosis of hip arthritis.

### Electronic supplementary material

Below is the link to the electronic supplementary material.

Additional file 1: Ultrasound scanning study protocol: this document shows the ultrasound scanning protocol of the study that enables sonographers to perform the data collection in a standardised way. This includes all details about which joint and what structures to measure, how and where to measure, how to position the child and the joint, and which frequency to use.

Additional file 2: Supplemental data: includes supplemental table S1 (prevalence of capsular distention), supplemental table S2 – S4 (multiple regression analyses), growth tables (supplemental table S5 – S 21) and growth charts (supplemental figures S1- S26).

Additional file 3: Illustrative atlas (pdf): this file includes images of all joints and structures assessed, over the different age groups. This enables to better appreciate the changes in the growing joint.

## Data Availability

The datasets used and/or analysed during the current study are available from the corresponding author on reasonable request.

## Abbreviations

BCD: Bone-capsular distance
BMI: Body mass index
CI: Confidence interval
Cm: Centimeter
EULAR: European League against Rheumatism
ICC: Intraclass coefficient of correlation
Kg: Kilogram
JIA: Juvenile idiopathic arthritis
MCP2: Second metacarpophalangeal joint
MHz: Megahertz
Mm: Millimeter
MRI: Magnetic resonance imaging
MTP1: First metatarsophalangeal joint
N: Number
SD: Standard deviation
US: Ultrasound
