# Supplementary material for: Structural ultrasound of joints and tendons in healthy children: development of normative data
Source: Pediatr Rheumatol Online J. 2023 Sep 19;21:105. doi: 10.1186/s12969-023-00895-8 (PMC10508001; doi:10.1186/s12969-023-00895-8)
Supplement: Supplementary file 1 — Additional file 1: Ultrasound scanning study protocol: this document shows the ultrasound scanning protocol of the study that enables sonographers to perform the data collection in a standardised way. This includes all details about which joint and what structures to measure, how and where to measure, how to position the child and the joint, and which frequency to use. [file 12969_2023_895_MOESM1_ESM.pdf]

Standardized scanning protocol

1. **Shoulder** biceps *transverse*
2. **Shoulder** distal biceps *transverse*
3. **Hip** acetabulofemoral recess
4. **Hip** femoral head cartilage
5. **Knee** suprapatellar recess
6. **Knee** parapatellar recess
7. **Knee** patella tendon *transverse*
8. **Knee** max. flexion cartilage
9. **Ankle** tibiotalar recess
10. **Ankle** cartilage talar dome
11. **MTP1** capsular distention + **MTP1** head cartilage
12. **Elbow** lateral radiohumeral recess
13. **Elbow** anterior radiohumeral recess
14. **Elbow** posterior fossa
15. **Wrist** radio-lunate-capitate-metacarpal recess
16. **Wrist** extensor digitorum tendons
17. **Wrist** extensor carpi ulnaris tendon
18. **MCP2** dorsal capular distention
19. **MCP2** flexion cartilage head
20. **MCP2** volar capsular distention
21. **MCP2** volar flexor digitorum *transverse*

2 measures

1. Shoulder

Biceps tendon +

Tendon and bicipital recess

Transverse scan

Diameter

Max. diameter between tubercles

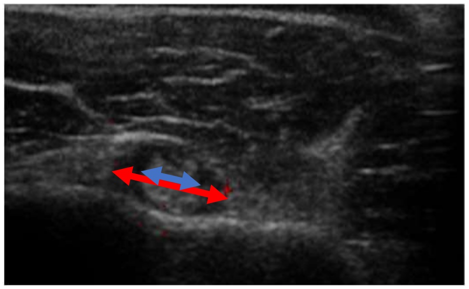

2 measures

2. Shoulder

Biceps and biceps + recess,

Distal part of recess (before tendon becomes muscle)

Transverse scan

Diameter

Max. diameter between tubercles

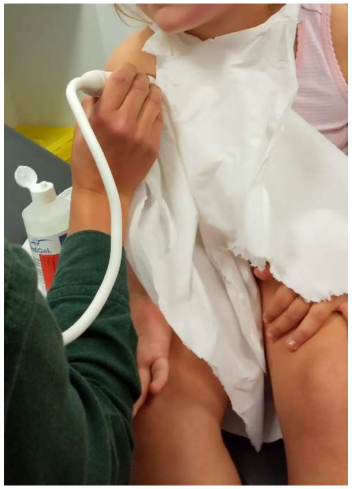

Child sitting on the egde of the table, elbow flexed 90°, palm of hand directed upwards

2 measures

12 – 15 MhZ

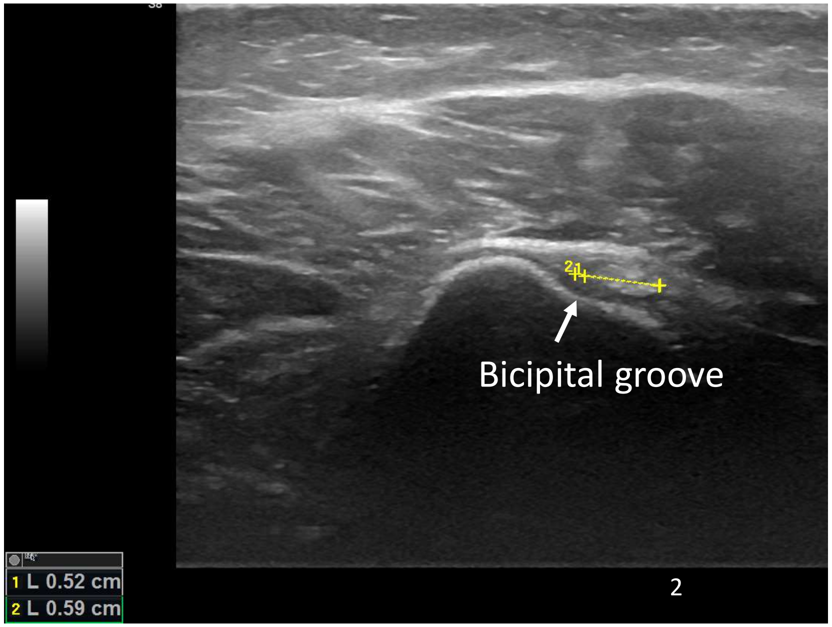

3. Hip

Acetabulofemoral recess

Capsular distention

Longitudinal sagittal

Distance to joint capsule,

from start femur neck

Max. distance

8 MhZ

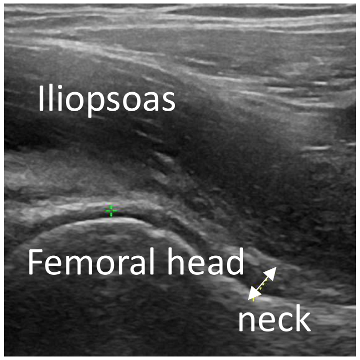

Child lying on the table,
Legs in extension, slight
external rotation of feet

4. Hip

Femoral head: cartilage thickness

Longitudinal sagittal

Thickness

Max. thickness

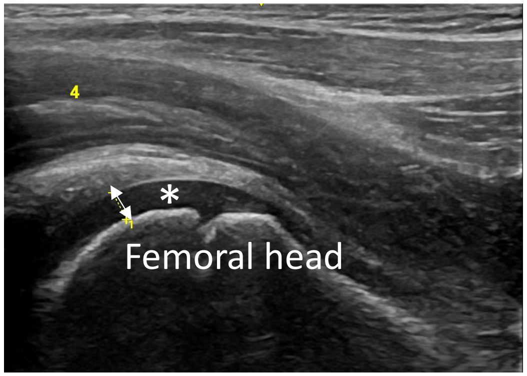

5. Knee

Distention of

Suprapatellar recess

Suprapatellar

Longitudinal sagittal

Flexion 30°

Distance from distal

Femur till recess

Max. distance

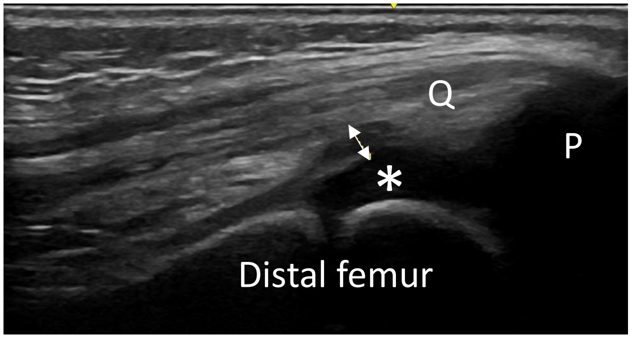

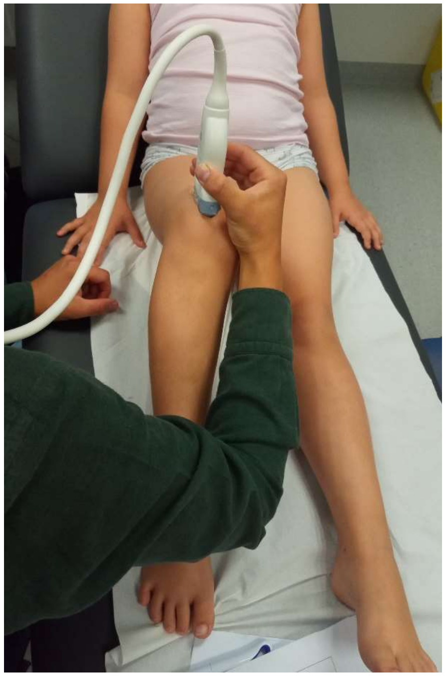

Child lying on the table (hips flexed), 30° flexion of the knee

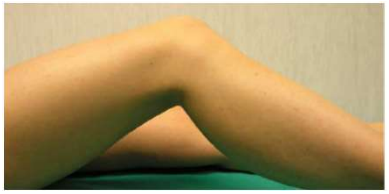

P: patella Q: quadriceps

*: cartilage

6. Knee

12 – 15 MhZ

Parapatellar recess

Suprapatellar

Transverse scan

(midpatellar)

Extension knee

Distance

Max. distance

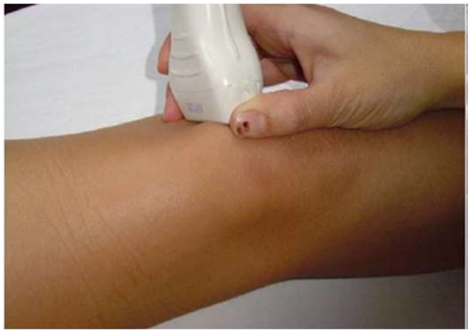

Knee in full extension

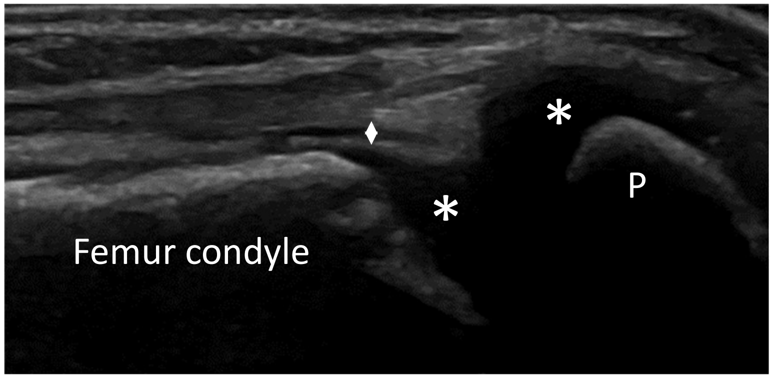

12 – 15 MhZ

7. Knee

Patellar tendon

Infrapatellar Transverse scan

Flexion 30°

Diameter

1/3 rd distal from origin

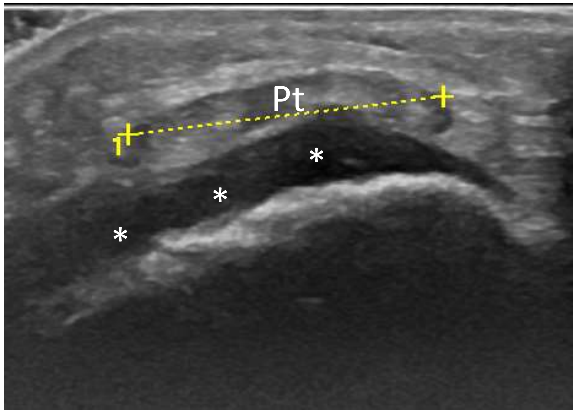

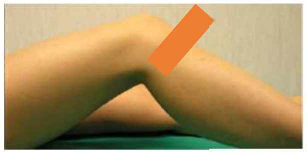

Child lying on the table (hips flexed), 30° flexion of the knee

Pt: patellar tendon

8. Knee

Maximal flexion

Cartilage trochlea (= middle, not at condyles) distal femur

Transverse scan

Thickness

Max. thickness

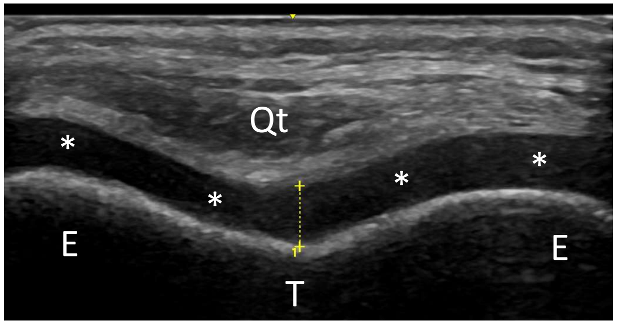

E: epicondyl
T: trochlea
*: cartilage
Qt: quadriceps tendon

Maximal flexion of the knee

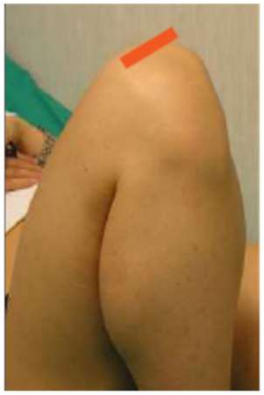

9. Ankle

Tibiotalar recess

Longitudinal

sagittal scan

Distance

Max. distance

Child lying on table,
knee 90-120° flexed,
foot flat on table

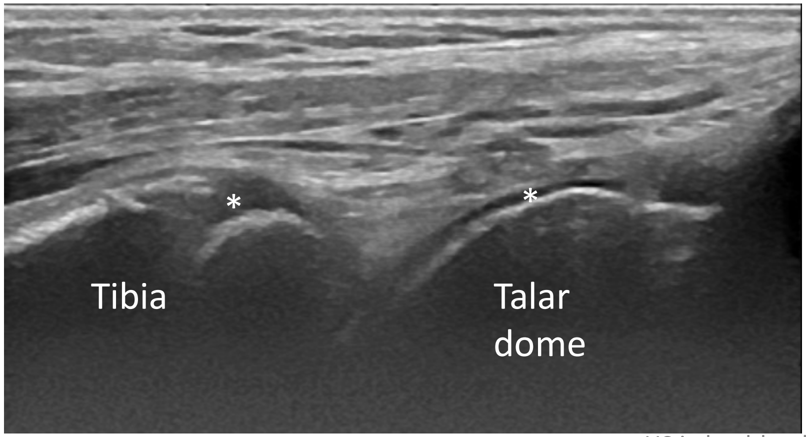

*: cartilage

10. Ankle

Cartilage thickness

talar dome

Longitudinal

sagittal scan

Thickness

Max. thickness

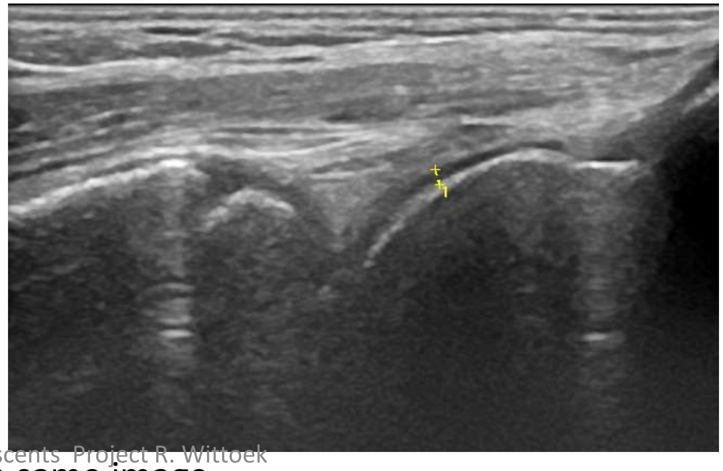

12 – 15 MhZ

9 and 10 can be assessed on same image

11. MTP1

Capsular distention of MTP1 joint

Longitudinal sagittal (midline over metatarsal 1)

max. distention

Child lying on table, knee 90-120° flexed, foot flat on table

11. MTP1

2 measures on 1 image

MTP head cartilage thickness

Longitudinal sagittal (midline over metatarsal 1)

Thickness

Max. thickness

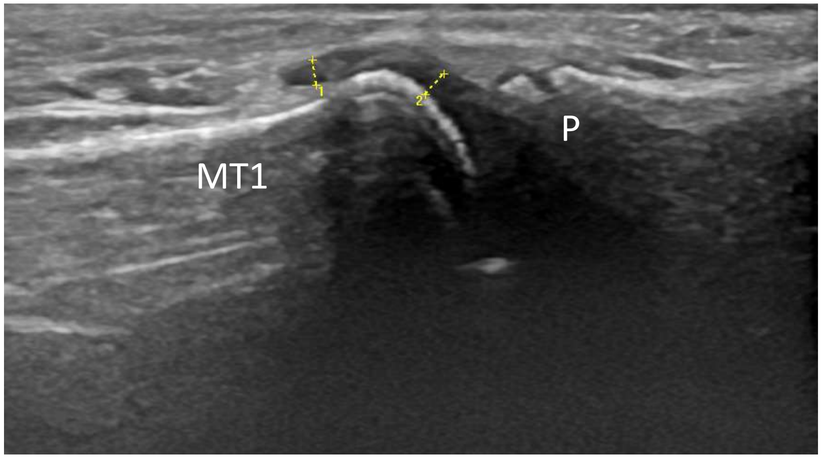

MT1: metatarsal bone 1
P: proximal phalanx

15 MhZ

12. Elbow

Lateral radiohumeral recessus

Longitudinal radial scan

Distance

Max. distance

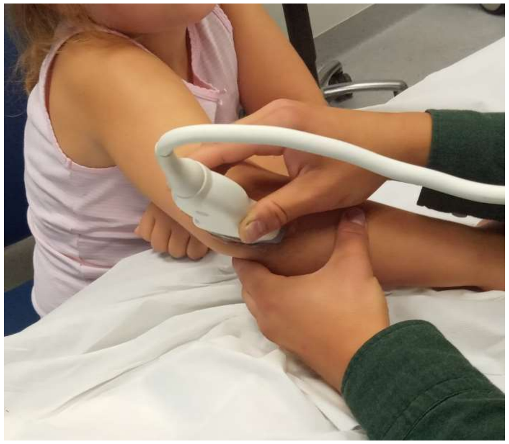

Elbow in 30° flexion, forearm on table or supported by sonographist

12 – 15 MhZ

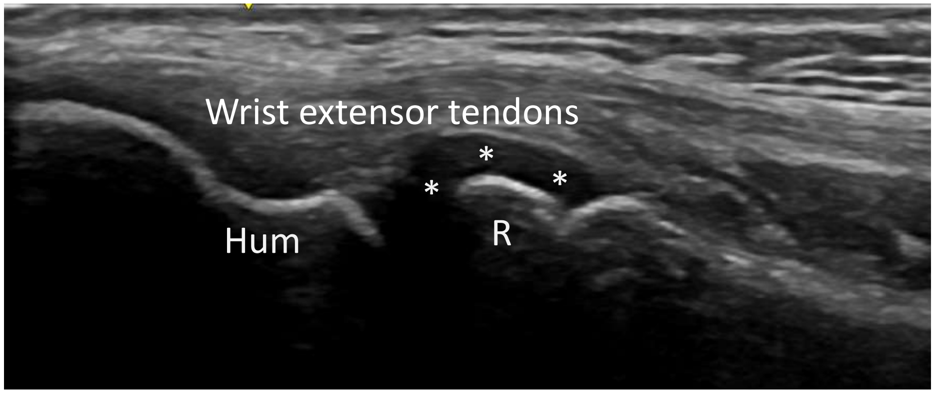

Hum: capitulum humerus R: Radial head

*: cartilage

13. Elbow

Anterior radiohumeral recessus

Longitudinal sagittal

Distance

Max. distance

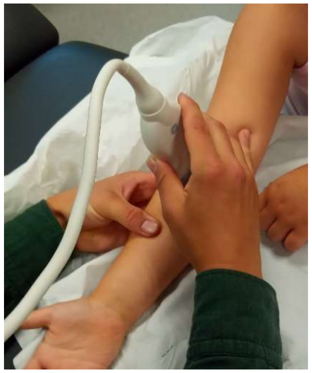

Elbow in extension (not forced)

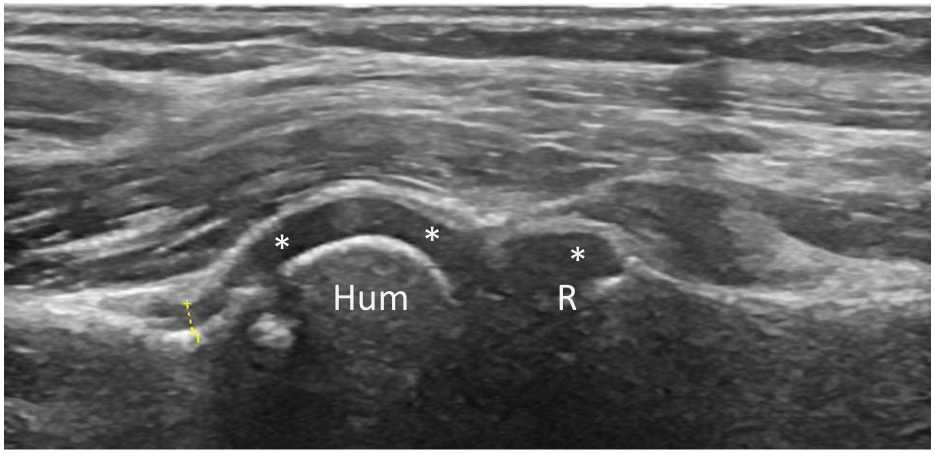

Hum: capitulum humerus R: Radial head

*: cartilage

14. Elbow

Posterior fossa/recessus

Longitudinal sagittal

Distance

Max. distance

12 – 15 MhZ

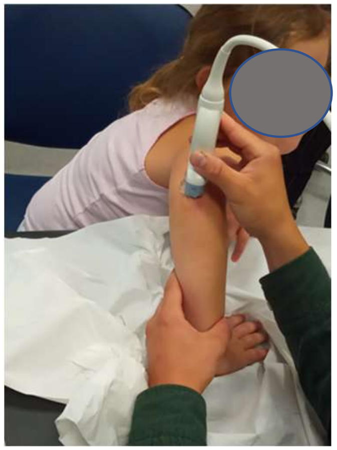

Hand flat on table, elbow flexed 90°, elbow directed towards sonographist

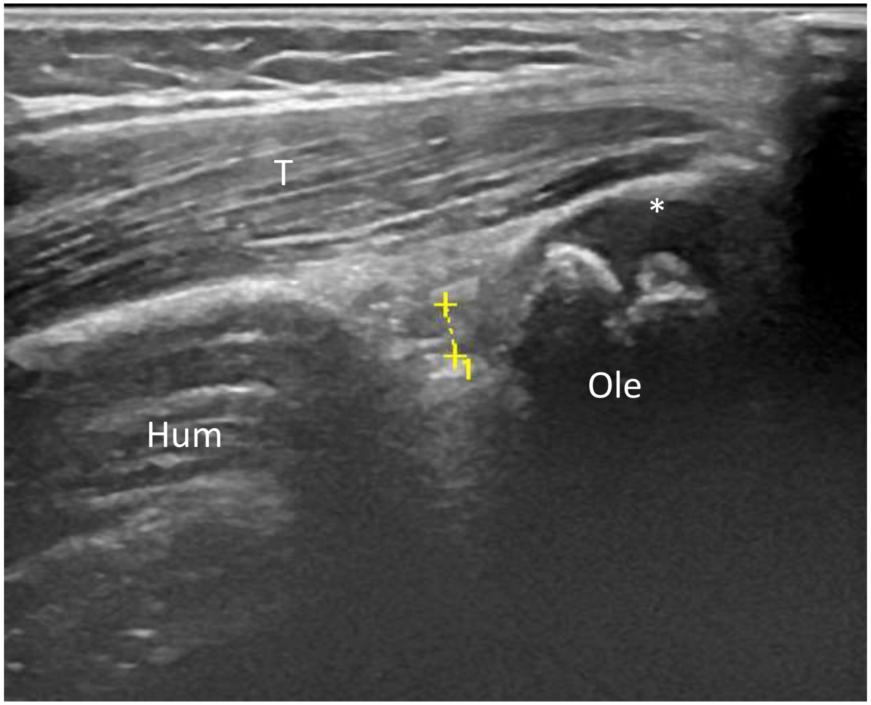

T: triceps
Hum: humerus
Ole: olecranon
*: cartilage

15. Wrist

Radiolunate – lunate capitatum – captitatum-metacarpal recess

Longitudinal midline

sagital scan

(over metacarpal 3)

Distance

Max. distance

3 measures

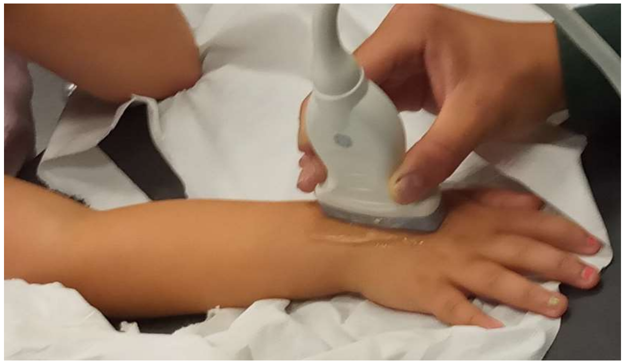

Hand and wrist flat on table, avoid radial or ulnar deviation of the hand

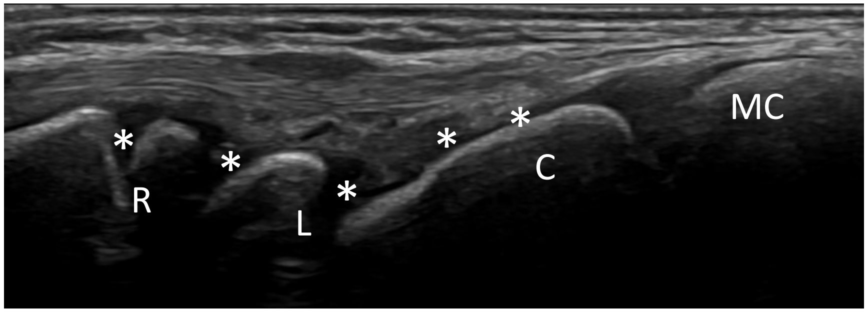

R: radius
Lun: os lunatum

Cap: os capitatum
Meta: 3rd Metacarpal bone

12 – 15 MhZ

16. Wrist

Extensor digitorum tendons

(comp IV): tendon and sheet

Transverse midcarpal

(over metacarpal 3)

Max. diameter

At Lister tubercle

2 measures

12 – 15 MhZ

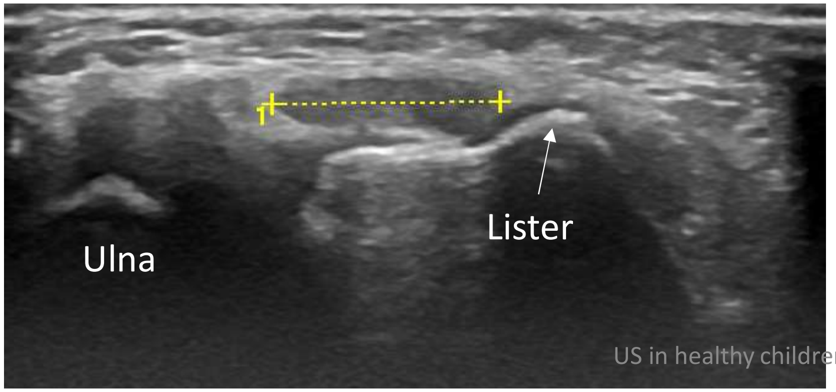

17. Wrist

Extensor carpi ulnaris tendon

(comp VI): tendon and sheet

Ulnar transverse scan

Diameter

At ulnocarpal joint

2 measures

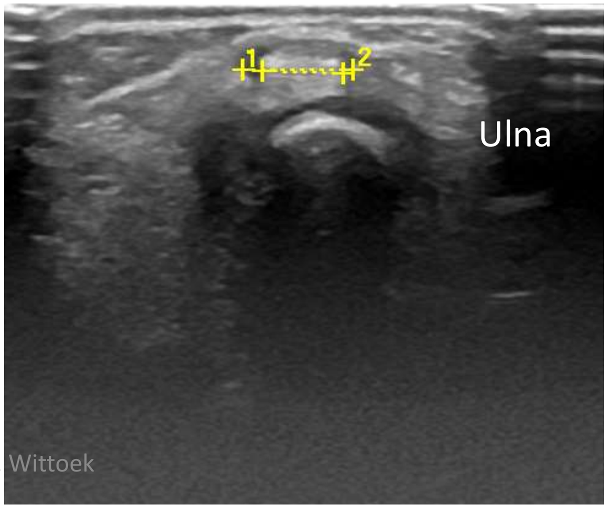

18. MCP2 dorsal

Synovial distention

Longitudinal midline

Sagittal scan

distance

Max. distance

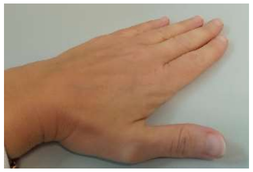

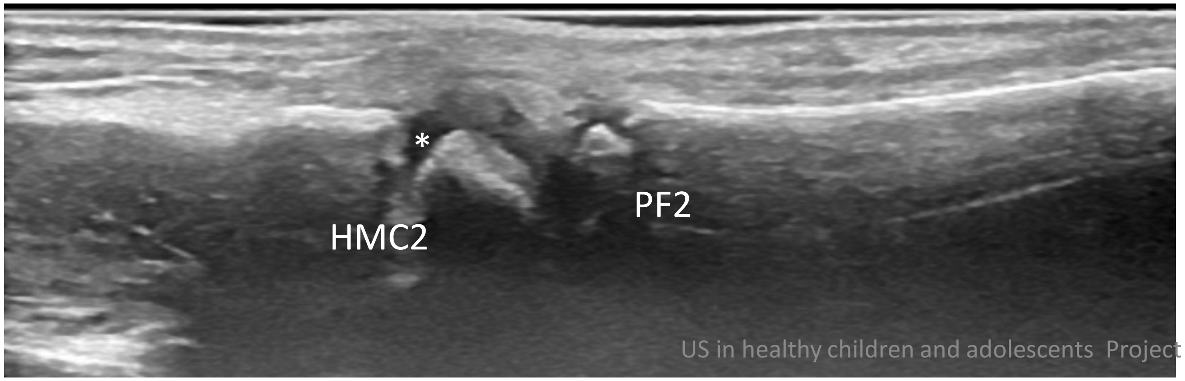

19. MCP2 dorsal

Flexion of MCP

Cartilage metacarpal head

Longitudinal midline

Sagittal scan

Thickness

Max. thickness

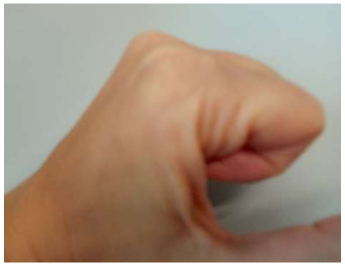

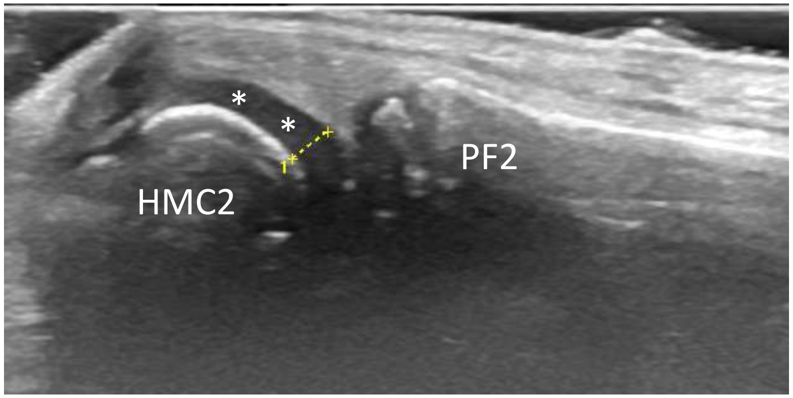

HMC2: metacarpal head 2
PF2: head of proximal phalanx 2
*: cartilage

15 MhZ

20. MCP2 volar
Capsular distention
Longitudinal midline sagittal scan
Distance
Max. diameter

20. MCP2 volar
Cartilage metacarpal head
Longitudinal midline
Sagittal scan
Thickness
Max. thickness

Dorsum hand on table
Forearm in full supination

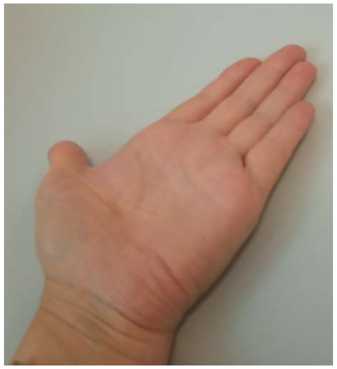

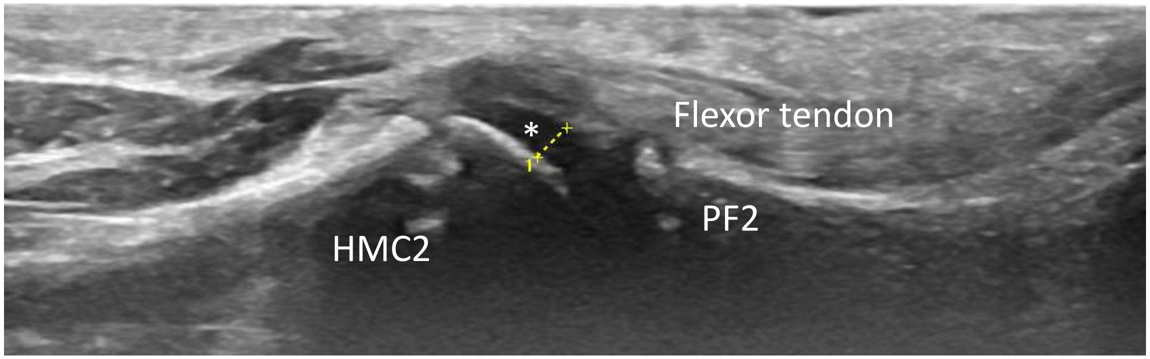

HMC2: metacarpal head 2
PF2: head of proximal phalanx 2
*: cartilage

15 MhZ

21. MCP2 volar

Flexor digitorum tendon (profundus + superficialis)
(measure just proximal to MCP2 joint)

incl. surrounding sheet

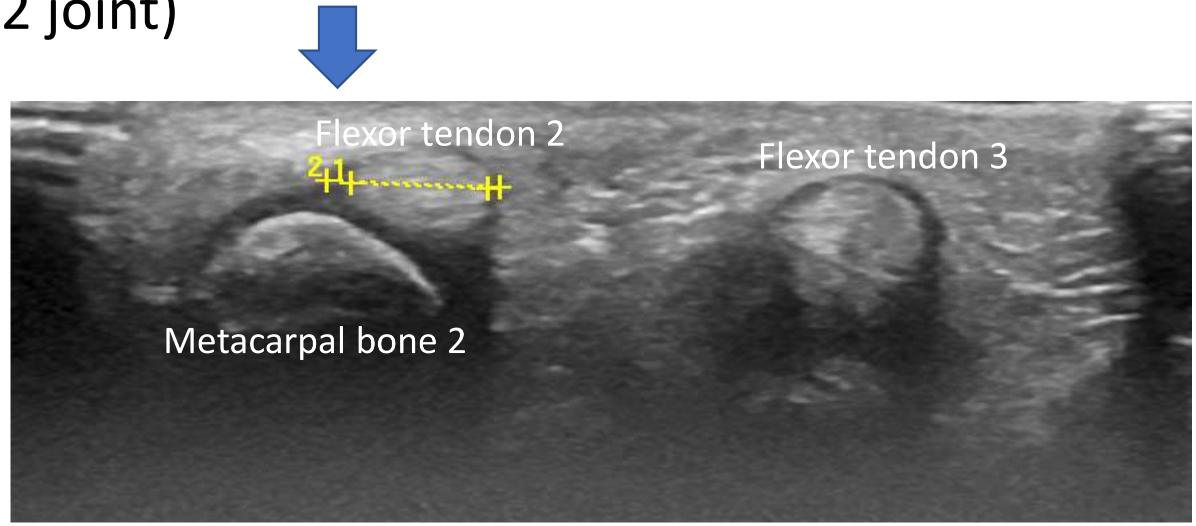

Transverse scan

Diameter

Max. diameter

2 measures

Dorsum hand on table
Forearm in full supination

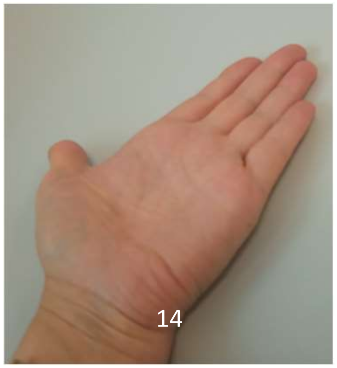

15 MhZ
