# Supplementary material for: Structural ultrasound of joints and tendons in healthy children: development of normative data
Source: Pediatr Rheumatol Online J. 2023 Sep 19;21:105. doi: 10.1186/s12969-023-00895-8 (PMC10508001; doi:10.1186/s12969-023-00895-8)
Supplement: Supplementary file 2 — Additional file 2: Supplemental data: includes supplemental table S1 (prevalence of capsular distention), supplemental table S2 – S4 (multiple regression analyses), growth tables (supplemental table S5 – S 21) and growth charts (supplemental figures S1- S26). [file 12969_2023_895_MOESM2_ESM.pdf]

Atlas of healthy joints by ultrasound in children aged 0 – 18 years

Additional material to:

STRUCTURAL ULTRASOUND OF JOINTS AND TENDONS IN HEALTHY CHILDREN: NORMATIVE DATA

Ruth Wittoek, Céline Decock, Nele Dewaele, Lara Arnold, Pieter Baeyens, Ignace De Schrijver, Lisa Pardaens, Ioannis Raftakis, Thomas Renson, Charline Rinkin, Alexander D. J. Thooft, Tine Vanhaverbeke, Caroline Verbist

Table of content

Table of content p. 2

Definition of measurements p. 5

Figure 1: Schematic view of measurements p. 6

I. Cartilage p.7

Figure 2: Decrease of cartilage at the femoral head during growth p.8

Figure 3: Decrease of cartilage at the trochlea during growth p. 10

Figure 4: Decrease of cartilage at the talar dome during growth p. 12

Figure 5: Decrease of cartilage at the head of the 1st metatarsal bone during growth p. 14

Figure 6: Decrease of cartilage at the head of the 2nd metacarpal bone during growth p. 16

Table of content

II. Recesses p. 18

Figure 7: The acetabulofemoral recess during growth p. 19

Figure 8: The suprapatellar recess during growth p. 21

Figure 9: The parapatellar recess during growth p. 23

Figure 10: The tibiotalar recess during growth p. 25

Figure 11: The recess of MTP1 joint during growth p. 27

Figure 12: The lateral radiohumeral recess during growth p. 29

Figure 13: The anterior radiohumeral recess during growth p. 31

Figure 14: The posterior fossa elbow during growth p. 33

Figure 15: Wrist: the radio-lunate recess, lunate-capitate recess, capitate-metacarpal III recess during growth p. 35

Figure 16: The Recess of MCP2 joint during growth p. 37

Table of content

III. Tendons p. 39

Figure 17: Increasing diameter of the biceps tendon during growth p. 40

Figure 18: Increasing diameter of the patellar tendon during growth p. 42

Figure 19: Increasing diameter of the extensor digitorum communis tendon during growth p. 44

Figure 20: Increasing diameter of the flexor digitorum (superficial and profound) tendon of the 2nd finger during growth p. 46

Reference list p. 48

Definition of measurements

- Normal cartilage and joint capsule were defined according to Roth et al. (1)
- Standard scans were chosen as described in the literature (2). Except for the ankle joint, the knee was in flexion (not in neutral position), and the foot placed flat on the table, as this was considered as a more comfortable and stable position for (especially younger) children.

Figure 1: Schematic view of measurements

A. The bone-capsule distance (double arrow) was measured as the maximal distance between the inner layer of the capsular layer and the underlying bone cortex
B. Enlarged view of cartilage covering the bone: cartilage thickness (double arrow) was measured as the greatest thickness of the cartilage layer, including the layer of hyaline cartilage (anechoic structure) and cartilage surface (hyperechoic line if visible), perpendicular to the bone cortex
C. Transverse view of a tendon: diameter of tendons was measured as the maximal diameter of the tendon in the transverse view, including the surrounding synovial sheet if present

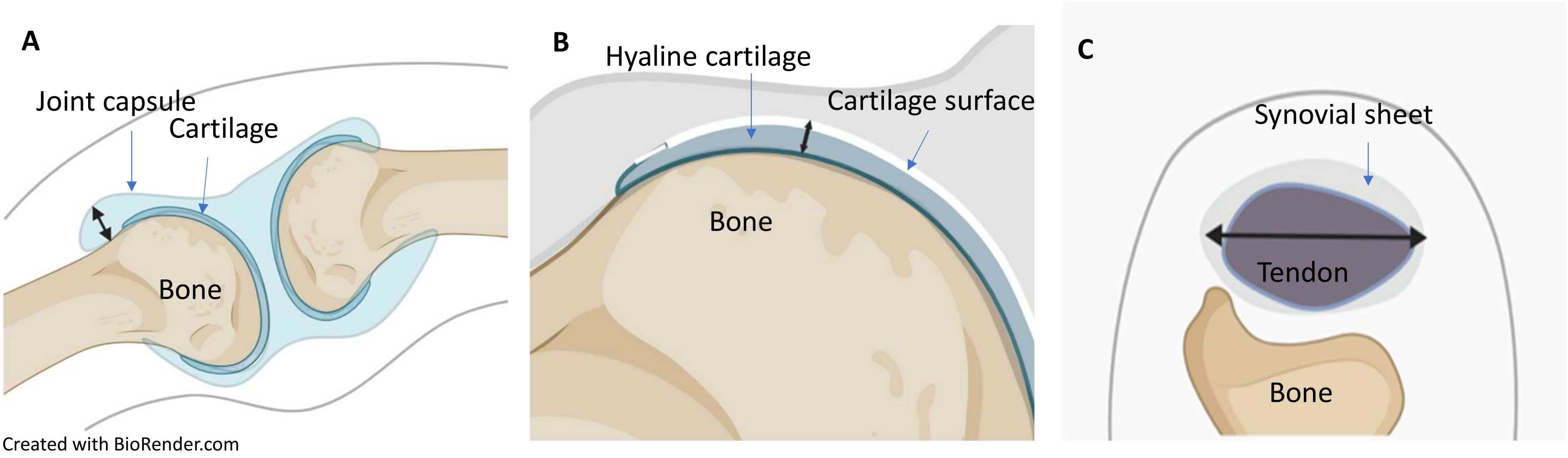

I. Cartilage

Figure 2: Decrease of cartilage at the femoral head during growth

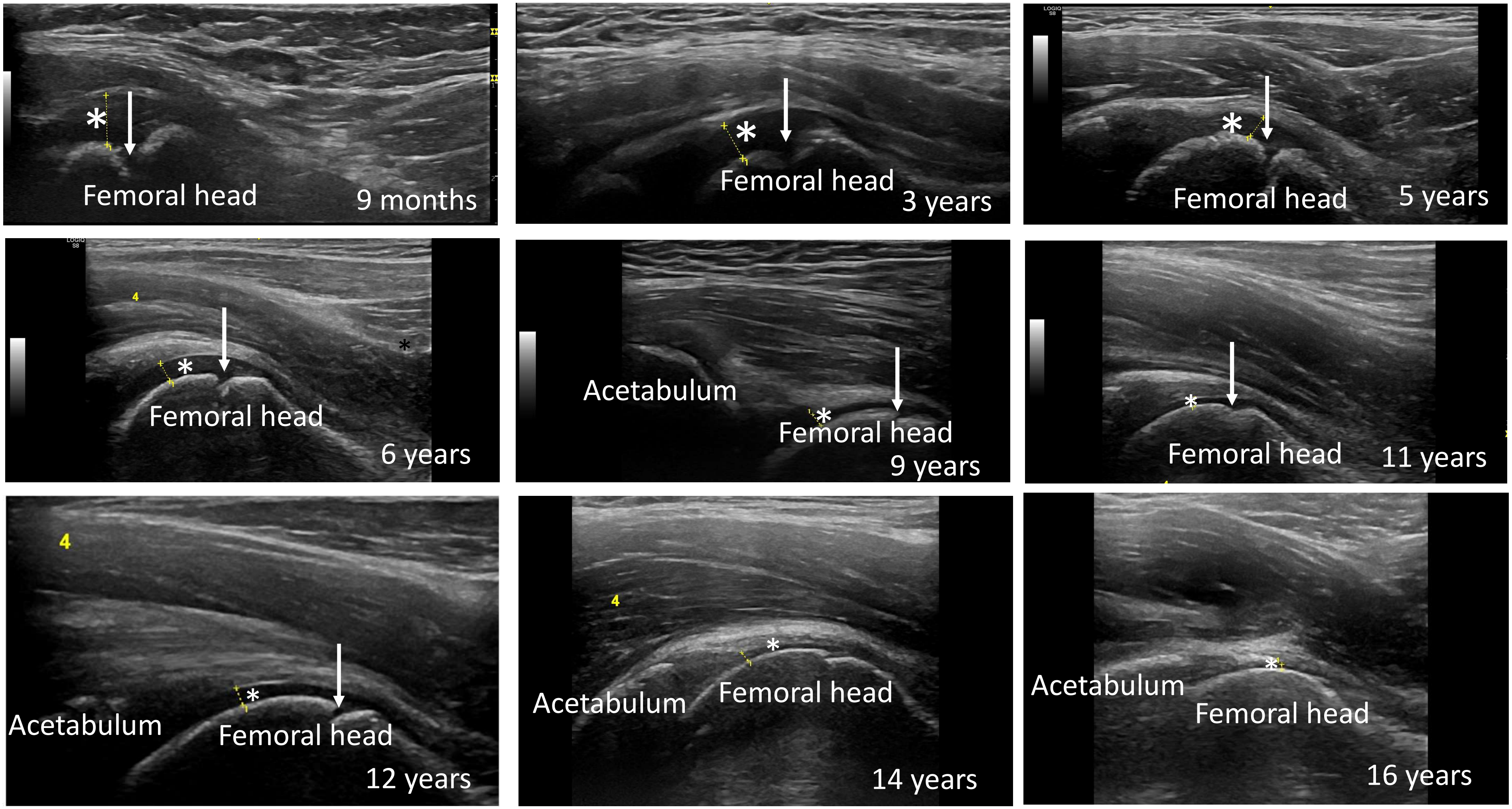

Figure 2: Decrease of cartilage at the femoral head during growth

The femoral head in anterior longitudinal view: the femoral head of the children in the younger age groups is not well rounded due to the presence of the growth plate and the incomplete ossified epiphysis. This changes around the age of 10, when the head becomes more rounded. The growth plate disappeares even a few years later.

Images shown belong to children from several age categories between 0.2 and 18 years. The specific age is explicited on the image.

Legend to images:

*: the black anechoic line represents cartilage

↓: the arrow marks the interruption of the bony cortex representing the growth plate

Figure 3: Decrease of cartilage at the trochlea during growth

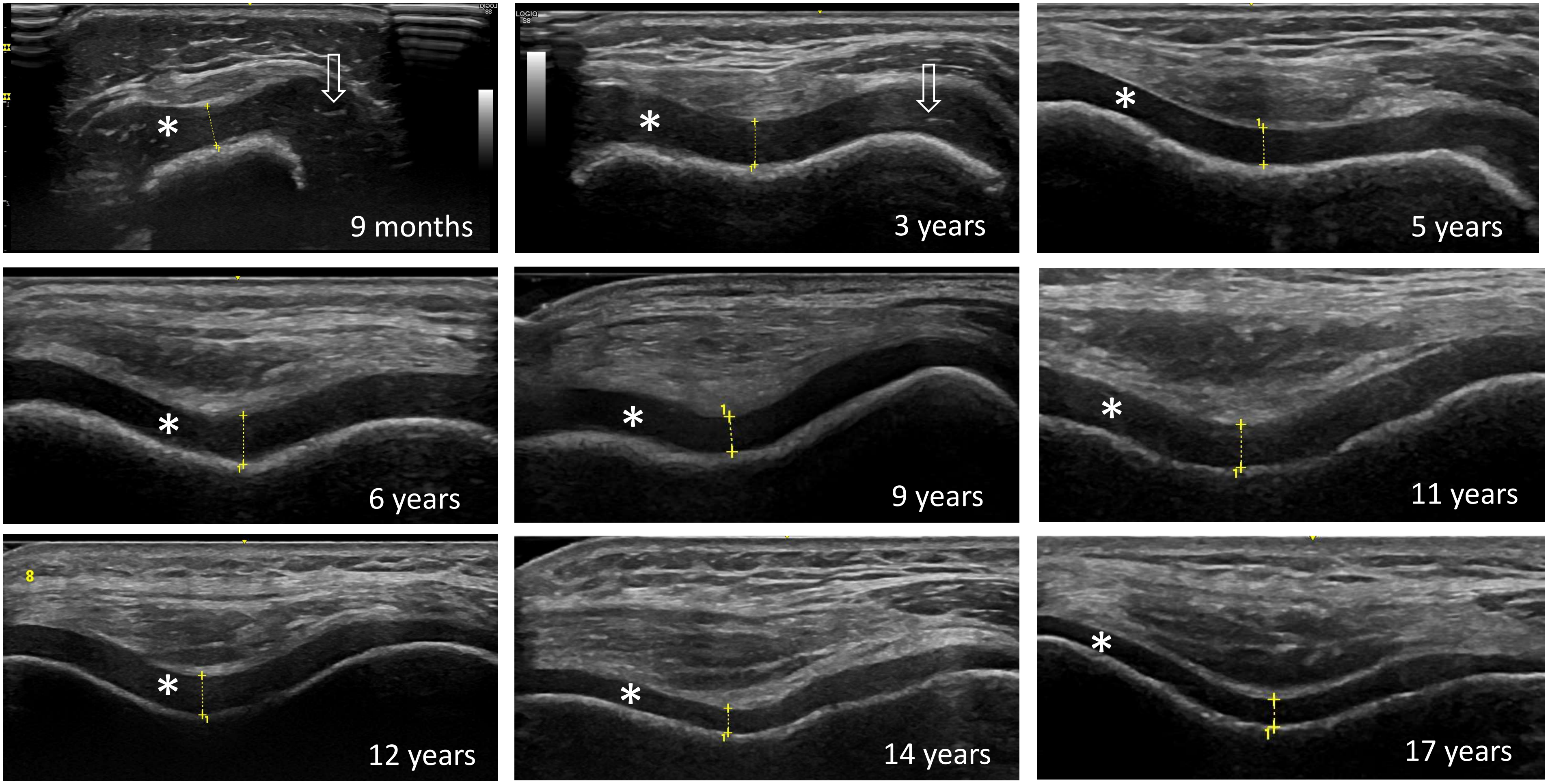

Figure 3: Decrease of cartilage at the trochlea during growth

The cartilage overlying the trochlea of the knee in maximal flexion, in transverse view: a regular, symmetric anechoic layer is visible on the bone. In the children of the younger age groups, some hyperechoic spots are visible, representing vascular channels that close when growing.

Images shown belong to children from several age categories between 0.2 and 18 years. The specific age is explicited on the image.

Legend to images:

*: the black anechoic line represents cartilage

⇩: vascular channels

Figure 4: Decrease of cartilage at the talar dome during growth

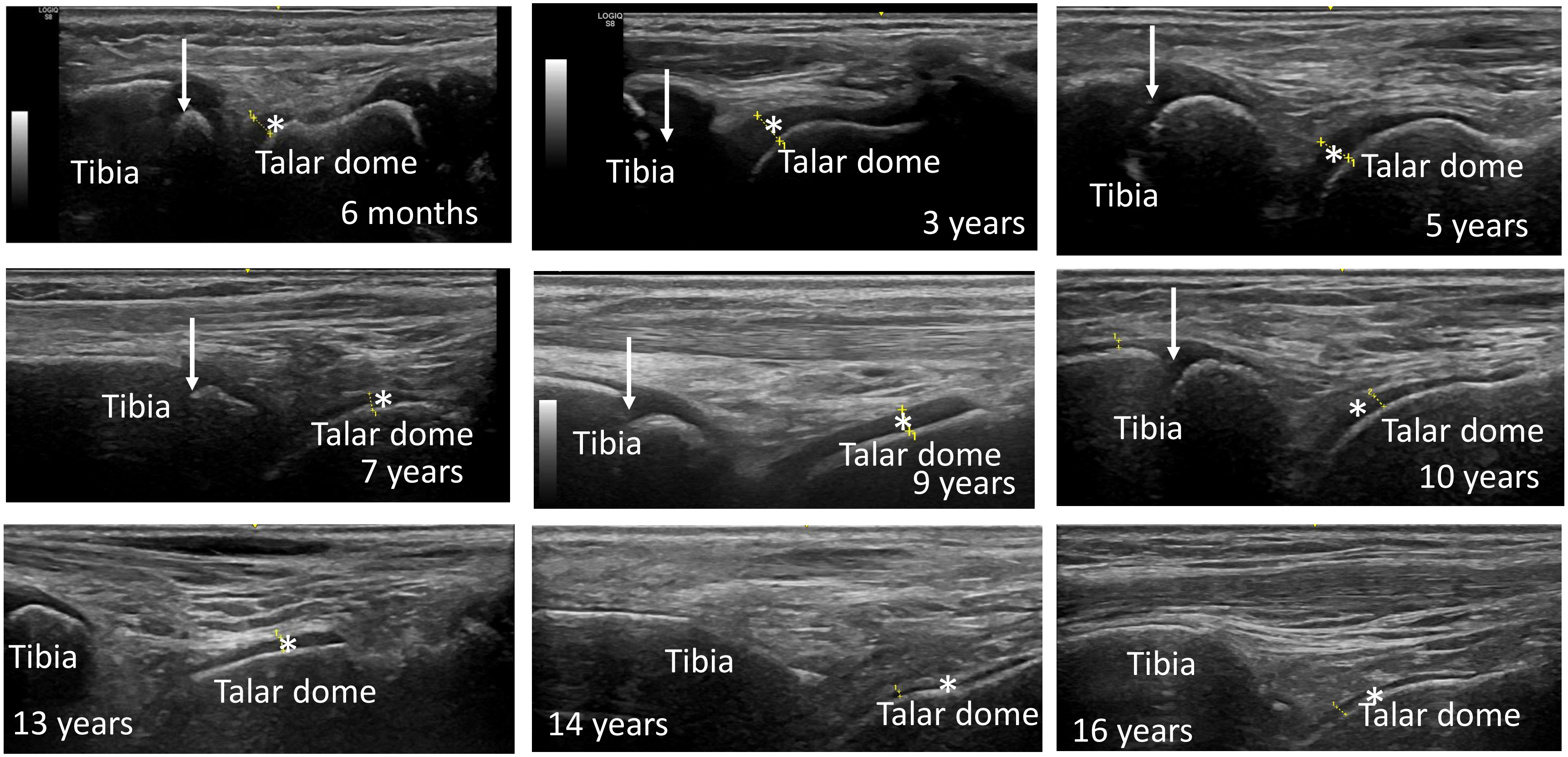

Figure 4: Decrease of cartilage at the talar dome during growth

Cartilage at the talar dome in the ankle in anterior longitudinal view: presence of growth plate and incomplete ossified epiphysis at the distal tibia in the younger children.

Images shown belong to children from several age categories between 0.2 and 18 years. The specific age is explicited on the image.

Legend to images:

*: the black anechoic line represents cartilage

↓: the arrow marks the interruption of the bony cortex representing the growth plate

Figure 5: Decrease of cartilage at the head of the 1st metatarsal bone during growth

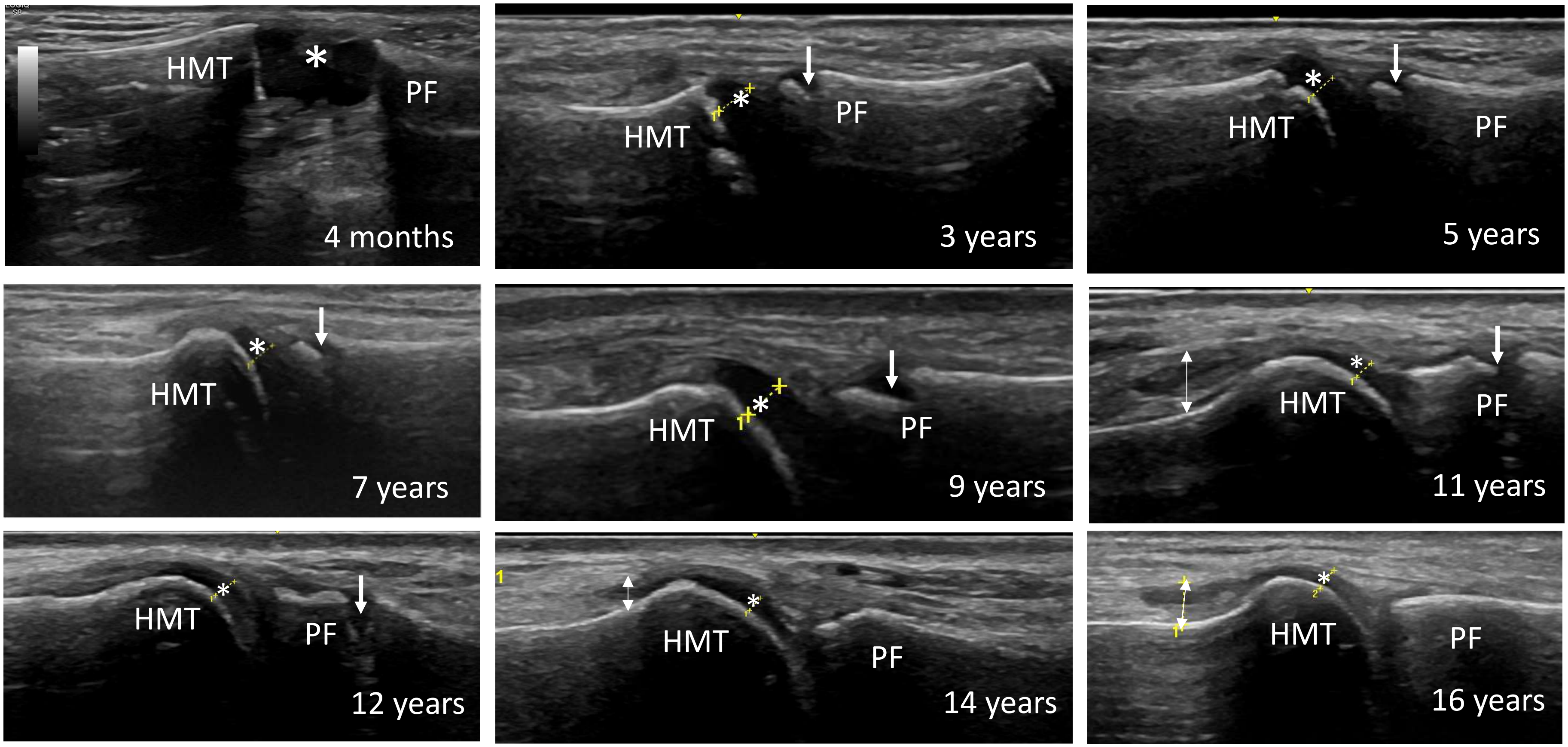

Figure 5: Decrease of cartilage at the head of the 1st metatarsal bone during growth

Cartilage at the head of the 1st metarsal bone in longitudinal midline view: presence of growth plate and incomplete ossified epiphysis at the proximal phalanx in the younger children.

Some fluid being present in the recess on the images of the children aged 11 years (middle row, right column), 14 years (lower row, middle column) and 16 years (lower row, right column).

Images shown belong to children from several age categories between 0.2 and 18 years. The specific age is explicited on the image.

Legend to images:

*: the black anechoic line represents cartilage

↓: the arrow marks the interruption of the bony cortex representing the growth plate

↕ : some capsular distention in the recess is present

HMT: head of metatarsal bone; PF: proximal phalanx

Figure 6: Decrease of cartilage at the head of the 2nd metacarpal bone during growth

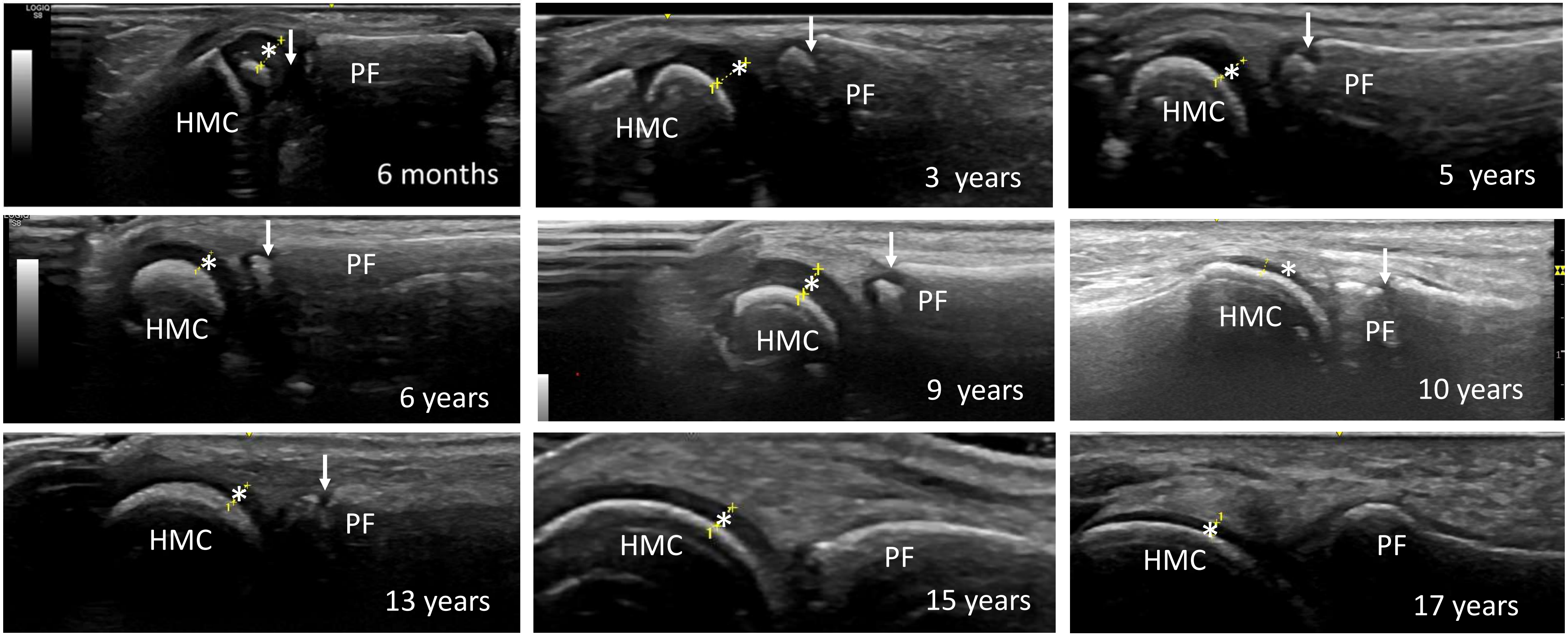

Figure 6: Decrease of cartilage at the head of the 2nd metacarpal bone during growth

The cartilage, measured at the metacarpal head of the second finger with the MCP2 joint in maximal flexion, in longitudinal midline view: presence of growth plate and incomplete ossified epiphysis at the proximal phalanx in the younger children.

Images shown belong to children from several age categories between 0.2 and 18 years. The specific age is explicited on the image.

Legend to images:

*: the black anechoic line represents cartilage

↓: the arrow marks the interruption of the bony cortex representing the growth plate

HMC: head of metacarpal bone; PF: proximal phalanx

II. Recesses

Figure 7: The acetabulofemoral recess during growth

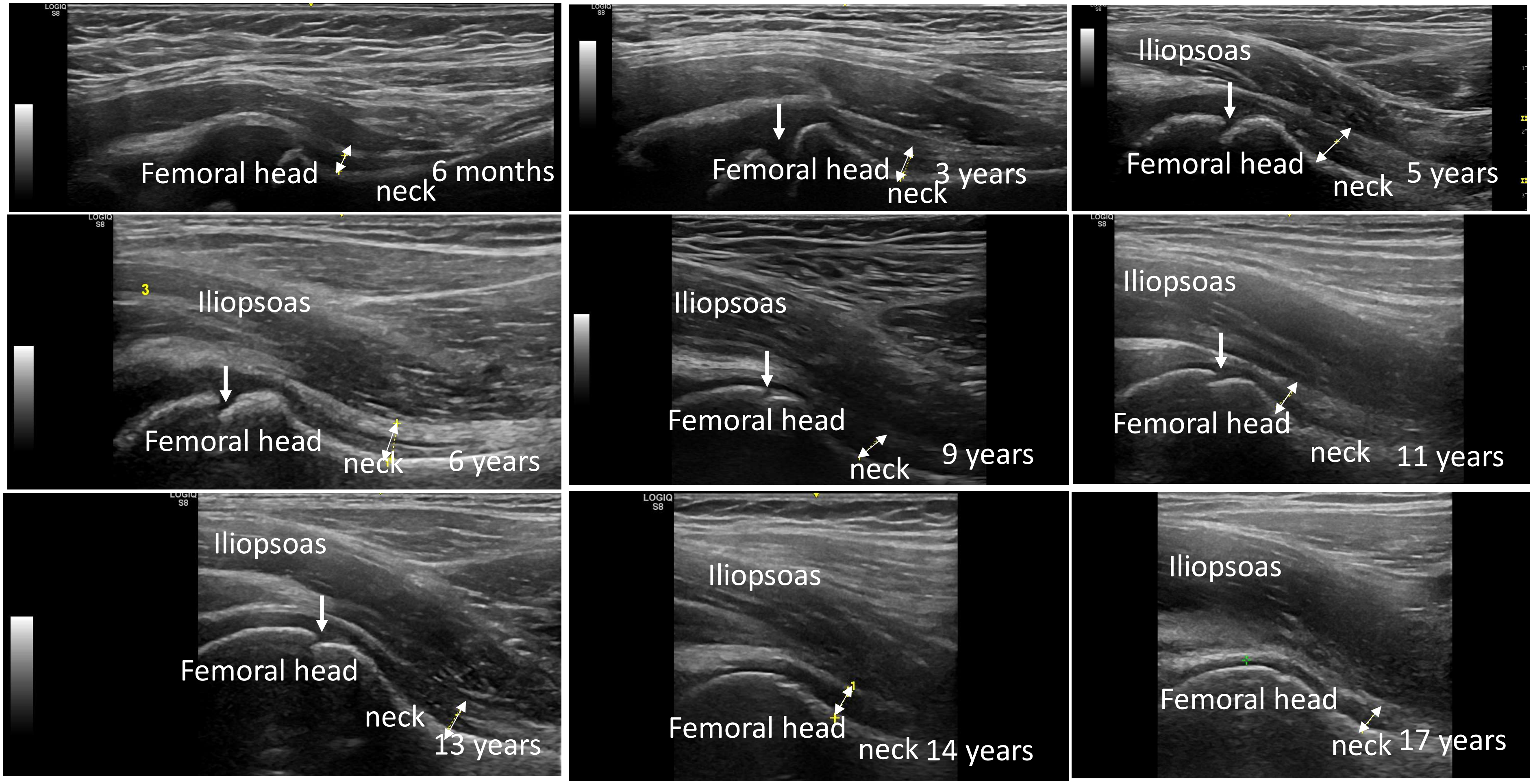

Figure 7: The acetabulofemoral recess during growth

Anterior longitudinal view: the maximal distance between the underlying bone of the femoral neck and the overlying capsule was measured (defined as the bone-capsule distance). As shown, some distention is always present but this clearly does not reflect capsular bulging what would be present in case of synovitis.

Also note to the presence of the growth plate and the incomplete ossified epiphysis at the femoral head. This closes around the age of 14 years.

Images shown belong to children from several age categories between 0.2 and 18 years. The specific age is explicited on the image.

Legend to images:

↕: bone to capsule distance

↓: the arrow marks the interruption of the bony cortex representing the growth plate

Figure 8: The suprapatellar recess during growth

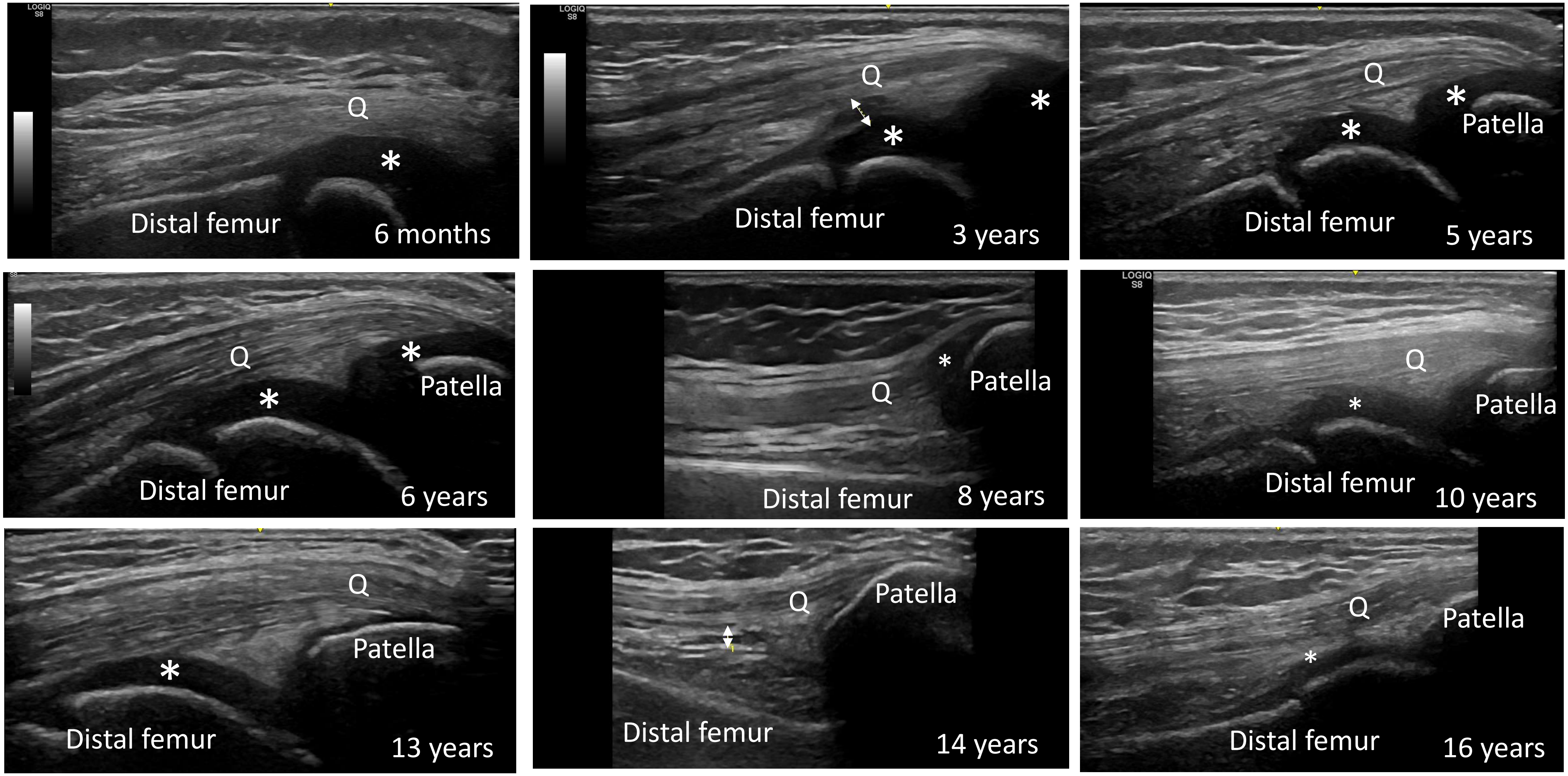

Figure 8: The suprapatellar recess during growth

Longitudinal midline sagittal view of the knee with the knee in 30° flexion: effusion in the suprapatellar recess is often seen in healthy children (here, in the images of the 3-years and 14-years old children there is some effusion present). Note the large amounts of cartilage (anechoic signal) on the distal femur, not to be misinterpreted as effusion. In the youngest children, the patella is depicted as an unossified center anterior to the distal femur.

Images shown belong to children from several age categories between 0.2 and 18 years. The specific age is explicited on the image.

Legend to images:

↕: bone to capsule distance

↓: the arrow marks the interruption of the bony cortex representing the growth plate

*: the anechoic signal represents cartilage

Q: quadriceps tendon

Figure 9: The parapatellar recess during growth

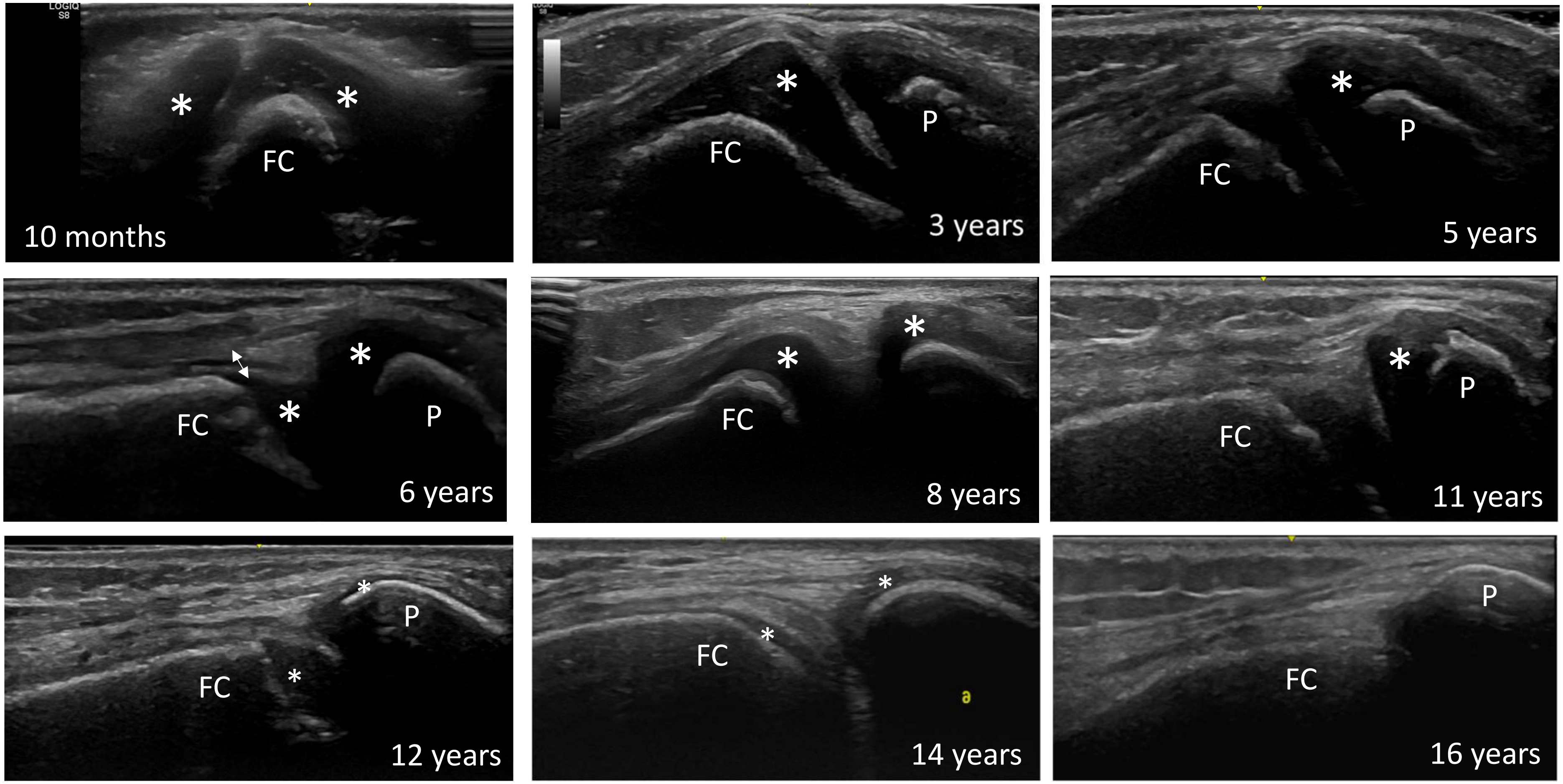

Figure 9: The parapatellar recess during growth

Transverse parapatellar (lateral) scan with the knee in maximal extension: some effusion in the parapatellar recess is seen in one quarter of healthy children. Large amounts of cartilage covers the patella and femoral condyles and decreases upon growing.

Images shown belong to children from several age categories between 0.2 and 18 years. The specific age is explicited on the image.

Legend to images:

↕: bone to capsule distance

*: the anechoic signal represents cartilage

FC: femoral lateral condyle; P: patella

Figure 10: The tibiotalar recess during growth

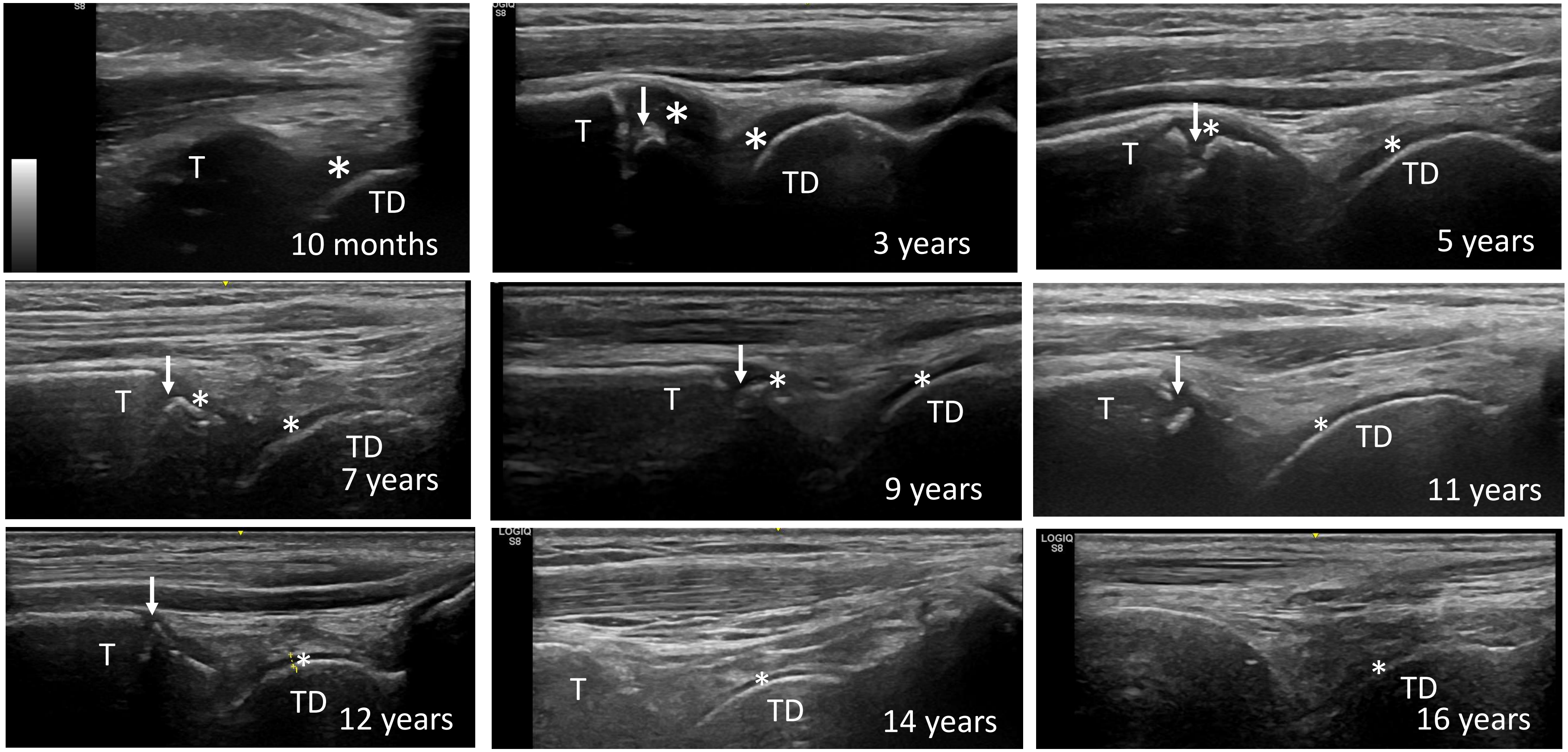

Figure 10: The tibiotalar recess during growth

Longitudinal sagittal midline view: effusion or any capsular distention of the tibiotalar recess is rare in healthy children. The cartilage on the talar dome and distal tibia is clearly decreasing upon growing.

Images shown belong to children from several age categories between 0.2 and 18 years. The specific age is explicited on the image.

Legend to images:

*: the anechoic signal represents cartilage

↓: the arrow marks the interruption of the bony cortex representing the growth plate

TD: talar dome; T: tibia

Figure 11: The recess of MTP1 joint during growth

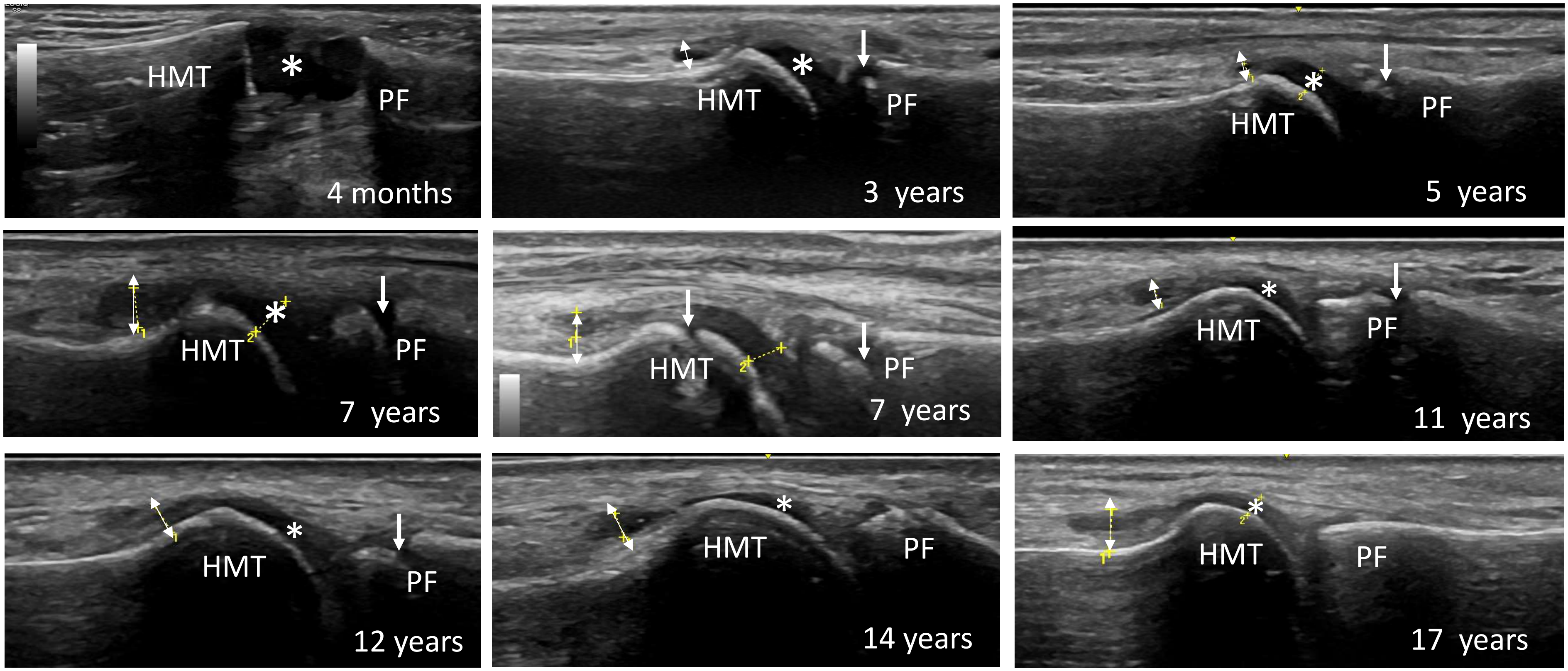

Figure 11: The recess of MTP1 joint during growth

Dorsal sagittal midline view: capsular distention (effusion and/or synovial hyperproliferation) is often seen in healthy children.

Images shown belong to children from several age categories between 0.2 and 18 years. The specific age is explicited on the image.

Legend to images:

↕: some capsular distention in the recess is present

↓: the arrow marks the interruption of the bony cortex representing the growth plate

*: the black anechoic line represents cartilage

HMT: head of metatarsal bone; PF: proximal phalanx

Figure 12: The lateral radiohumeral recess during growth

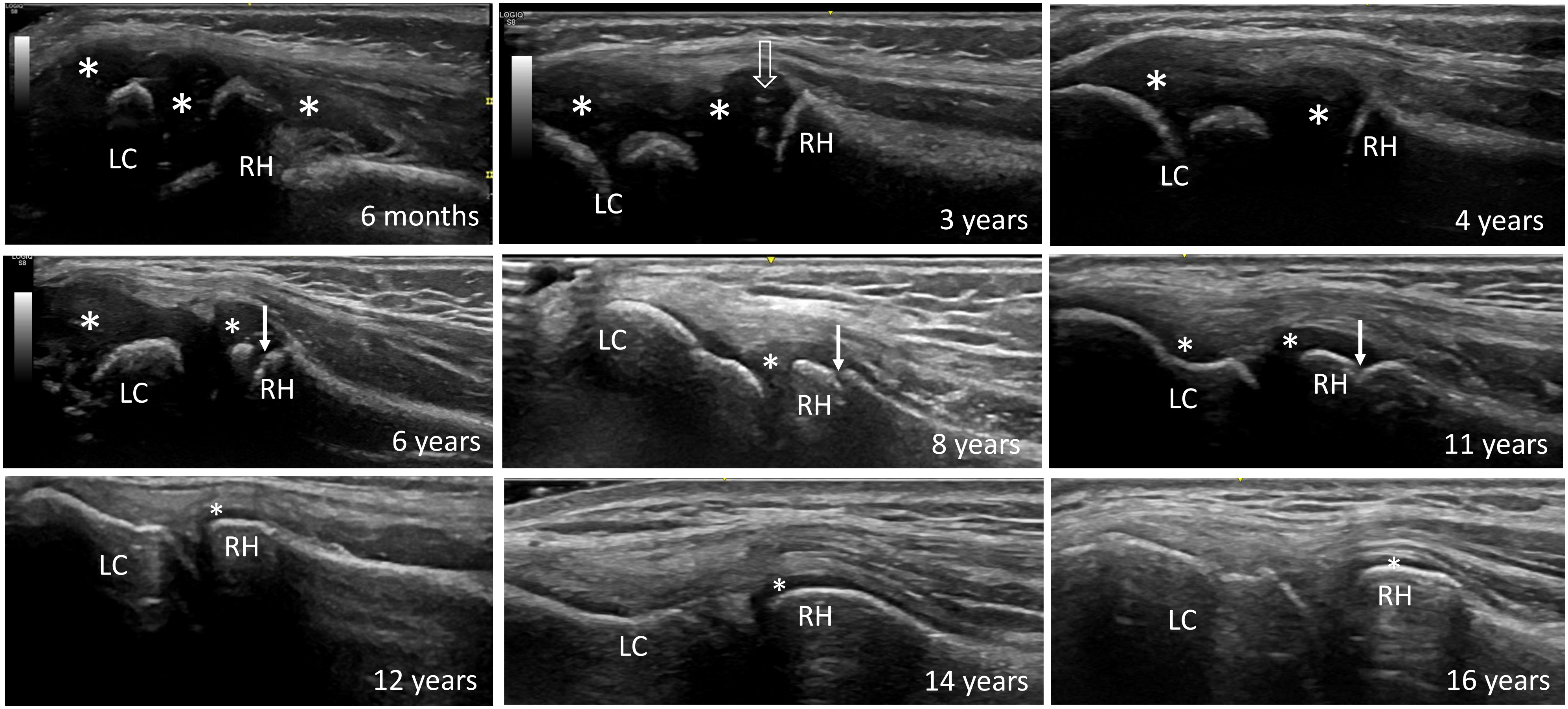

Figure 12: The lateral radiohumeral recess during growth

Longitudinal radial view over radiohumeral joint, with elbow in 30° flexion: capsular distention of the lateral radiohumeral recess is rare in healthy children. The head of the radius of the children in the younger age groups is not well rounded due to the presence of the growth plate and the incomplete ossified epiphysis. This changes around the age of 12.

Images shown belong to children from several age categories between 0.2 and 18 years. The specific age is explicited on the image.

Legend to images:

↓: the arrow marks the interruption of the bony cortex representing the growth plate

⇩: vascular channels

*: the black anechoic line represents cartilage

LC: lateral condyle of the humerus (capittelum); RH: radial head

Figure 13: The anterior radiohumeral recess during growth

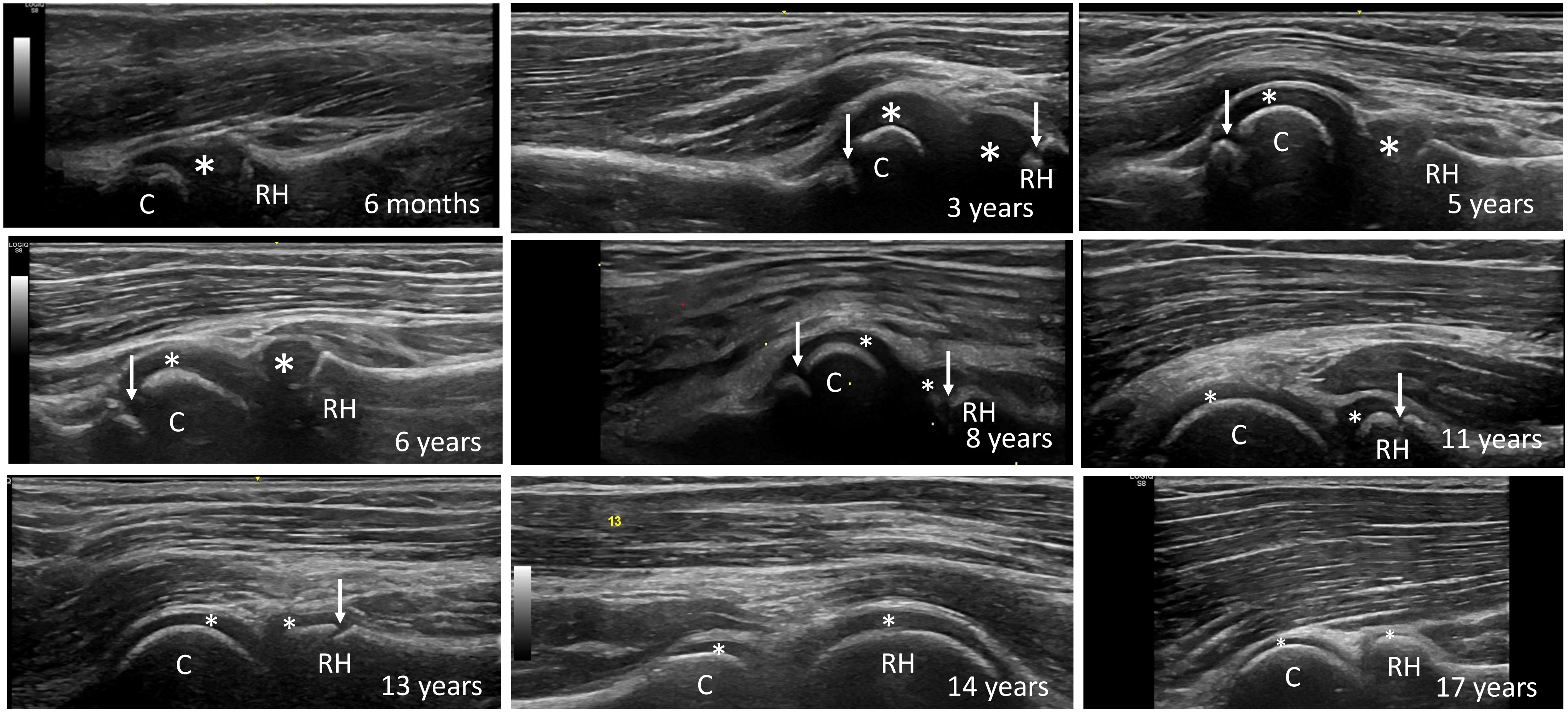

Figure 13: The anterior radiohumeral recess during growth

Anterior sagittal view with the elbow in extension (not maximal since full extension will tend to force any fluid from the joint): some distention of the anterior radiohumeral recess was seen in 10% of the healthy children. Both the capitellum as radial head develop a more rounded shape upon growing.

Images shown belong to children from several age categories between 0.2 and 18 years. The specific age is explicited on the image.

Legend to images:

↓: the arrow marks the interruption of the bony cortex representing the growth plate

*: the black anechoic line represents cartilage

C: capitellum (medial side) of the humerus; RH: radial head

Figure 14: The posterior fossa elbow during growth

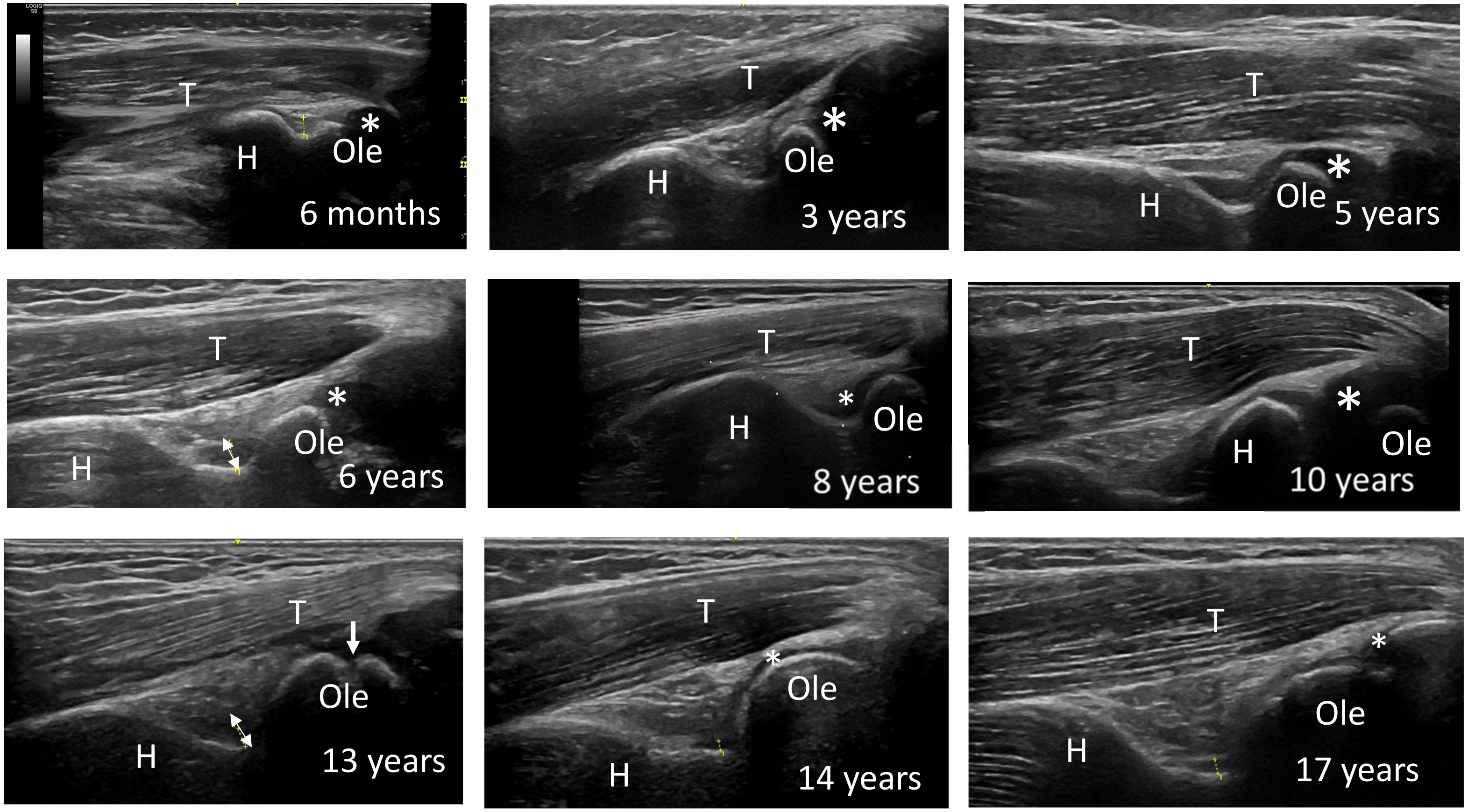

Figure 14: The posterior fossa elbow during growth

Longitudinal view with elbow in 90° flexion: some effusion in the posterior fossa is often detected in healthy children in this position.

The black signal at the insertion of the triceps tendon represents anisotrophy.

Images shown belong to children from several age categories between 0.2 and 18 years. The specific age is explicited on the image.

Legend to images:

↕: some capsular distention in the recess is present

↓: the arrow marks the interruption of the bony cortex representing the growth plate

*: the black anechoic line represents cartilage

H: humerus; Ole: olecranon; T: triceps

Figure 15: Wrist: the radio-lunate recess, lunate-capitate recess, capitate-metacarpal III recess during growth

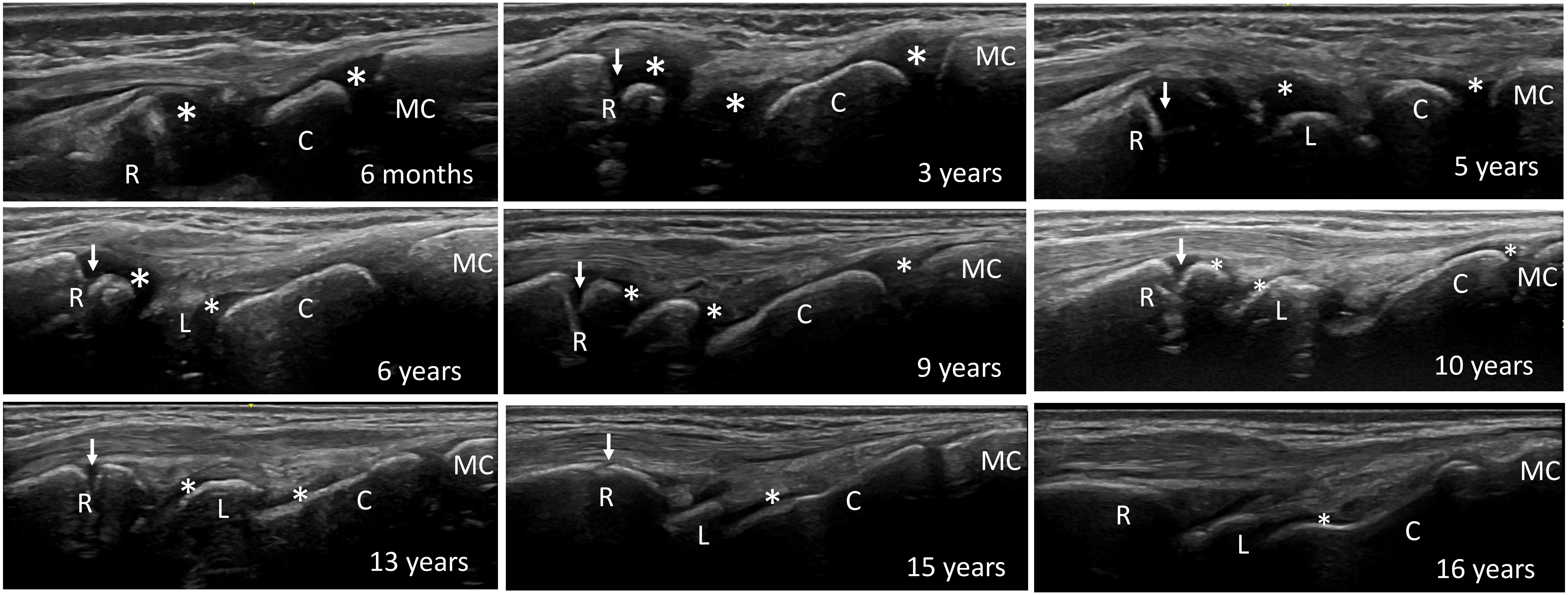

Figure 15: Wrist: the radio-lunate recess, lunate-capitate recess, capitate-metacarpal III recess during growth

Longitudinal sagittal view: effusion or capsular distention in the wrist recesses is almonst not seen in healthy children. Note that the lunate bone is not yet visible in the youngest children (due to an unossified center). Growth plate at the distal radius closes around the age of 15 years.

Images shown belong to children from several age categories between 0.2 and 18 years. The specific age is explicited on the image.

Legend to images:

↓: the arrow marks the interruption of the bony cortex representing the growth plate

*: the black anechoic line represents cartilage

R: distal radius; L: lunate bone; C: capitate bone; MC: base of the 3rd metacarpal bone

Figure 16: The Recess of MCP2 joint during growth

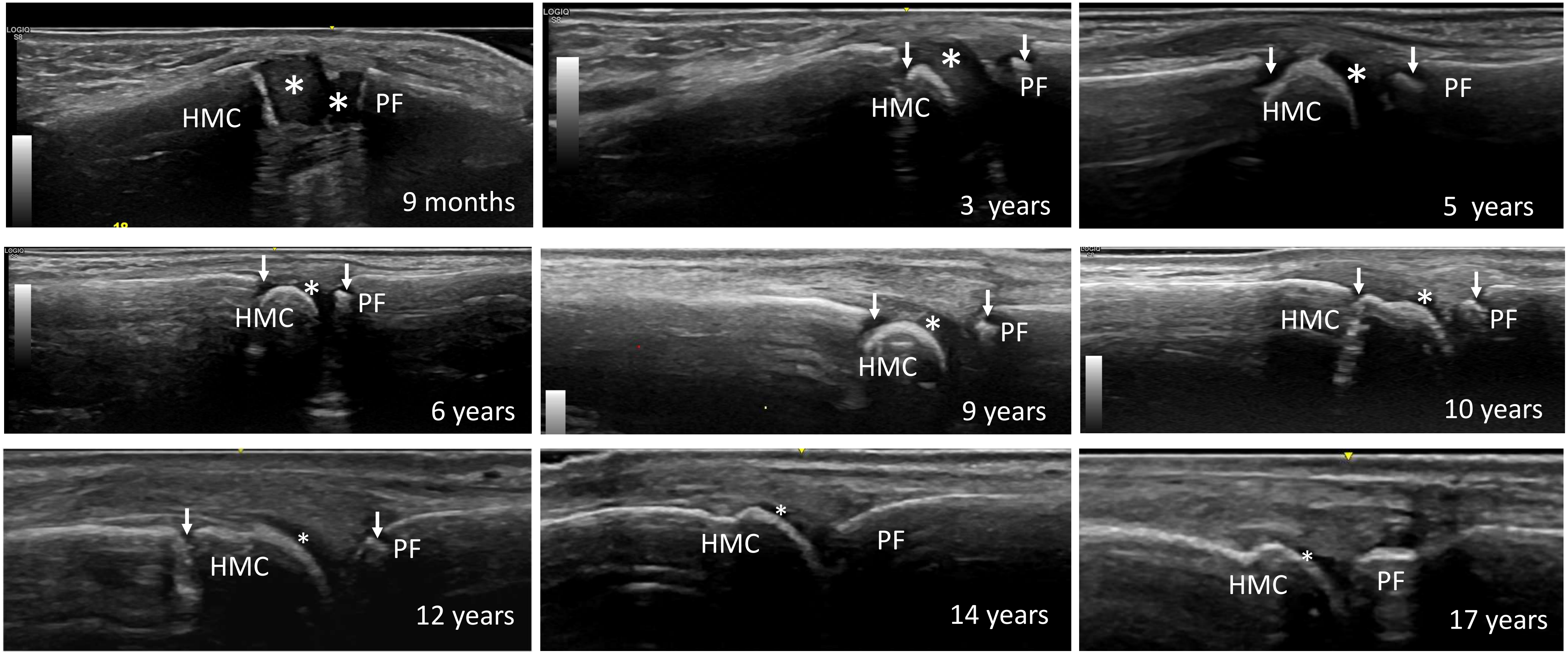

Figure 16: The Recess of MCP2 joint during growth

Longitudinal sagittal midline view (MCP joint in extension): effusion in the recess of MCP2 joint is very rare in healthy children. An abundance of cartilage is present in the MCP2 joint of little children. The cartilage is cleary decreasing while growing.

Epiphyseal growth is seen by closure of the epiphyses around the age of 14 years, both at the metacarpal head and at the base of the proximal phalanx.

Images shown belong to children from several age categories between 0.2 and 18 years. The specific age is explicited on the image.

Legend to images:

↓: the arrow marks the interruption of the bony cortex representing the growth plate

*: the black anechoic line represents cartilage

HMC: head of metacarpal bone; PF: proximal phalanx

III. Tendons

Figure 17: Increasing diameter of the biceps tendon during growth

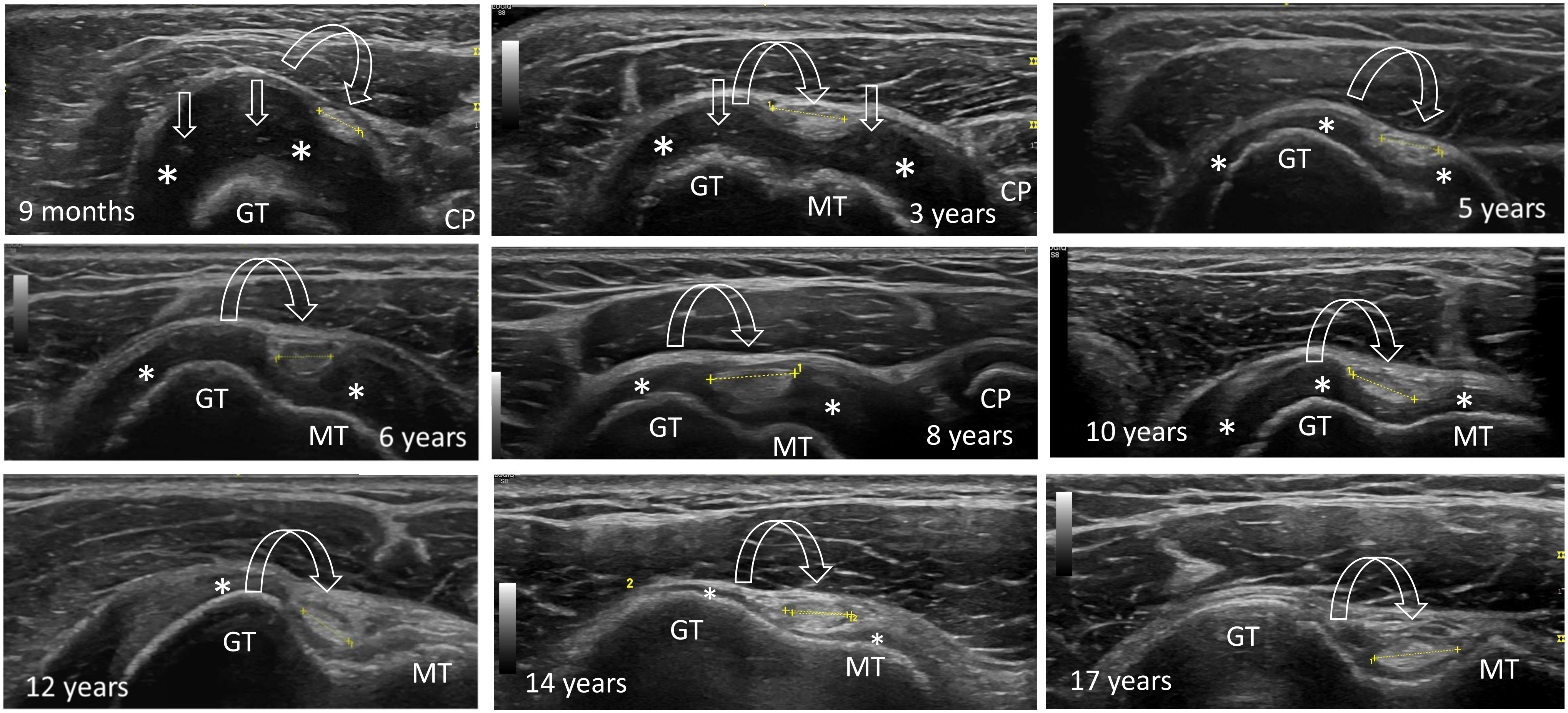

Figure 17: Increasing diameter of the biceps tendon during growth

Transverse view: the biceps tendon lying in the bicipital recess, between the lesser tuberosity and the greater tuberosity. Note the large amount of cartilage (anechoic signal with some nutrional vessels creating hyperechoic spots) covering the underlying bone on the image of the younger children. This gradually decreases as children age.

Images shown belong to children from several age categories between 0.2 and 18 years. The specific age is explicited on the image.

Legend to images:

↷: curved arrow indicates the biceps tendon (fibrillar aspect) in the bicipital groove

⇩: vascular channels

*: the black anechoic line represents cartilage

GT: greater tuberosity; MT: minor tuberosity; CP: coracoid process

Figure 18: Increasing diameter of the patellar tendon during growth

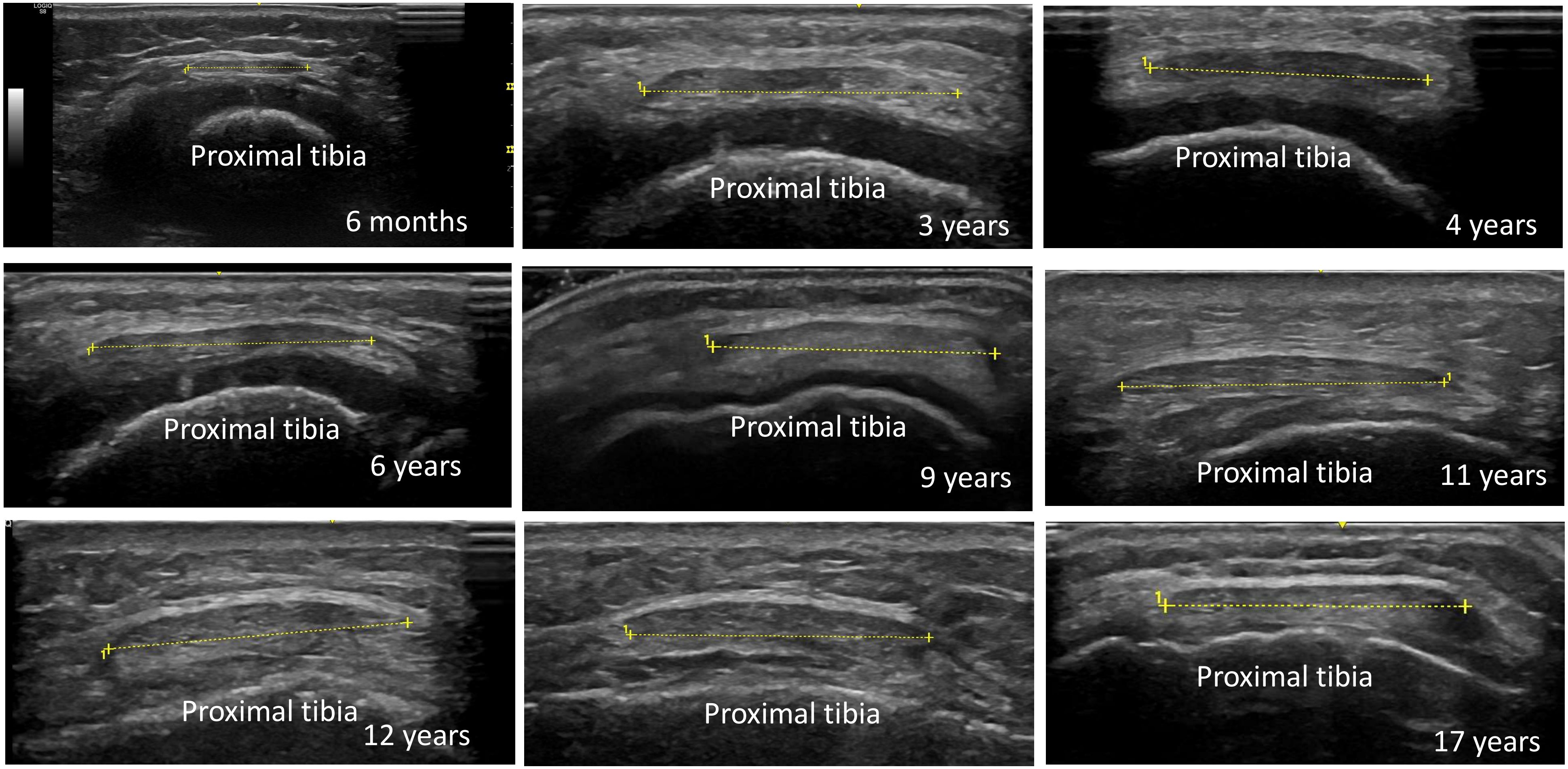

Figure 18: Increasing diameter of the patellar tendon during growth

Transverse view, probe positioned between lower border of the patella and tuberosity tibiae: maximal diameter of the patellar tendon (yellow lines).

Images shown belong to children from several age categories between 0.2 and 18 years. The specific age is explicited on the image.

Figure 19: Increasing diameter of the extensor digitorum communis tendon during growth

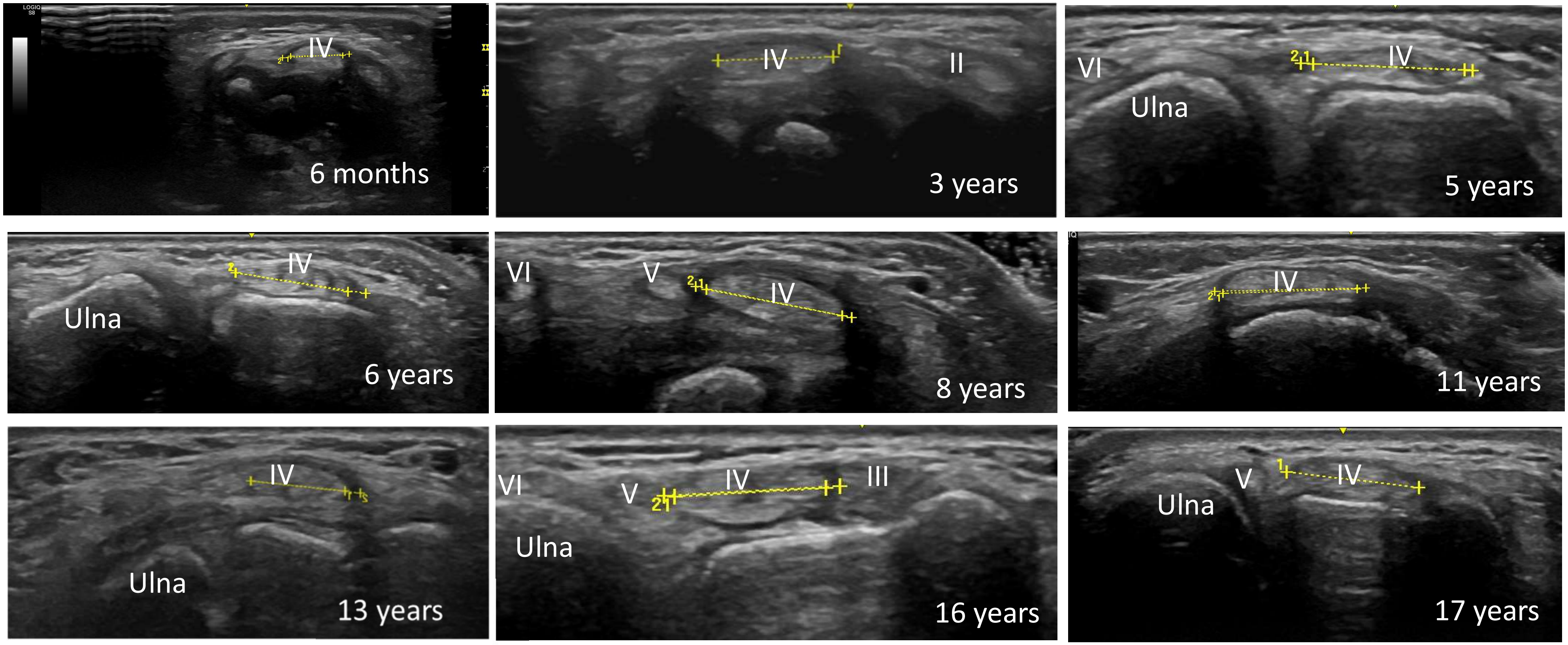

Figure 19: Increasing diameter of the extensor digitorum communis tendon during growth

Transverse view: the maximal diameter of the 4th extensor compartment is measured. A measurement was performed of the tendon alone (yellow lines, indicated as ‘1’), and another measurement including the tenovaginum (yellow lines, indicated as ‘2’).

Images shown belong to children from several age categories between 0.2 and 18 years. The specific age is explicited on the image.

Legend to image:

III to VI: extensor compartment III to VI

Figure 20: Increasing diameter of the flexor digitorum (superficial and profound) tendon of the 2nd finger during growth

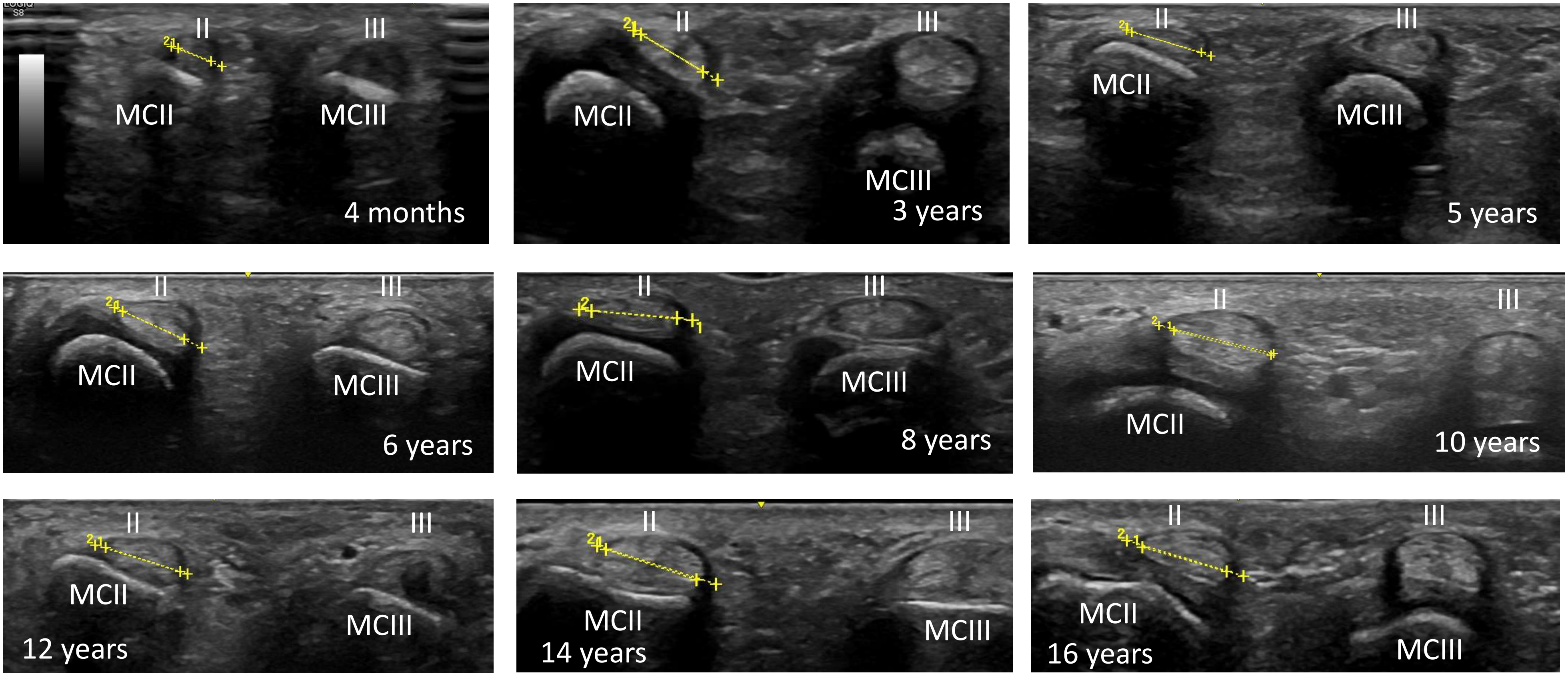

Figure 20: Increasing diameter of the flexor digitorum (superficial and profound) tendon of the 2nd finger during growth

Transverse view, volar side, proximal to MCP joint: the maximal diameter of the flexor digitorum tendon of the 2nd finger is measured. A measurement was performed of the tendon alone (yellow lines, indicated as ‘1’), and another measurement including the tenovaginum (yellow lines, indicated as ‘2’).

Images shown belong to children from several age categories between 0.2 and 18 years. The specific age is explicited on the image.

Legend to images:

II: 2nd finger; III: third finger: MC: metacarpal bone

Reference list

1. Roth J, Jousse-Joulin S, Magni-Manzoni S, Rodriguez A, Tzaribachev N, Iagnocco A, et al. Outcome Measures in Rheumatology Ultrasound Group. Definitions for the sonographic features of joints in healthy children. Arthritis Care Res (Hoboken). 2015 Jan;67(1):136-42. doi: 10.1002/acr.22410. PMID: 25047864.

2. Backhaus M, Burmester GR, Gerber T, Grassi W, Machold KP, Swen WA, et al.; Working Group for Musculoskeletal Ultrasound in the EULAR Standing Committee on International Clinical Studies including Therapeutic Trials. Guidelines for musculoskeletal ultrasound in rheumatology. Ann Rheum Dis. 2001 Jul;60(7):641-9. doi: 10.1136/ard.60.7.641. PMID: 11406516; PMCID: PMC1753749.
