# Supplementary material for: Structural ultrasound of joints and tendons in healthy children: development of normative data
Source: Pediatr Rheumatol Online J. 2023 Sep 19;21:105. doi: 10.1186/s12969-023-00895-8 (PMC10508001; doi:10.1186/s12969-023-00895-8)
Supplement: Supplementary file 3 — Additional file 3: Illustrative atlas (pdf): this file includes images of all joints and structures assessed, over the different age groups. This enables to better appreciate the changes in the growing joint. [file 12969_2023_895_MOESM3_ESM.pdf]

List of Content

Supplementary tables:

Table S1: Prevalence of capsular distention p.4

Table S2: Multiple regression analyses of cartilage thickness p.5

Table S3: Multiple regression analyses of capsular distention of synovial recesses p.6

Table S4: Multiple regression analyses of tendon diameter p.7

Table S5: Growth table: cartilage thickness at the femoral head in males p.9

Table S6: Growth table: cartilage thickness at the femoral head in females p.11

Table S7: Growth table: cartilage thickness at the trochlea of the knee in males p.13

Table S8: Growth table: cartilage thickness at the trochlea of the knee in females p.15

Table S9: Growth table: cartilage thickness at the talar dome in males p.17

Table S10: Growth table: cartilage thickness at the talar dome in females p.19

Table S11: Growth table: cartilage thickness at the head of 1st metatarsal bone in males p.21

Table S12: Growth table: cartilage thickness at the head of 1st metatarsal bone in females p.23

Table S13: Growth table: cartilage thickness at the head of 2nd metacarpal bone in males p.25

Table S14: Growth table: cartilage thickness at the head of 2nd metacarpal bone in females p.27

Table S15: Growth table: distention at the acetabulofemoral recess in males and females p.29

Table S16: Growth table: distention at the recess of MTP1 joint in males and females p.31

Table S17: Growth table: tendon diameter of the biceps tendon in males and females p.33

Table S18: Growth table: diameter of the patellar tendon in males and females p.35

Table S19: Growth table: diameter of the extensor digitorum communis tendon in males and females p.37

Table S20: Growth table: diameter of the extensor carpi ulnaris tendon in males and females p.39

Table S21: Growth table: diameter of the flexor digitorum (superficial and profound) tendon of the 2nd finger in males and females p.41

Supplementary figures:

Figure S1: Age related growth chart of cartilage thickness at the femoral head in males p.43

Figure S2: Age related growth chart of cartilage thickness at the femoral head in females p.44

Figure S3: Age related growth chart of cartilage thickness at the trochlea of the knee in males p.45

Figure S4: Age related growth chart of cartilage thickness at the trochlea of the knee in females p.46

Figure S5: Age related growth chart of cartilage thickness at the talar dome in males p.47

Figure S6: Age related growth chart of cartilage thickness at the talar dome in females p.48

Figure S7: Age related growth chart of cartilage thickness at the head of 1st metatarsal bone in males p.49

Figure S8: Age related growth chart of cartilage thickness at the head of 1st metatarsal bone in females p.50

Figure S9: Age related growth chart of cartilage thickness at the head of 2nd metacarpal bone in males p.51

Figure S10: Age related growth chart of cartilage thickness at the head of 2nd metacarpal bone in males p.52

Figure S11: Age related growth chart of bone capsule distance at the acetabulofemoral recess in males and females p.53

Figure S12: Age related growth chart of bone capsule distance at the parapatellar recess in males and females p.54

Figure S13: Age related growth chart of bone capsule distance at the tibiotalar recess in males and females p.55

Figure S14: Age related growth chart of bone capsule distance at the 1st metatarsophalangeal joint in males and females p.56

Figure S15: Age related growth chart of bone capsule distance at the lateral radiohumeral recess in males and females p.57

Figure S16: Age related growth chart of bone capsule distance at the anterior radiohumeral recess in males and females p.58

Figure S17: Age related growth chart of bone capsule distance at the posterior fossa recess of the elbow in males and females p.59

Figure S18: Age related growth chart of bone capsule distance at the radiolunate recess in males and females p.60

Figure S19: Age related growth chart of bone capsule distance at thelunate-capitate recess in males and females p.61

Figure S20: Age related growth chart of bone capsule distance at the capitate-metacarpal recess in males and females p.62

Figure S21: Age related growth chart of bone capsule distance at recess of the 2nd metacarpophalangeal joint in males and females p.63

Figure S22: Age related growth chart of diameter of the biceps tendon in males and females p.64

Figure S23: Age related growth chart of diameter of the patellar tendon in males and females p.65

Figure S24: Age related growth chart of diameter of the extensor digitorum communis tendon in males and females p.66

Figure S25: Age related growth chart of diameter of the extensor carpi ulnaris tendon in males and females p.67

Figure S26: Age related growth chart of diameter of the flexor digitorum tendon of the 2nd finger in males and females p.68

Table S1: Prevalence of capsular distention

Recess	N	BCD > 0mm (N (%))
Acetabulofemoral recess	485	485 (100%)
Suprapatellar recess	489	159 (32.5%)
Parapatellar recess	461	120 (26.0%)
Tibiotalar recess	495	8 (1.6%)
Recess of 1st MTP joint	494	223 (45.1%)
Lateral radiohumeral recess	495	2 (0.4%)
Anterior radiohumeral recess	495	52 (10.5%)
Posterior fossa elbow	493	186 (37.7%)
Radiolunate recess	474	6 (1.3%)
Lunate-capitate recess	472	3 (0.6%)
Capitate-metacarpal recess	473	2 (0.4%)
Recess of 2nd MCP joint	492	8 (1.6%)

BCD: bone-capsular distance: capsular distention was defined as bone to capsular distance > 0 mm**;** N = number; MTP = metatarsophalangeal joint; MCP = metacarpophalangeal joint

Table S2: Multiple regression analyses of cartilage thickness

	Predictors	Femoral head			Trochlea			Talar dome			Head of 1st metatarsal bone			Head of 2nd metacarpal bone dorsal side		
		Beta	p-value	Adj.R^2	Beta	p-value	Adj.R^2	Beta	p-value	Adj.R^2	Beta	p-value	Adj.R^2	Beta	p-value	Adj.R^2
Boys																
Model 1	Age (years)	-0.27	<0.0001	0.62	-0.10	<0.0001	0.43	-0.08	<0.0001	0.28	-0.15	<0.0001	0.54	-0.20	<0.0001	0.58
Model 2	Height (cm)	-0.04	<0.0001	0.65	-0.02	<0.0001	0.44	-0.01	<0.0001	0.29	-0.02	<0.0001	0.52	-0.03	<0.0001	0.62
Model 3	Weight (kg)	-0.06	<0.0001	0.519	-0.02	<0.0001	0.35	-0.02	<0.0001	0.24	-0.04	<0.0001	0.46	-0.04	<0.0001	0.45
Model 4	BMI (kg/m^2)	-0.16	<0.0001	0.09	-0.06	2e-04	0.07	-0.06	<0.0001	0.07	-0.12	<0.0001	0.14	-0.13	<0.0001	0.10
Model 5	Age (years)	-0.03	0.61	0.65	-0.05	0.28	0.44	-0.03	0.39	0.29	-0.12	<0.005	0.53	0.00	0.92	0.62
	Height (cm)	-0.05	<0.0001		-0.01	0.05		-0.02	0.19		-0.01	0.45		-0.03	<0.0001	
Model 6	Age (years)	-0.30	<0.0001	0.62	-0.12	<0.0001	0.43	-0.09	<0.0001	0.28	-0.15	<0.0001	0.53	-0.25	<0.0001	0.58
	Weight (kg)	0.01	0.22		<0.01	0.464		0.002	0.73		0.00	0.93		0.01	0.09	
Model 7	Age (years)	-0.08	0.18	0.66	-0.05	0.14	0.46	-0.04	0.29	0.28	-0.12	<0.001	0.53	-0.04	0.34	0.64
	Height (cm)	-0.04	<0.0001		-0.01	0.02		-0.01	0.14		-0.00	0.44		-0.04	<0.0001	
	Weight (kg)	0.03	<0.01		0.01	0.13		0.01	0.43		0.00	0.87		0.03	<0.0001	
Girls																
Model 1	Age (years)	-0.21	<0.0001	0.54	-0.09	<0.0001	0.43	-0.08	<0.0001	0.41	-0.12	<0.0001	0.50	-0.14	<0.0001	0.48
Model 2	Height (cm)	-0.04	<0.0001	0.59	-0.02	<0.0001	0.43	-0.01	<0.0001	0.41	-0.02	<0.0001	0.51	-0.03	<0.0001	0.55
Model 3	Weight (kg)	-0.05	<0.0001	0.43	-0.02	<0.0001	0.36	-0.02	<0.0001	0.32	-0.03	<0.0001	0.39	-0.03	<0.0001	0.36
Model 4	BMI (kg/m^2)	-0.14	<0.0001	0.10	-0.08	<0.0001	0.13	-0.06	<0.0001	0.11	-0.09	<0.0001	0.13	-0.08	<0.0001	0.08
Model 5	Age (years)	0.03	0.45	0.59	-0.05	0.03	0.43	-0.03	0.14	0.42	-0.05	0.07	0.51	0.08	0.01	0.56
	Height (cm)	-0.04	<0.0001		-0.01	0.08		-0.01	0.02		-0.01	0.01		-0.04	<0.0001	
Model 6	Age (years)	-0.25	<0.0001	0.55	-0.09	<0.0001	0.43	-0.09	<0.0001	0.41	-0.15	<0.0001	0.50	-0.18	<0.0001	0.49
	Weight (kg)	0.01	0.09		0.00	0.93		0.00	0.25		0.01	0.12		0.01	0.02	
Model 7	Age (years)	-0.03	0.55	0.60	-0.06	0.030	0.43	-0.05	0.05	0.42	-0.07	0.01	0.52	0.02	0.45	0.59
	Height (cm)	-0.05	<0.0001		-0.01	0.07		-0.01	0.01		-0.02	<0.005		-0.04	<0.0001	
	Weight (kg)	0.03	<0.005		0.00	0.66		0.01	0.10		0.01	0.03		0.02	<0.0001	

Adj.R^2: adjusted R squared: adjusted for the number of predictors in the model

Table S3: Multiple regression analyses of distention of synovial recesses

	Predictors	Recess of 1st MTP joint			Acetabulofemoral recess		
		Beta	p-value	Adj.R^2	Beta	p-value	Adj.R^2
Boys							
Model 1	Age (years)	0.08	<0.0001	0.14	0.09	<0.0001	0.09
Model 2	Height (cm)	0.01	<0.0001	0.13	0.02	<0.0001	0.11
Model 3	Weight (kg)	0.02	<0.0001	0.09	0.02	<0.0001	0.09
Model 4	BMI (kg/m²)	0.04	0.05	0.02	0.06	0.06	0.01
Model 5	Age (years)	0.06	0.31	0.13	-0.07	0.38	0.11
	Height (cm)	0.00	0.71		0.03	0.04	
Model 6	Age (years)	0.13	7e-04	0.14	0.06	0.27	0.09
	Weight (kg)	-0.01	0.15		0.01	0.58	
Model 7	Age (years)	0.09	0.14	0.14	-0.07	0.42	0.10
	Height (cm)	0.01	0.37		0.03	0.05	
	Weight (kg)	-0.02	0.10		0.00	0.93	
Girls							
Model 1	Age (years)	0.05	<0.0001	0.06	0.13	<0.0001	0.18
Model 2	Height (cm)	0.01	<0.0001	0.06	0.02	<0.0001	0.21
Model 3	Weight (kg)	0.01	<0.0001	0.04	0.04	<0.0001	0.20
Model 4	BMI (kg/m²)	0.03	0.09	0.006	0.13	<0.0001	0.07
Model 5	Age (years)	0.05	0.27	0.06	-0.07	0.29	0.21
	Height (cm)	0.00	0.94		0.03	<0.0001	
Model 6	Age (years)	0.08	<0.01	0.06	0.04	0.38	0.20
	Weight (kg)	-0.01	0.26		0.03	0.02	
Model 7	Age (years)	0.07	0.14	0.06	-0.11	0.10	0.22
	Height (cm)	0.00	0.77		0.03	<0.005	
	Weight (kg)	-0.01	0.25		0.02	0.08	

Adj.R^2: adjusted R squared: adjusted for the number of predictors in the model; BMI: body mass index (kg/m²)

Table S4: Multiple regression analyses of tendon diameter

	Predictors	Patellar tendon			Biceps tendon			Extensor digitorum communis tendon			Extensor carpi ulnaris tendon			Flexor digitorum tendon* of the 2nd finger		
		Beta	p-value	Adj.R^2	Beta	p-value	Adj.R^2	Beta	p-value	Adj.R^2	Beta	p-value	Adj.R^2	Beta	p-value	Adj.R^2
Boys																
Model 1	Age (years)	0.87	<0.0001	0.56	0.15	<0.0001	0.24	0.39	<0.0001	0.41	0.25	<0.0001	0.47	0.19	<0.0001	0.43
Model 2	Height (cm)	0.14	<0.0001	0.56	0.03	<0.0001	0.30	0.06	<0.0001	0.45	0.04	<0.0001	0.52	0.03	<0.0001	0.48
Model 3	Weight (kg)	0.19	<0.0001	0.43	0.03	<0.0001	0.20	0.10	<0.0001	0.39	0.06	<0.0001	0.43	0.04	<0.0001	0.38
Model 4	BMI (kg/m²)	0.50	<0.0001	0.08	0.08	0.02	0.02	0.33	<0.0001	0.12	0.19	<0.0001	0.12	0.15	<0.0001	0.12
Model 5	Age (years)	-0.02	0.93	0.59	-0.20	<0.01	0.33	-0.05	0.69	0.45	-0.05	0.54	0.52	-0.05	0.44	0.48
	Height (cm)	0.14	<0.0001		0.06	<0.0001		0.07	<0.0001		0.05	<0.0001		0.04	<0.0001	
Model 6	Age (years)	1.12	<0.0001	0.56	0.18	<0.0001	0.24	0.28	<0.005	0.41	0.21	<0.0001	0.47	0.15	<0.0001	0.43
	Weight (kg)	-0.07	0.07		-0.01	0.62		0.03	0.18		0.01	0.39		0.01	0.35	
Model 7	Age (years)	0.21	0.33	0.62	-0.15	0.04	0.34	-0.06	0.66	0.44	-0.03	0.68	0.52	-0.04	0.55	0.47
	Height (cm)	0.18	<0.0001		0.07	<0.0001		0.07	<0.005		0.05	<0.0001		0.04	0.001	
	Weight (kg)	-0.13	<0.0001		-0.03	0.02		0.00	0.86		-0.01	0.55		-0.01	0.60	
Girls																
Model 1	Age (years)	0.71	<0.0001	0.42	0.11	<0.0001	0.15	0.25	<0.0001	0.23	0.18	<0.0001	0.29	0.16	<0.0001	0.38
Model 2	Height (cm)	0.13	<0.0001	0.51	0.02	<0.0001	0.18	0.05	<0.0001	0.28	0.03	<0.0001	0.31	0.03	<0.0001	0.45
Model 3	Weight (kg)	0.18	<0.0001	0.36	0.03	<0.0001	0.12	0.06	<0.0001	0.18	0.04	<0.0001	0.25	0.04	<0.0001	0.29
Model 4	BMI (kg/m²)	0.50	<0.0001	0.09	0.08	0.00	0.03	0.12	<0.01	0.02	0.14	<0.0001	0.09	0.10	<0.0001	0.06
Model 5	Age (years)	-0.67	<0.0001	0.53	-0.09	0.12	0.19	-0.22	0.02	0.29	0.00	0.99	0.31	-0.10	0.02	0.46
	Height (cm)	0.24	<0.0001		0.03	0.001		0.08	<0.0001		0.03	0.005		0.05	<0.0001	
Model 6	Age (years)	0.70	<0.0001	0.42	0.12	0.02	0.15	0.32	<0.0001	0.24	0.16	<0.0001	0.29	0.21	<0.0001	0.39
	Weight (kg)	-0.01	0.89		0.00	0.73		-0.02	0.2		0.01	0.67		-0.01	0.09	
Model 7	Age (years)	-0.54	<0.005	0.54	-0.06	0.31	0.19	-0.13	0.22	0.30	0.01	0.94	0.31	-0.04	0.35	0.47
	Height (cm)	0.25	<0.0001		0.04	0.001		0.09	<0.0001		0.03	<0.005		0.05	<0.0001	
	Weight (kg)	-0.05	0.07		-0.01	0.29		-0.04	0.02		0.00	0.83		-0.02	<0.005	

*: including the superficial and profound tendons; Ext.: extensor; Adj.R^2: adjusted R squared: adjusted for the number of predictors in the model; BMI: body mass index (kg/m^2)

Table S5: Growth table: cartilage thickness at the femoral head in males

Year	5% percentile	50% percentile	95% percentile
2.0	3.17	4.69	7.78
2.5	3.02	4.45	7.09
3.0	2.93	4.23	6.51
3.5	2.86	4.02	6.05
4.0	2.81	3.83	5.71
4.5	2.75	3.65	5.48
5.0	2.69	3.50	5.33
5.5	2.62	3.36	5.25
6.0	2.55	3.24	5.18
6.5	2.47	3.13	5.11
7.0	2.38	3.03	5.04
7.5	2.28	2.94	4.95
8.0	2.18	2.85	4.86
8.5	2.07	2.75	4.76
9.0	1.96	2.65	4.65
9.5	1.84	2.54	4.53
10.0	1.72	2.42	4.40
10.5	1.60	2.30	4.27
11.0	1.47	2.17	4.12
11.5	1.36	2.04	3.97
12.0	1.25	1.93	3.81
12.5	1.15	1.82	3.64
13.0	1.07	1.72	3.46
13.5	0.99	1.63	3.27
14.0	0.93	1.55	3.08
14.5	0.88	1.48	2.88
15.0	0.84	1.43	2.67

15.5	0.81	1.38	2.48
16.0	0.79	1.35	2.30
16.5	0.78	1.32	2.15
17.0	0.79	1.30	2.02

Values are in millimeter

Table S6: Growth table: cartilage thickness at the femoral head in females

Year	5% percentile	50% percentile	95% percentile
2.0	2.34	4.27	6.85
2.5	2.22	3.90	6.53
3.0	2.19	3.6	6.22
3.5	2.21	3.37	5.91
4.0	2.25	3.19	5.61
4.5	2.27	3.06	5.31
5.0	2.27	2.97	5.03
5.5	2.24	2.91	4.75
6.0	2.18	2.84	4.47
6.5	2.09	2.77	4.21
7.0	1.99	2.69	3.95
7.5	1.87	2.60	3.73
8.0	1.74	2.51	3.54
8.5	1.62	2.42	3.40
9.0	1.51	2.32	3.32
9.5	1.40	2.22	3.28
10.0	1.30	2.12	3.29
10.5	1.20	2.02	3.36
11.0	1.11	1.92	3.45
11.5	1.02	1.82	3.55
12.0	0.94	1.72	3.61
12.5	0.87	1.61	3.62
13.0	0.80	1.51	3.57
13.5	0.75	1.42	3.44
14.0	0.71	1.34	3.26
14.5	0.68	1.27	3.01
15.0	0.68	1.23	2.73

15.5	0.70	1.20	2.45
16.0	0.73	1.20	2.23
16.5	0.78	1.22	2.10
17.0	0.85	1.26	2.07

Values are in millimeter

Table S7: Growth table: cartilage thickness at the trochlea of the knee in males

Year	5% percentile	50% percentile	95% percentile
2.0	3.13	4.14	5.10
2.5	2.97	3.97	4.89
3.0	2.85	3.82	4.72
3.5	2.77	3.70	4.58
4.0	2.73	3.59	4.47
4.5	2.73	3.51	4.40
5.0	2.76	3.45	4.35
5.5	2.80	3.41	4.33
6.0	2.85	3.39	4.31
6.5	2.88	3.37	4.30
7.0	2.90	3.37	4.29
7.5	2.90	3.36	4.28
8.0	2.88	3.36	4.26
8.5	2.83	3.35	4.24
9.0	2.77	3.33	4.20
9.5	2.68	3.30	4.16
10.0	2.57	3.26	4.11
10.5	2.44	3.22	4.05
11.0	2.30	3.17	3.98
11.5	2.16	3.11	3.90
12.0	2.03	3.04	3.81
12.5	1.92	2.97	3.72
13.0	1.83	2.89	3.61
13.5	1.77	2.82	3.50
14.0	1.73	2.75	3.38
14.5	1.71	2.71	3.25
15.0	1.72	2.68	3.15

15.5	1.74	2.67	3.10
16.0	1.80	2.67	3.13
16.5	1.87	2.70	3.27
17.0	1.97	2.74	3.51

Values are in millimeter

Table S8: Growth table: cartilage thickness at the trochlea of the knee in females

Year	5% percentile	50% percentile	95% percentile
2.0	2.49	3.49	4.47
2.5	2.42	3.36	4.32
3.0	2.39	3.29	4.20
3.5	2.39	3.25	4.13
4.0	2.40	3.22	4.09
4.5	2.40	3.20	4.09
5.0	2.40	3.17	4.11
5.5	2.40	3.14	4.15
6.0	2.39	3.11	4.19
6.5	2.37	3.07	4.22
7.0	2.35	3.03	4.22
7.5	2.32	2.98	4.21
8.0	2.28	2.94	4.18
8.5	2.24	2.89	4.13
9.0	2.19	2.84	4.06
9.5	2.14	2.78	3.99
10.0	2.08	2.72	3.90
10.5	2.02	2.66	3.81
11.0	1.95	2.60	3.72
11.5	1.89	2.53	3.63
12.0	1.82	2.47	3.53
12.5	1.77	2.42	3.43
13.0	1.72	2.37	3.33
13.5	1.67	2.33	3.23
14.0	1.64	2.29	3.15
14.5	1.61	2.26	3.07
15.0	1.58	2.23	3.01

15.5	1.57	2.20	2.96
16.0	1.56	2.18	2.92
16.5	1.56	2.15	2.9
17.0	1.57	2.13	2.89

Values are in millimeter

Table S9: Growth table: cartilage thickness at the talar dome in males

Year	5% percentile	50% percentile	95% percentile
2.0	1.23	2.28	4.19
2.5	1.21	2.28	3.95
3.0	1.18	2.26	3.72
3.5	1.16	2.21	3.52
4.0	1.13	2.13	3.34
4.5	1.10	2.03	3.17
5.0	1.07	1.92	3.03
5.5	1.04	1.80	2.90
6.0	1.01	1.69	2.78
6.5	0.99	1.61	2.68
7.0	0.97	1.54	2.58
7.5	0.95	1.49	2.50
8.0	0.93	1.47	2.42
8.5	0.92	1.47	2.36
9.0	0.91	1.48	2.30
9.5	0.90	1.49	2.26
10.0	0.89	1.51	2.22
10.5	0.89	1.52	2.20
11.0	0.89	1.52	2.18
11.5	0.89	1.51	2.17
12.0	0.90	1.50	2.16
12.5	0.91	1.48	2.15
13.0	0.92	1.47	2.13
13.5	0.93	1.45	2.11
14.0	0.95	1.43	2.09
14.5	0.96	1.40	2.07
15.0	0.98	1.38	2.05

15.5	0.98	1.35	2.02
16.0	0.97	1.32	1.99
16.5	0.93	1.29	1.96
17.0	0.88	1.25	1.93

Values are in millimeter

Table S10: Growth table: cartilage thickness at the talar dome in females

Year	5% percentile	50% percentile	95% percentile
2.0	1.23	2.28	4.19
2.5	1.21	2.28	3.95
3.0	1.18	2.26	3.72
3.5	1.16	2.21	3.52
4.0	1.13	2.13	3.34
4.5	1.10	2.03	3.17
5.0	1.07	1.92	3.03
5.5	1.04	1.80	2.90
6.0	1.01	1.69	2.78
6.5	0.99	1.61	2.68
7.0	0.97	1.54	2.58
7.5	0.95	1.49	2.50
8.0	0.93	1.47	2.42
8.5	0.92	1.47	2.36
9.0	0.91	1.48	2.30
9.5	0.90	1.49	2.26
10.0	0.89	1.51	2.22
10.5	0.89	1.52	2.20
11.0	0.89	1.52	2.18
11.5	0.89	1.51	2.17
12.0	0.90	1.50	2.16
12.5	0.91	1.48	2.15
13.0	0.92	1.47	2.13
13.5	0.93	1.45	2.11
14.0	0.95	1.43	2.09
14.5	0.96	1.40	2.07
15.0	0.98	1.38	2.05

15.5	0.98	1.35	2.02
16.0	0.97	1.32	1.99
16.5	0.93	1.29	1.96
17.0	0.88	1.25	1.93

Values are in millimeter

Table S11: Growth table: cartilage thickness at the head of 1st metatarsal bone in males

Year	5% percentile	50% percentile	95% percentile
2.0	1.86	3.21	5.44
2.5	1.86	3.21	5.13
3.0	1.86	3.18	4.84
3.5	1.86	3.13	4.58
4.0	1.86	3.06	4.34
4.5	1.85	2.98	4.12
5.0	1.83	2.90	3.92
5.5	1.80	2.81	3.75
6.0	1.77	2.71	3.60
6.5	1.72	2.61	3.47
7.0	1.67	2.50	3.36
7.5	1.61	2.39	3.27
8.0	1.55	2.28	3.17
8.5	1.47	2.18	3.08
9.0	1.39	2.07	2.99
9.5	1.30	1.97	2.89
10.0	1.22	1.88	2.80
10.5	1.13	1.79	2.70
11.0	1.04	1.70	2.61
11.5	0.95	1.62	2.51
12.0	0.88	1.54	2.42
12.5	0.82	1.46	2.32
13.0	0.77	1.39	2.23
13.5	0.73	1.32	2.13
14.0	0.70	1.26	2.03
14.5	0.68	1.20	1.94
15.0	0.68	1.15	1.86

15.5	0.68	1.12	1.80
16.0	0.70	1.10	1.80
16.5	0.73	1.10	1.85
17.0	0.77	1.12	1.95

Values are in millimeter

Table S12: Growth table: cartilage thickness at the head of 1st metatarsal bone in females

Year	5% percentile	50% percentile	95% percentile
2.0	1.68	2.81	4.55
2.5	1.71	2.74	4.35
3.0	1.72	2.66	4.17
3.5	1.71	2.58	3.98
4.0	1.69	2.50	3.81
4.5	1.66	2.42	3.63
5.0	1.61	2.34	3.46
5.5	1.54	2.24	3.29
6.0	1.46	2.15	3.13
6.5	1.36	2.05	2.97
7.0	1.26	1.95	2.82
7.5	1.15	1.85	2.67
8.0	1.05	1.76	2.53
8.5	0.96	1.67	2.40
9.0	0.88	1.59	2.28
9.5	0.82	1.52	2.19
10.0	0.76	1.46	2.11
10.5	0.71	1.41	2.06
11.0	0.66	1.37	2.02
11.5	0.62	1.33	2.01
12.0	0.58	1.31	2.00
12.5	0.54	1.29	2.00
13.0	0.51	1.27	2.00
13.5	0.48	1.25	2.00
14.0	0.47	1.21	2.01
14.5	0.47	1.16	2.01
15.0	0.48	1.09	2.02

15.5	0.51	1.03	2.03
16.0	0.55	1.00	2.04
16.5	0.60	1.01	2.06
17.0	0.67	1.07	2.09

Values are in millimeter

Table S13: Growth table: cartilage thickness at the head of 2nd metacarpal bone in males

Year	5% percentile	50% percentile	95% percentile
2.0	1.85	3.78	6.15
2.5	1.71	3.36	5.44
3.0	1.60	3.02	4.72
3.5	1.53	2.73	4.05
4.0	1;49	2.52	3.51
4.5	1.45	2.37	3.15
5.0	1.41	2.26	2.95
5.5	1.38	2.19	2.85
6.0	1.35	2.13	2.81
6.5	1.31	2.08	2.80
7.0	1.27	2.02	2.80
7.5	1.23	1.95	2.80
8.0	1.19	1.89	2.79
8.5	1.15	1.82	2.76
9.0	1.11	1.75	2.71
9.5	1.06	1.67	2.65
10.0	1.02	1.60	2.57
10.5	0.97	1.53	2.47
11.0	0.93	1.47	2.36
11.5	0.89	1.41	2.24
12.0	0.85	1.35	2.10
12.5	0.81	1.30	1.97
13.0	0.77	1.24	1.83
13.5	0.74	1.20	1.70
14.0	0.71	1.15	1.60
14.5	0.70	1.10	1.51
15.0	0.69	1.06	1.45

15.5	0.70	1.03	1.41
16.0	0.71	1.00	1.40
16.5	0.74	0.99	1.41
17.0	0.77	0.99	1.45

Values are in millimeter

Table S14: Growth table: cartilage thickness at the head of 2nd metacarpal bone in females

Year	5% percentile	50% percentile	95% percentile
2.0	1.29	2.70	4.69
2.5	1.16	2.36	4.16
3.0	1.17	2.15	3.69
3.5	1.26	2.02	3.30
4.0	1.35	1.95	2.98
4.5	1.40	1.88	2.72
5.0	1.39	1.82	2.52
5.5	1.34	1.76	2.38
6.0	1.28	1.71	2.29
6.5	1.21	1.66	2.23
7.0	1.16	1.61	2.20
7.5	1.10	1.56	2.19
8.0	1.06	1.50	2.19
8.5	1.02	1.45	2.20
9.0	0.98	1.40	2.20
9.5	0.95	1.35	2.21
10.0	0.92	1.30	2.20
10.5	0.89	1.25	2.20
11.0	0.86	1.20	2.18
11.5	0.84	1.16	2.16
12.0	0.81	1.13	2.12
12.5	0.79	1.09	2.06
13.0	0.77	1.06	1.98
13.5	0.75	1.04	1.89
14.0	0.73	1.01	1.77
14.5	0.71	0.99	1.64
15.0	0.69	0.98	1.51

15.5	0.66	0.96	1.39
16.0	0.63	0.95	1.32
16.5	0.60	0.94	1.30
17.0	0.55	0.94	1.36

Values are in millimeter

Table S15: Growth table: distention at the acetabulofemoral recess in males and females

Year	5% percentile	50% percentile	95% percentile
2.0	1.23	2.74	4.95
2.5	1.30	2.92	5.27
3.0	1.37	3.07	5.54
3.5	1.43	3.19	574
4.0	1.47	3.27	5.88
4.5	1.51	3.33	5.96
5.0	1.54	3.35	6.00
5.5	1.56	3.35	6.00
6.0	1.57	3.32	6.00
6.5	1.57	3.27	6.00
7.0	1.56	3.20	6.00
7.5	1.54	3.13	6.00
8.0	1.52	3.05	6.02
8.5	1.49	2.98	6.03
9.0	1.46	2.93	6.06
9.5	1.44	2.90	6.08
10.0	1.43	2.90	6.12
10.5	1.44	2.94	6.15
11.0	1.47	3.02	6.20
11.5	1.52	3.14	6.24
12.0	1.58	3.29	6.30
12.5	1.67	3.48	6.35
13.0	1.77	3.70	6.42
13.5	1.90	3.92	6.49
14.0	2.04	4.13	6.56
14.5	2.20	4.31	6.64
15.0	2.33	4.45	6.72

15.5	2.40	4.55	6.81
16.0	2.36	4.60	6.90
16.5	2.18	4.58	7.00
17.0	2.20	4.50	7.10

Values are in millimeter

Table S16: Growth table: distention at the recess of MTP1 joint in males and females

Year	5% percentile	50% percentile	95% percentile
2.0	0.00	0.02	0.52
2.5	0.00	0.05	0.53
3.0	0.02	0.12	0.50
3.5	0.00	0.18	0.56
4.0	0.00	0.29	0.56
4.5	0.00	0.33	0.58
5.0	0.00	0.36	0.59
5.5	0.00	0.38	0.61
6.0	0.00	0.38	0.63
6.5	0.00	0.39	0.62
7.0	0.00	0.42	0.68
7.5	0.00	0.44	0.82
8.0	0.00	0.45	1.05
8.5	0.00	0.45	1.08
9.0	0.00	0.49	1.13
9.5	0.00	0.51	1.16
10.0	0.00	0.53	1.36
10.5	0.00	0.53	1.39
11.0	0.00	0.54	1.49
11.5	0.00	0.56	1.64
12.0	0.00	0.61	1.82
12.5	0.00	0.63	1.92
13.0	0.00	0.64	1.98
13.5	0.07	0.67	2.03
14.0	0.07	0.69	2.12
14.5	0.02	0.81	2.18
15.0	0.00	0.86	2.05

15.5	0.00	1.03	2.06
16.0	0.03	1.06	2.08
16.5	0.00	1.06	2.08
17.0	0.00	1.07	2.09

Values are in millimeter; MTP1: first metatarsophalangeal joint

Table S17: Growth table: tendon diameter of the biceps tendon in males and females

Year	5% percentile	50% percentile	95% percentile
2.0	2.74	3.68	5.57
2.5	2.86	3.9	5.85
3.0	2.97	4.1	6.11
3.5	3.08	4.3	6.36
4.0	3.19	4.48	6.60
4.5	3.28	4.65	6.81
5.0	3.37	4.81	7.02
5.5	3.46	4.95	7.20
6.0	3.53	5.09	7.37
6.5	3.61	5.21	7.52
7.0	3.67	5.33	7.66
7.5	3.73	5.43	7.78
8.0	3.78	5.52	7.88
8.5	3.83	5.59	7.97
9.0	3.87	5.66	8.05
9.5	3.91	5.71	8.1
10.0	3.95	5.76	8.14
10.5	3.98	5.79	8.17
11.0	4.01	5.81	8.18
11.5	4.04	5.82	8.18
12.0	4.06	5.84	8.18
12.5	4.09	5.85	8.18
13.0	4.11	5.86	8.18
13.5	4.13	5.87	8.18
14.0	4.14	5.87	8.18
14.5	4.16	5.88	8.18
15.0	4.17	5.89	8.18

15.5	4.18	5.89	8.18
16.0	4.19	5.89	8.18
16.5	4.19	5.90	8.18
17.0	4.20	5.90	8.18

Values are in millimeter

Table S18: Growth table: diameter of the patellar tendon in males and females

Year	5% percentile	50% percentile	95% percentile
2.0	11.86	15.40	18.95
2.5	11.40	16.16	19.91
3.0	12.93	16.89	20.83
3.5	13.44	17.60	21.74
4.0	13.94	18.28	22.62
4.5	14.41	18.94	23.48
5.0	14.87	19.57	24.32
5.5	15.31	20.18	25.13
6.0	15.73	20.77	25.92
6.5	16.14	21.33	26.69
7.0	16.52	21.86	27.43
7.5	16.89	22.38	28.15
8.0	17.24	22.86	28.85
8.5	17.58	23.33	29.52
9.0	17.89	23.77	30.17
9.5	18.19	24.18	30.80
10.0	18.47	24.57	31.40
10.5	18.73	24.94	31.98
11.0	18.98	25.28	32.53
11.5	19.20	25.60	33.07
12.0	19.41	25.89	33.58
12.5	19.60	26.16	34.06
13.0	19.78	26.40	34.53
13.5	19.93	26.62	34.97
14.0	20.07	26.82	35.38
14.5	20.19	26.99	35.78
15.0	20.29	27.13	36.15

15.5	20.38	27.26	36.50
16.0	20.44	27.35	36.82
16.5	20.49	27.43	37.12
17.0	20.52	27.48	37.40

Values are in millimeter

Table S19: Growth table: diameter of the extensor digitorum communis tendon in males and females

Year	5% percentile	50% percentile	95% percentile
2.0	5.14	8.30	11.39
2.5	5.35	8.68	11.91
3.0	5.55	9.03	12.38
3.5	5.76	9.36	12.78
4.0	5.96	9.66	13.12
4.5	6.16	9.93	13.40
5.0	6.36	10.18	13.63
5.5	6.55	10.41	13.81
6.0	6.75	10.60	13.97
6.5	6.94	10.78	14.09
7.0	7.13	10.93	14.20
7.5	7.32	11.07	14.31
8.0	7.51	11.20	14.43
8.5	7.69	11.33	14.57
9.0	7.88	11.46	14.73
9.5	8.06	11.59	14.91
10.0	8.24	11.71	15.11
10.5	8.42	11.84	15.33
11.0	8.59	11.98	15.57
11.5	8.77	12.11	15.84
12.0	8.94	12.25	16.13
12.5	9.11	12.40	16.43
13.0	9.28	12.54	16.76
13.5	9.45	12.69	17.11
14.0	9.62	12.84	17.48
14.5	9.78	12.99	17.88
15.0	9.94	13.15	18.29

15.5	10.10	13.31	18.73
16.0	10.26	13.47	19.19
16.5	10.42	13.64	19.66
17.0	10.57	13.80	20.16

Values are in millimeter

Table S20: Growth table: diameter of the extensor carpi ulnaris tendon in males and females

Year	5% percentile	50% percentile	95% percentile
2.0	2.54	4.46	5.70
2.5	2.69	4.66	5.97
3.0	2.84	4.85	6.24
3.5	2.99	5.03	6.50
4.0	3.13	5.21	6.76
4.5	3.27	5.38	7.00
5.0	3.40	5.54	7.24
5.5	3.53	5.70	7.48
6.0	3.65	5.86	7.70
6.5	3.77	6.00	7.92
7.0	3.88	6.14	8.13
7.5	3.99	6.28	8.33
8.0	4,10	6.40	8.53
8.5	4,20	6.52	8.72
9.0	4.29	6.64	8.90
9.5	4.38	6.75	9.07
10.0	4.47	6.85	9.24
10.5	4.55	6.95	9.40
11.0	4.63	7.04	9.55
11.5	4.70	7.12	9.70
12.0	4.77	7.20	9.84
12.5	4.83	7.27	9.97
13.0	4.89	7.34	10.09
13.5	4.94	7.39	10.21
14.0	4.99	7.45	10.32
14.5	5.03	7.49	10.42
15.0	5.07	7.53	10.51

15.5	5.11	7.57	10.60
16.0	5.14	7.59	10.68
16.5	5.16	7.62	10.76
17.0	5.19	7.63	10.82

Values are in millimeter

Table S21: Growth table: diameter of the flexor digitorum (superficial and profound) tendon of the 2nd finger in males and females

Year	5% percentile	50% percentile	95% percentile
2.0	3.63	5.20	6.95
2.5	3.82	5.48	6.98
3.0	3.98	5.72	7.02
3.5	4.12	5.90	7.05
4.0	4.23	6.03	7.09
4.5	4.31	6.11	7.13
5.0	4.37	6.15	7.18
5.5	4.41	6.17	7.24
6.0	4.43	6.19	7.31
6.5	4.43	6.20	7.39
7.0	4.43	6.22	7.48
7.5	4.44	6.26	7.57
8.0	4.46	6.30	7.67
8.5	4.50	6.35	7.78
9.0	4.56	6.42	7.90
9.5	4.65	6.50	8.02
10.0	4.76	6.59	8.16
10.5	4.90	6.69	8.30
11.0	5.05	6.81	8.44
11.5	5.22	6.93	8.58
12.0	5.38	7.07	8.71
12.5	5.54	7.22	8.81
13.0	5.70	7.37	8.90
13.5	5.84	7.50	8.96
14.0	5.97	7.61	9.01
14.5	6.08	7.69	9.04
15.0	6.18	7.74	9.05

15.5	6.26	7.76	9.06
16.0	6.32	7.78	9.06
16.5	6.37	7.79	9.06
17.0	6.40	7.80	9.06

Values are in millimeter

FIGURE S1

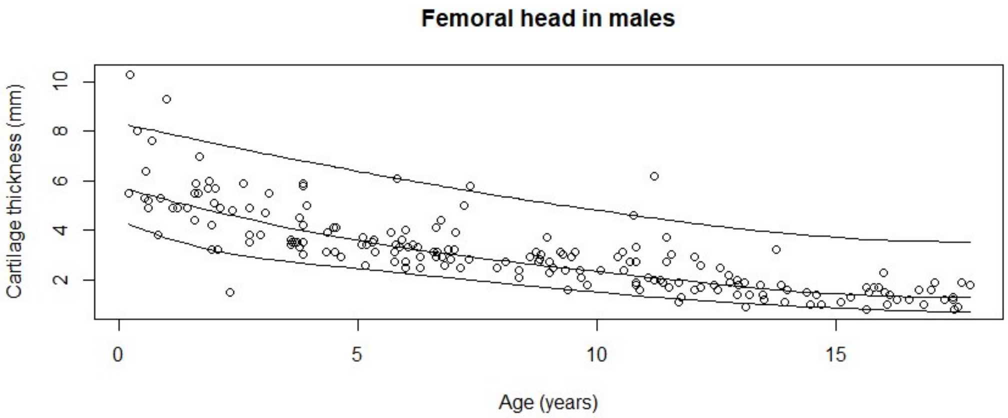
Femoral head in males
Cartilage thickness (mm)
2
4
6
8
10
0
5
10
15
Age (years)

FIGURE S2

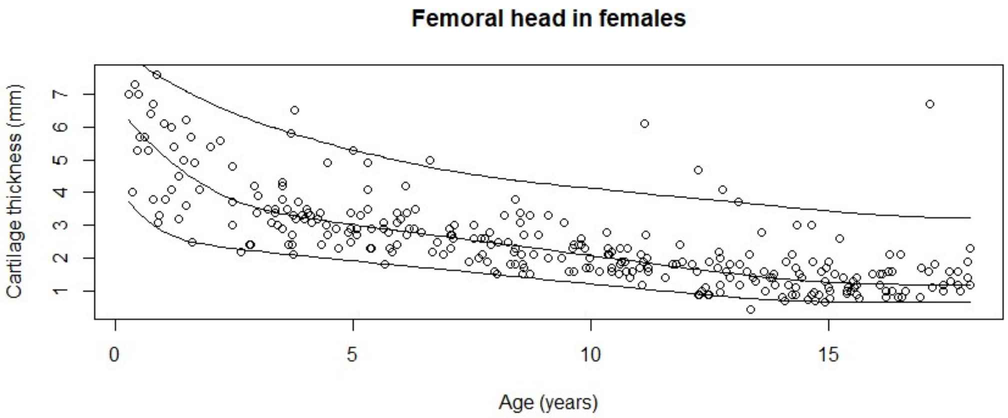

Femoral head in females
Cartilage thickness (mm)
1
2
3
4
5
6
7
0
5
10
15
Age (years)

FIGURE S3

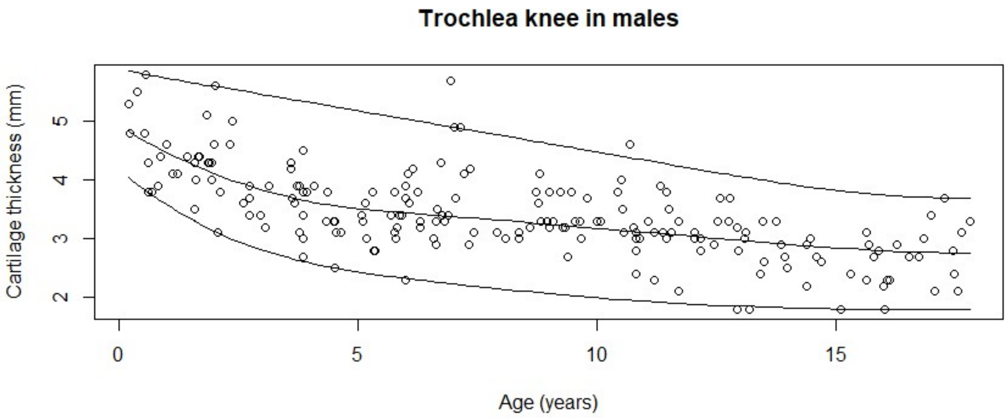

Trochlea knee in males
Cartilage thickness (mm)
2
3
4
5
0
5
10
15
Age (years)

FIGURE S4

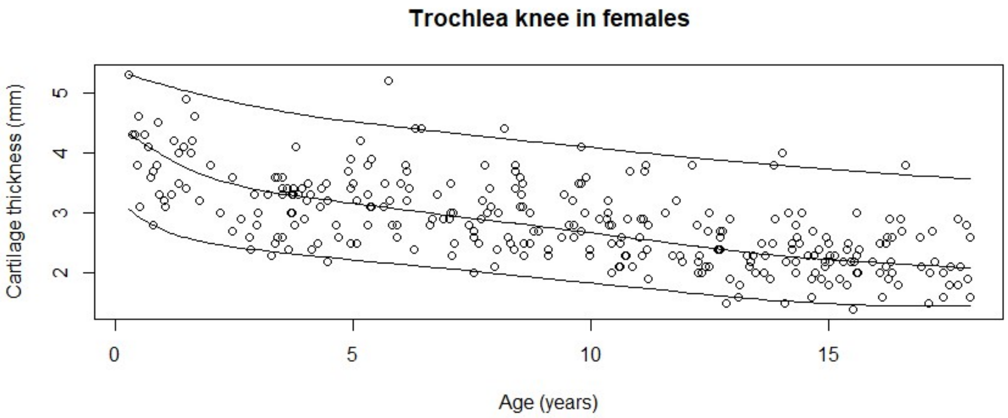

Trochlea knee in females
Cartilage thickness (mm)
2
3
4
5
0
5
10
15
Age (years)

FIGURE S5

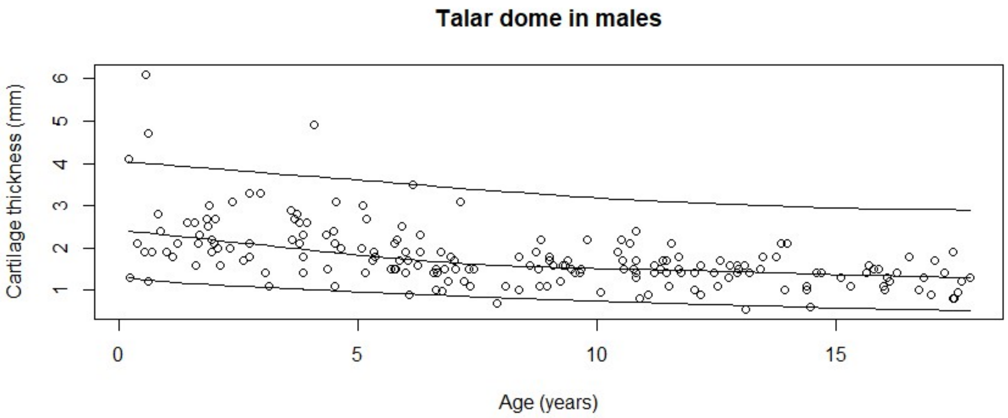

Talar dome in males
Cartilage thickness (mm)
1
2
3
4
5
6
0
5
10
15
Age (years)

FIGURE S6

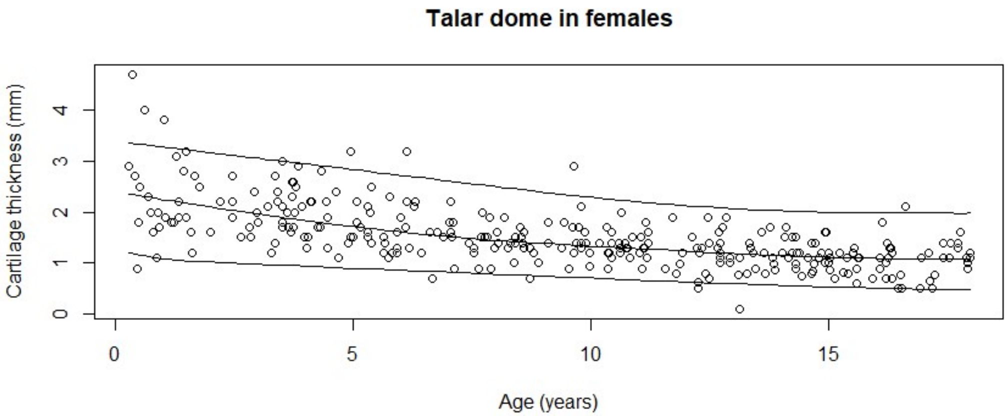
Talar dome in females
Cartilage thickness (mm)
0
1
2
3
4
0
5
10
15
Age (years)

FIGURE S7

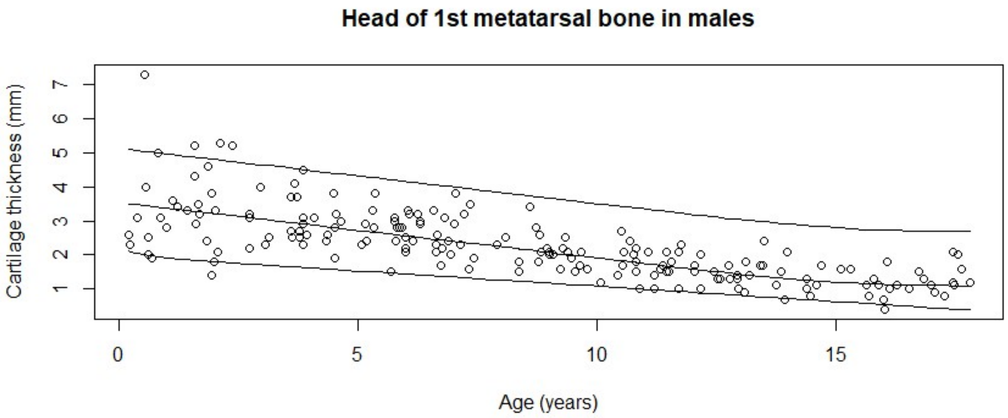
Head of 1st metatarsal bone in males
Cartilage thickness (mm)
1
2
3
4
5
6
7
0
5
10
15
Age (years)

FIGURE S8

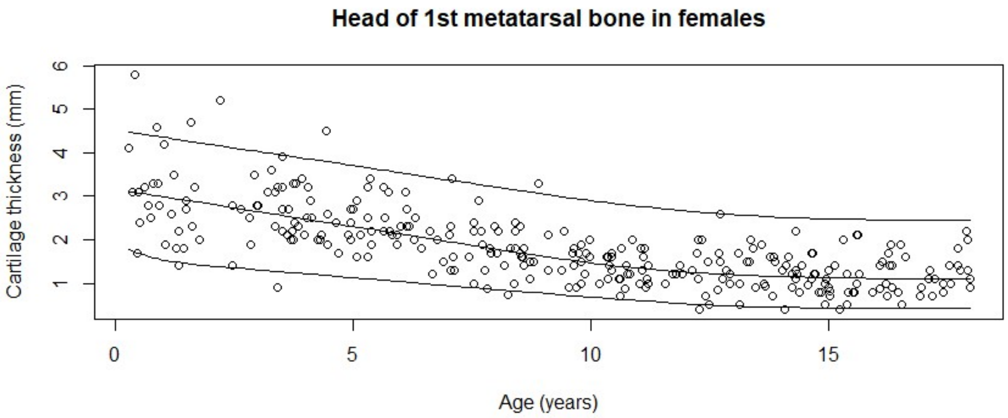

Head of 1st metatarsal bone in females
Cartilage thickness (mm)
1
2
3
4
5
6
0
5
10
15
Age (years)

FIGURE S9

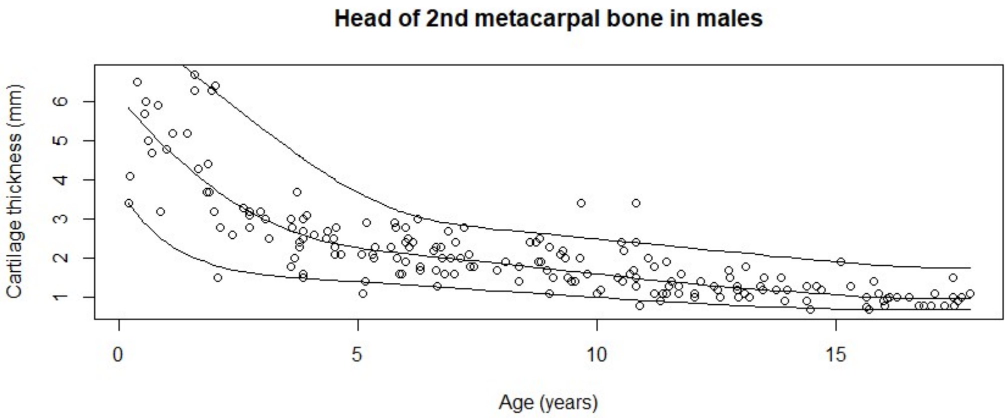
Head of 2nd metacarpal bone in males
Cartilage thickness (mm)
1
2
3
4
5
6
0
5
10
15
Age (years)

FIGURE S10

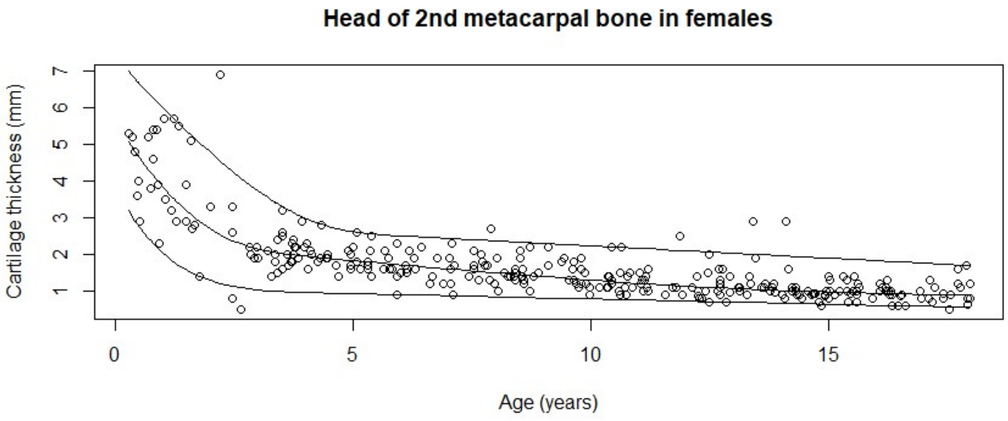

Head of 2nd metacarpal bone in females
Cartilage thickness (mm)
1
2
3
4
5
6
7
0
5
10
15
Age (years)

FIGURE S11

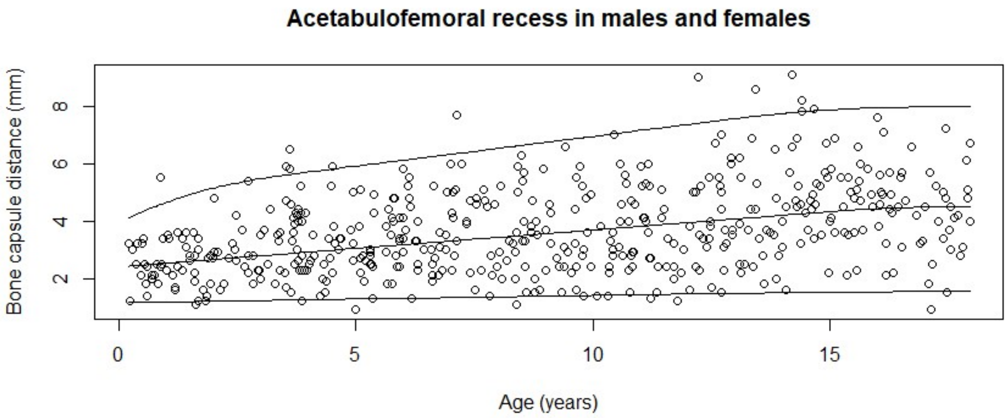

Acetabulofemoral recess in males and females
Bone capsule distance (mm)
2
4
6
8
0
5
10
15
Age (years)

FIGURE S12

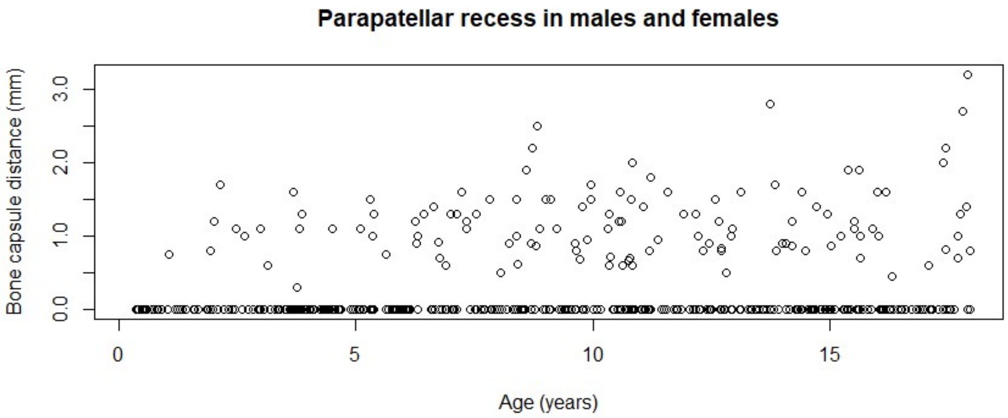
Parapatellar recess in males and females
Bone capsule distance (mm)
0.0
1.0
2.0
3.0
0
5
10
15
Age (years)

FIGURE S13

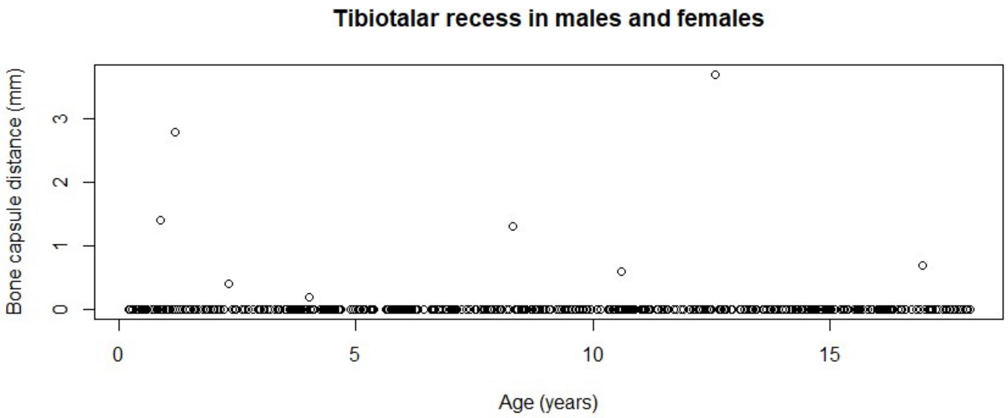

Tibiotalar recess in males and females
Bone capsule distance (mm)
0
1
2
3
0
5
10
15
Age (years)

FIGURE S14

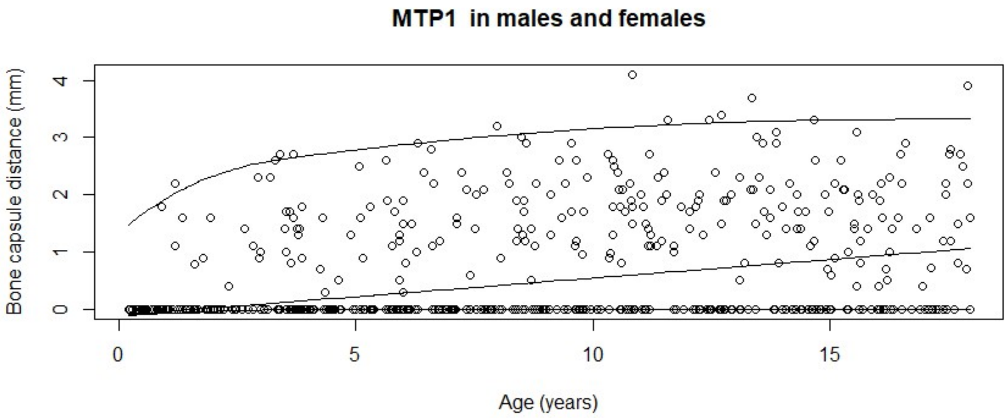

MTP1 in males and females
Bone capsule distance (mm)
0
1
2
3
4
0
5
10
15
Age (years)

FIGURE S15

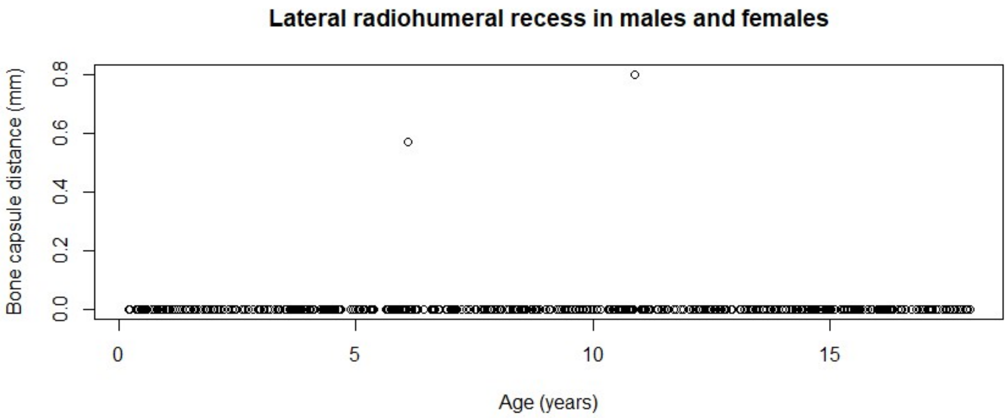
Lateral radiohumeral recess in males and females
Bone capsule distance (mm)
0.0
0.2
0.4
0.6
0.8
0
5
10
15
Age (years)

FIGURE S16

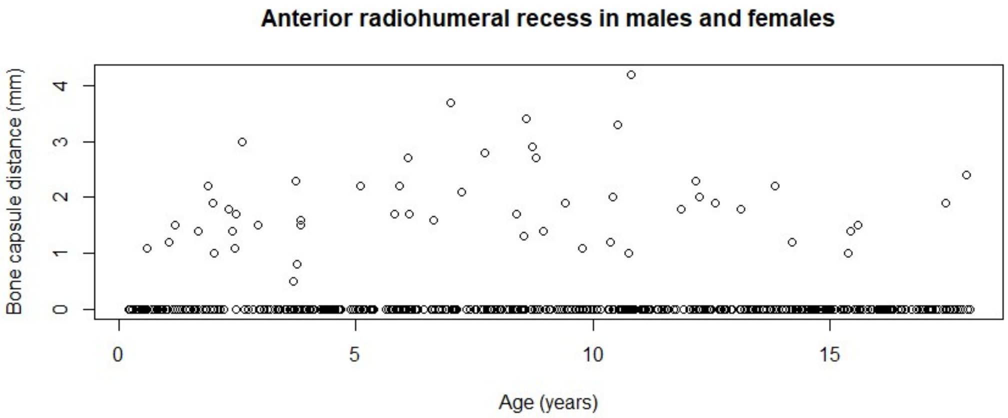

Anterior radiohumeral recess in males and females
Bone capsule distance (mm)
0
1
2
3
4
0
5
10
15
Age (years)

FIGURE S17

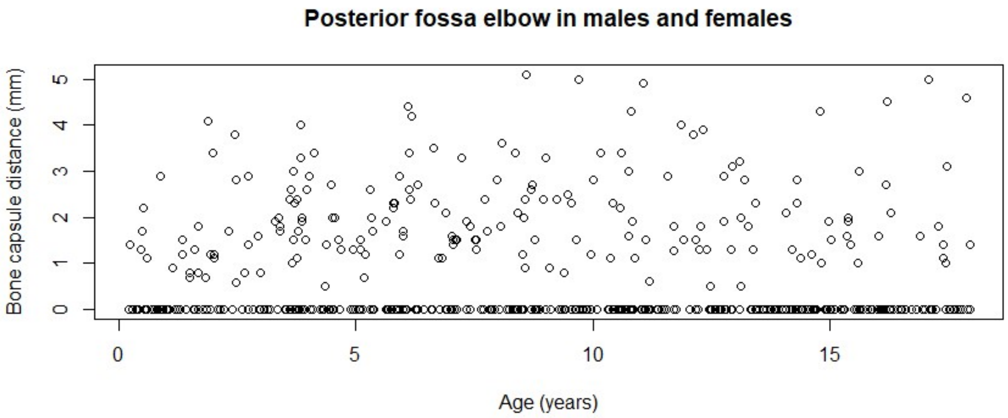
Posterior fossa elbow in males and females
Bone capsule distance (mm)
0
1
2
3
4
5
0
5
10
15
Age (years)

FIGURE S18

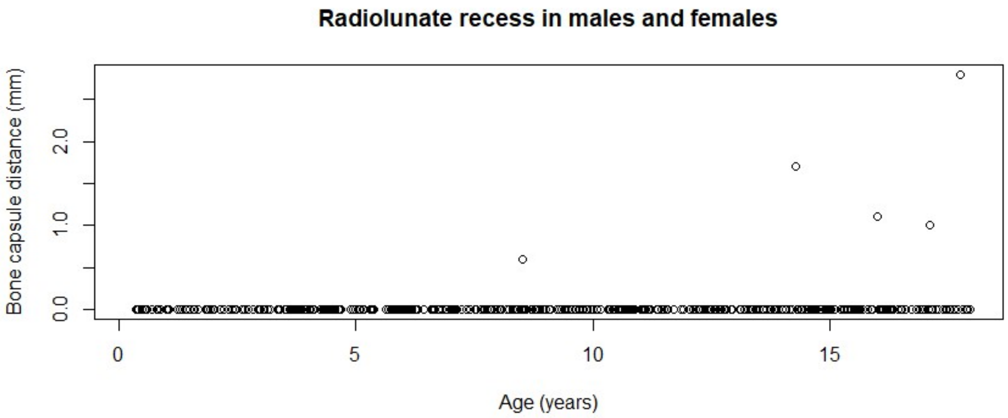

Radiolunate recess in males and females
Bone capsule distance (mm)
0.0
1.0
2.0
0
5
10
15
Age (years)

FIGURE S19

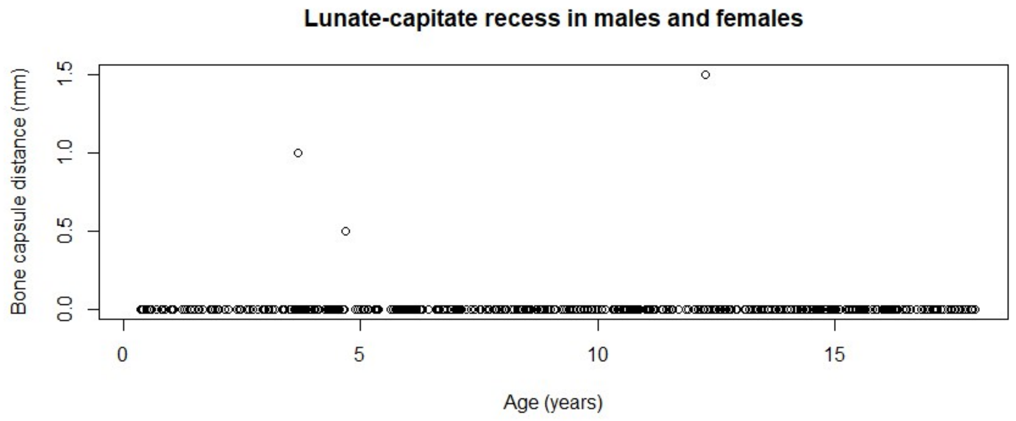

Lunate-capitate recess in males and females
Bone capsule distance (mm)
0.0
0.5
1.0
1.5
0
5
10
15
Age (years)

FIGURE S20

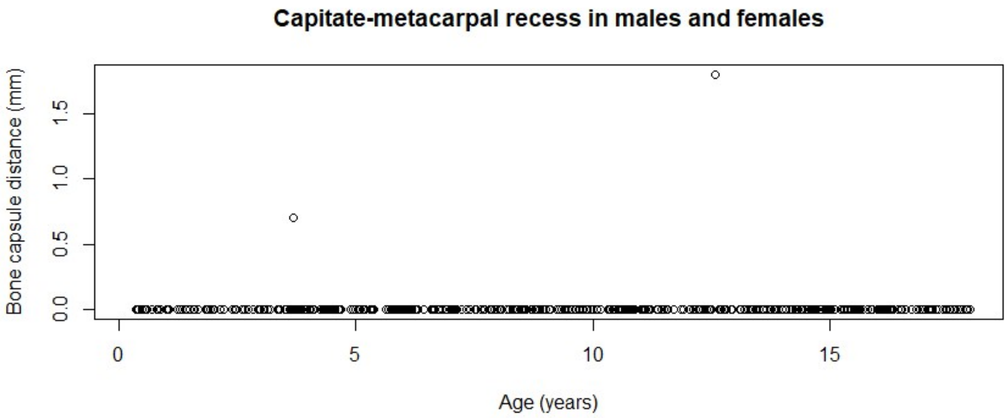

Capitate-metacarpal recess in males and females
Bone capsule distance (mm)
0.0
0.5
1.0
1.5
0
5
10
15
Age (years)

FIGURE S21

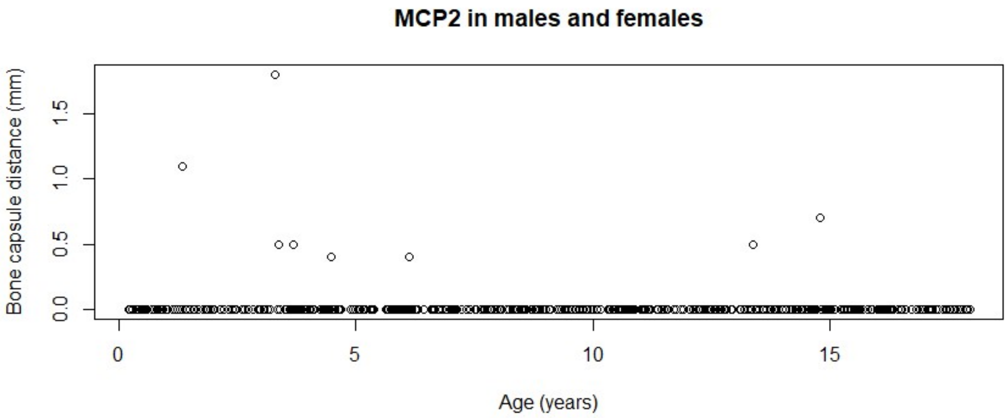
MCP2 in males and females
Bone capsule distance (mm)
0.0
0.5
1.0
1.5
0
5
10
15
Age (years)

FIGURE S22

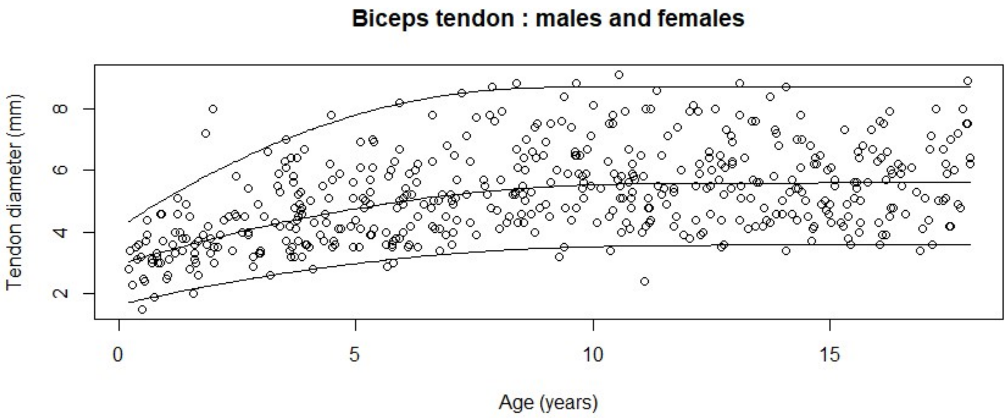

Biceps tendon : males and females
Tendon diameter (mm)
2
4
6
8
0
5
10
15
Age (years)

FIGURE S23

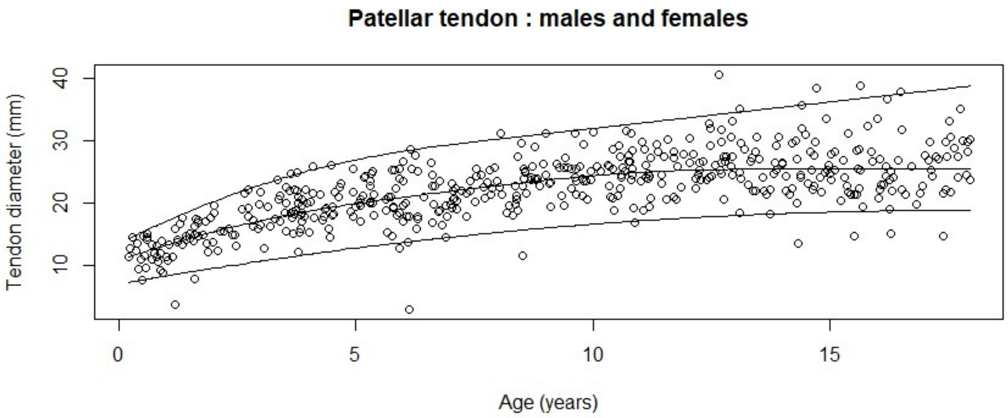
Patellar tendon : males and females
Tendon diameter (mm)
10
20
30
40
0
5
10
15
Age (years)

FIGURE S24

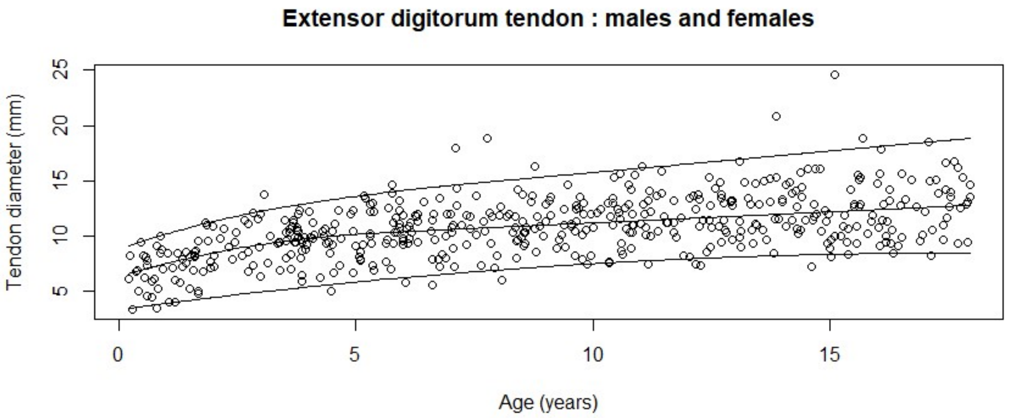

Extensor digitorum tendon : males and females
Tendon diameter (mm)
25
20
15
10
5
0
5
10
15
Age (years)

FIGURE S25

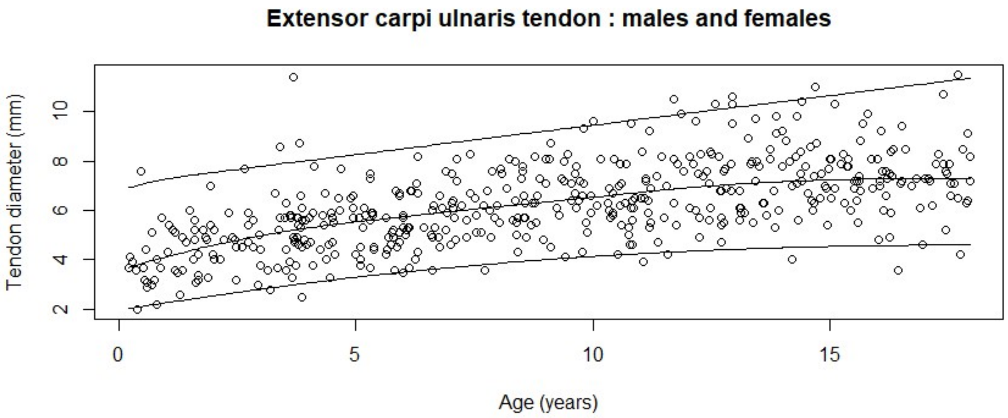

Extensor carpi ulnaris tendon : males and females
Tendon diameter (mm)
2
4
6
8
10
0
5
10
15
Age (years)

FIGURE S26

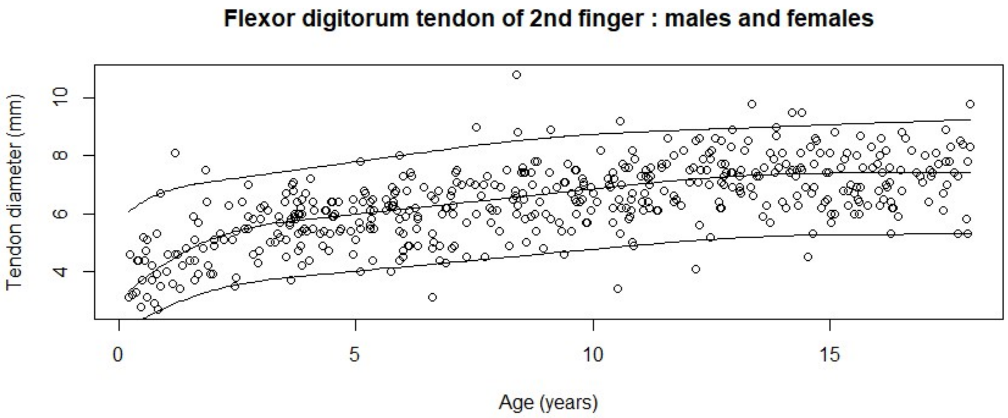
Flexor digitorum tendon of 2nd finger : males and females
Tendon diameter (mm)
4
6
8
10
0
5
10
15
Age (years)
